# The BRCA1-A complex restricts replication fork reversal-dependent DNA repair in ATM deficient cells

**DOI:** 10.1038/s41467-026-75271-7

**Published:** 2026-07-04

**Authors:** Arindam Datta, Jessica Jackson, Yaroslav I. Morozov, Jinghan Qiu, Alessandro Vindigni, Roger A. Greenberg

**Affiliations:** 1 Department of Cancer Biology, Penn Center for Genome Integrity, Basser Center for BRCA, Perelman School of Medicine, University of Pennsylvania, Philadelphia, PA USA; 2 Division of Oncology, Washington University, St. Louis, MO USA

**Keywords:** Genetics, Chromosomes

## Abstract

Ataxia Telangiectasia Mutated (ATM) kinase deficiency results in cancer susceptibility and drug hypersensitivity. Deficiency in either the BRCA1 interacting A complex or XRCC4/Ligase 4 confers resistance to Topoisomerase I or PARP1 inhibitors in ATM-deficient cells. This suggests that BRCA1-A directs toxicity to fork-damaging agents in ATM mutated cells via illegitimate end-joining. Here, we show that ATM inhibition triggers combined SUMO and ubiquitin mediated BRCA1-A damaged fork recognition to restrict end-resection and cause Topoisomerase I inhibitor hypersensitivity. BRCA1-A deficient cells display elevated chromatin accessibility and nuclease activity at damaged forks, coupled with restored resection and drug resistance. Electron microscopy evidence demonstrates that ATM inhibition prevents replication fork reversal, which is restored by BRCA1-A loss to generate substrates for end resection. These findings reveal that BRCA1-A enforces a restrictive chromatin state to suppress the genesis of resection substrates, implicating fork reversal as a key determinant of chemotherapy response in ATM deficient cells.

## Abstract (web summary)

ATM kinase activity is required for cellular survival to replication fork damaging chemotherapies. Here the authors show that deficiency in the BRCA1-A complex promotes chemotherapy resistance of ATM mutated cells by restoring fork reversal to allow homologous recombination repair.

## Introduction

Pathogenic mutations to the *BRCA1* or *ATM* genes compromise homologous recombination (HR) and predispose to breast and ovarian cancers^1–9^. BRCA1 is directly implicated in the multi-step process of HR by promoting end-resection and assembly of the RAD51 filament. Accordingly, BRCA1 mutant cancers display genomic alterations that are characteristic of HR deficiency, and either germline or somatic mutations in BRCA1 predict positive responses to PARP inhibitor (PARPi) or platinum-based chemotherapy^9–11^. Moreover, restoration of HR by reversion mutations or loss of 53BP1-Shieldin yields PARPi resistance in BRCA1 mutant cancer cells^11,12^. Germline ATM deficiency produces profound radiosensitivity, developmental abnormalities, and cancer susceptibility^5^. ATM phosphorylates more than 1000 substrates in response to ionizing radiation (IR), including factors that promote either HR or nonhomologous end-joining (NHEJ)^13^. BRCA1 and many of its interacting partners are confirmed substrates of ATM kinase activity following DNA damage. Despite these commonalities, ATM deficiency differs from loss of BRCA1 in several respects. This includes a different spectrum of cancer susceptibility and developmental abnormalities^5,14^. These observations are consistent with both shared and distinct aspects of ATM and BRCA1 in response to DNA lesions.

BRCA1 associates stoichiometrically with BARD1 through an amino-terminal interaction^15^. All other BRCA1 interactions are thought to be substoichiometric^16–20^. BRCA1 also interacts with tumor suppressors PALB2 and BRCA2-RAD51 through its coiled coil domain to promote HR^21,22^. The BRCA1 BRCT repeats are the most common site of cancer associated missense mutations in the BRCA1 gene. The BRCT repeats bind phospho-Serine motifs on ABRAXAS, BRIP1, and CtIP to form the mutually exclusive BRCA1- A, B, and C complexes^17,23,24^. Each complex is proposed to direct a different aspect of the damage response. The importance of each interaction with the BRCA1 BRCT repeats remains unknown, however, and interaction defective alleles in either BRIP1 or CtIP do not recapitulate loss of function mutation in either the BRCA1 BRCT repeats or BRIP1 or CtIP knockouts^25–27^.

The BRCA1-A complex is comprised of BRCA1 in association with ABRAXAS, RAP80, BRCC45, MERIT40, and BRCC36. RAP80 contains tandem ubiquitin interaction motifs (UIMs) at its amino terminus that specifically bind lysine63-linked ubiquitin chains (K63-Ub)^18–20^. The UIM domains are preceded by a SUMO-Interaction motif (SIM) domain. Each motif exhibits a dissociation constant of approximately 20 μM for K63-Ub or SUMO-2, respectively, whereas RAP80 binds to hybrid SUMO2-K63-Ub with 80 fold higher affinity (*K*_d_ < 0.2 μM)^28^, raising the possibility that hybrid SUMO-ubiquitin chains provide a key damage recognition element for BRCA1-A. BARD1 targets BRCA1 to damage sites by binding nucleosomes that contain unmethylated H4K20 (H4K20me0) and histone H2AK15-Ub^29,30^. The BRCA1 RING domain interacts with nucleosomes in a ubiquitin-independent manner that allows the BRCA1-BARD1 heterodimer to span di-nucleosomes^31–33^. BARD1 Ankyrin and BRCT interact with H2AK15Ub, H4K20me0 on one nucleosome and the BRCA1 RING domain interacts with the second nucleosome irrespective of methylation and ubiquitin. How BRCA1-A utilizes these multiple recognition elements provided by RAP80 and BRCA1-BARD1 in the DNA damage response is unknown, as is whether they are individually or collectively required to respond to specific lesions.

Despite extensive investigation into its structural and biochemical properties, little is understood regarding how BRCA1-A contributes to the DNA damage response. BRCA1 damage foci are reduced in A-complex knockout cells but this does not result in similar damage response deficits as observed in context of BRCA1 BRCT mutation^34^. A-complex deficiency is well tolerated in mice, which showed minimal to no changes in comparison to wildtype littermates in response to IR and only modest hypersensitivity to replication fork damaging agents^35,36^. Moreover, A-complex deficiency increases end resection and HR, indicating that its involvement in BRCA1 damage localization does not promote HR^37,38^. Recent work speaks to the anti-resection properties of the BRCA1-A complex being a key determinant of chemosensitivity during ATM deficiency. Genome-wide CRISPR-Cas9 screens identified loss of either the A complex or NHEJ as a cause of topoisomerase I inhibitors (TOP1i) and Poly(ADP)ribose polymerase inhibitor (PARPi) resistance specifically in ATM-deficient cancer cells^39^. ATM promotes DNA end resection and HR at replication-associated breaks triggered by TOP1i or PARPi^39–42^. The authors proposed that drug sensitivity in ATM-null cells is due to delayed end resection and HR at broken replication forks, leading to toxic NHEJ^39,43^. These results are consistent with prior demonstrations of an anti-resection function of BRCA1-A^37,38^. A similar synthetic viability interaction occurs upon 53BP1 deletion in BRCA1 mutated cells^44,45^. However, 53BP1 loss did not confer drug resistance in ATM null cells, suggesting BRCA1-A operates in a distinct manner to prevent resection at damaged forks^39^. This further underscores differences between cancers harboring BRCA1 vs. ATM mutations in the response to chemotherapy, thus representing an important knowledge gap in the treatment of a wide spectrum of cancers.

In this study, we report that BRCA1-A blocks end resection by preventing replication fork reversal in ATM-deficient cells. We show that hypersensitivity to fork damaging agents in ATM inhibited cells requires the complete assembly of the BRCA1-A complex and its combined SUMO and ubiquitin interaction. BRCA1 association with ABRAXAS restricted end-resection and conferred hypersensitivity to Top1i in ATM inhibited cells. Electron microscopy (EM) revealed that ATM kinase activity is required for reversal of damaged replication forks in a manner dependent on fork remodeling proteins, whereas A-complex loss restored fork reversal in ATM inhibited cells commensurate with increased chromatin accessibility, end resection, damage recovery, and Top1i resistance. These findings identify BRCA1-A mediated blockade of replication fork reversal as a critical determinant of end resection and response to cancer therapeutics in ATM deficient cells.

## Results

### BRCA1-A suppresses damaged fork resection in ATM-deficient cells

**Fig. 1:**
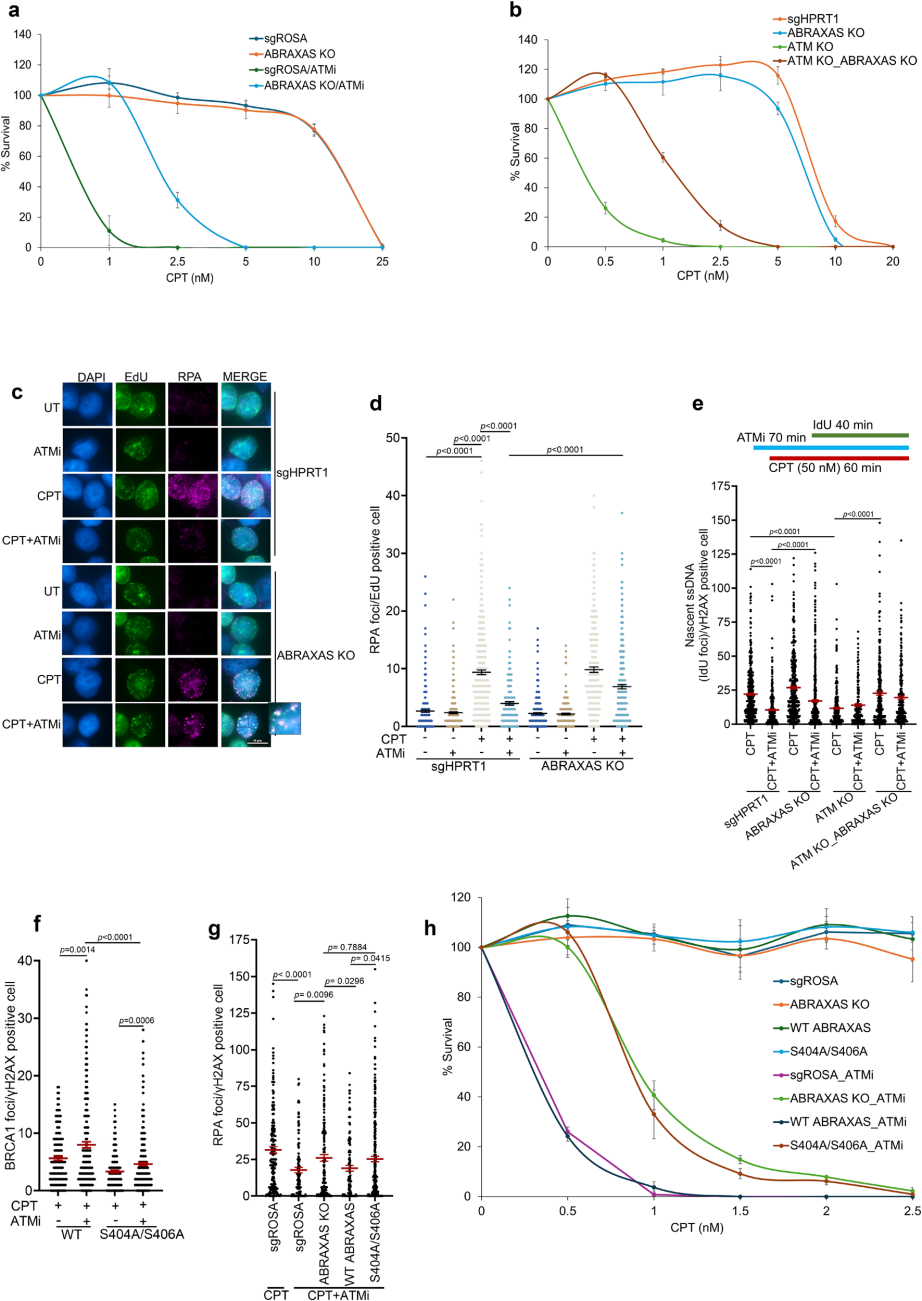
BRCA1 A-complex suppresses damaged fork resection and viability in ATM-deficient cells. **a** Relative colony survival (%) of control (sgROSA) and ABRAXAS KO HEK293T cells treated as indicated. **b** Relative colony survival (%) in the indicated genotypes of HT-29 cells treated as indicated. In **a**, **b**, data represent mean ± SD of *n* = 3 independent experiments. **c** Representative RPA immunofluorescence in the indicated genotypes of HT-29 cells treated with 50 nM CPT + /- 250 nM ATMi for 1 h. Cells were concomitantly labeled with EdU (10 µM) to identify S-phase cells. An inset shows colocalization of RPA signal with EdU spot. Images were acquired with 100X magnification. Scale bar is shown. **d** Scatter plot showing RPA foci number per EdU positive cell from experiment as described in (**c**). Data represent mean ± SEM derived from at least 200 EdU positive cells (*n* = 2 independent experiments); *p* values (*p* < 0.0001, *p* < 0.0001, *p* < 0.0001, *p* < 0.0001) are indicated. **e** Schematic showing experimental strategy of native IdU assay. Scatter plot showing quantification of nascent ssDNA (IdU foci number) in γH2AX positive cells. Data represent mean ± SEM derived from at least 180 cells (*n* = 2 independent experiments).; *p* values (*p* < 0.0001, *p* < 0.0001, *p* < 0.0001, *p* < 0.0001) are indicated. **f** Scatter plot showing BRCA1 foci in Wild type (WT) or S404A/S406A ABRAXAS expressing HT-29 cells treated with CPT (1 µM) +/- ATMi (250 nM) for 1 h. Experiments were repeated four times with similar results. Data represent mean ± SEM derived from at least 150 cells (*n* = 2 independent experiments); *p* values (*p* = 0.0014, *p* < 0.0001, *p* = 0.0006) are indicated. **g** Scatter plot showing quantification of RPA foci in the indicated genoty*p*es of HT-29 cells treated as in (**f**). Experiments were repeated three times with similar results. Data represent mean ± SEM derived from at least 110 cells (*n* = 2 independent experiments); *p* values (*p* < 0.0001, *p* = 0.0096, *p* = 0.0296, *p* = 0.7884, *p* = 0.0415) are indicated. **h** Clonogenic survival assay showing CPT+ATMi resistance in HT-29 cells expressing S404A/S406A ABRAXAS. Data represent mean ± SD from *n* = 3 independent experiments. In (**d**, **e**, **f**, **g**), *p* values were calculated with unpaired two-tailed t test. Source data are provided as a Source Data file.

BRCA1-A loss was reported to confer resistance to fork damaging drugs in ATM-deficient cancer cells^39^. Consistent with the previous results, we observed hypersensitivity of HEK293T cells to nanomolar doses of Topoisomerase 1 inhibitor (Top1i) camptothecin (CPT) when combined with AZD0156^39^, a potent and selective pharmacological ATM kinase inhibitor (ATMi)^42,43^ (Fig. 1a and Supplementary Fig. 1a, b). ABRAXAS knockout (KO) substantially reduced hypersensitivity to combined CPT and ATMi but did not affect responses to CPT alone. Similar results were obtained in ATM and ABRAXAS double KO HT-29 cells (Fig. 1b and Supplementary Fig. 1c, d). ABRAXAS knockout also produced PARPi resistance specifically in the context of ATMi (Supplementary Fig. 1e).

Restored end resection confers drug resistance in HR-compromised cancer cells^44,45^. Top1 inhibitors such as CPT generate distinct replication fork intermediates including one-ended double-strand breaks (DSBs) and reversed replication forks. While a high micromolar (µM) dose of CPT induces both one ended DSBs and reversed forks, low nanomolar CPT treatment causes fork reversal without generating DSBs^46,47^. Reversed fork structures resemble one-ended DSBs and are susceptible to nucleolytic processing by MRE11, EXO1 or DNA2 nucleases^48–51^. Therefore, genetic suppression of hypersensitivity to low nanomolar CPT treatment by BRCA1-A loss in ATM-deficient cells (Fig. 1a, b) raises the possibility of reversed forks as resection substrates in BRCA1-A deficient cells. We performed RPA immunofluorescence as a read out of single strand DNA generated from end resection in control or ABRAXAS KO HT-29 cells exposed to a low dose (50 nM) of CPT in combination with ATMi (Fig. 1c, d). Compared to untreated or only ATMi treated conditions, CPT treatment significantly induced RPA signal in both control and ABRAXAS KO cells. ATMi markedly reduced RPA signal, which was restored in ABRAXAS KO cells, suggesting BRCA1-A restricts end resection upon ATM inhibition. The increased RPA signal was evident in cells that stained positive for EdU incorporation, indicating that elevated resection in A-complex null cells occurs in S-phase (Fig. 1c, d). We also observed elevated resection upon BRCA1-A loss in ATM inhibited cells in response to a high dose (1 µM) of CPT that induces both DSBs and fork reversal (Supplementary Fig. 2a, b).

We performed native IdU assay under non-denaturing conditions to detect nascent ssDNA specifically at reversed fork structures^52–54^ (Fig. 1e and Supplementary Fig. 2c). ATMi suppressed nascent ssDNA in cells exposed to a low dose of CPT, while ABRAXAS KO significantly restored it under these conditions. We observed similar results in ATM KO cells with a significant restoration of ssDNA in ATM and ABRAXAS double KO cells (Fig. 1e and Supplementary Fig. 2c). ATMi did not further exacerbate ssDNA suppression in ATM KO cells, thus attesting to the specificity of the ATM inhibitor. In addition to a high CPT dose (2 µM), BRCA1-A loss significantly restored ssDNA in response to as low as 8 nM dose of CPT that does not generally cause DSBs^46,47^(Supplementary Fig. 2d**)**. These results are suggestive that BRCA1-A loss restores end resection at reversed fork intermediates in ATM-deficient cells.

### BRCA1 interaction with the A-complex suppresses fork resection and viability in ATM-deficient cells

The BRCA1-A complex harbors DNA damage recognition moieties contained within RAP80 and BRCA1-BARD1. We therefore examined if both elements were required for its damage localization in ATMi treated cells. Compared to CPT treatment alone, we observed significantly increased RAP80 foci following ATMi (Supplementary Fig. 3a, b) which is consistent with a previous report^43^. Similar results were obtained for ABRAXAS and BRCA1 recruitment (Supplementary Fig. 3c–f), suggesting ATMi triggers BRCA1-A engagement at damaged forks. However, BRCA1 damage localization was significantly impaired in ABRAXAS KO cells (Supplementary Fig. 3e, f). N-terminal S404 and S406 residues of ABRAXAS (hereafter S404A/S406A) interact with BRCA1 BRCT in a phosphorylation dependent manner^55^. Compared to wild type (WT) cells, ATMi-induced BRCA1 damage localization was significantly impaired in cells complemented with S404A/S406A mutant (Fig. 1f, and Supplementary Fig. 4a, b). BRCA1 foci formation was also considerably reduced in S404A/S406A mutant cells treated with CPT alone, indicating A-complex dependence of BRCA1 damage recognition in ATM-proficient cells as reported previously^37,55^. Although to a lesser extent than in ABRAXAS wild-type cells, ATMi led to a moderate increase in BRCA1 damage localization even in ABRAXAS S404A/S406A mutant cells (Fig. 1f). This likely reflects a contribution of BARD1 interaction with H2AK15Ub and H4K20Me0 chromatin marks independent of A-complex association^29,30^. Accordingly, a concomitant decrease in ABRAXAS foci formation also occurred in S404A/S406A mutant cells (Supplementary Fig. 4c, d). This indicates that damaged fork localization relies on coordinated A-complex and BRCA1-BARD1 recognition of modified nucleosomes^17,18,20,28–30,33^.

We further evaluated the effect of complete BRCA1-A assembly on end resection in ATM inhibited cells. While WT ABRAXAS complementation suppressed RPA foci in ABRAXAS KO cells, the S404A/S406A mutant did not (Fig. 1g and Supplementary Fig. 4e), thereby mimicking A-complex null effects. Native IdU assay further revealed significantly increased nascent ssDNA in S404A/S406A mutant cells as compared to WT when exposed to ATMi (Supplementary Fig. 4f). Consistent with these results, HT-29 cells expressing S404A/S406A mutant showed CPT+ATMi resistance to a similar extent to ABRAXAS KO (Fig. 1h and Supplementary Fig. 5a). Concordant results were obtained in ABRAXAS KO HEK293T cells complemented with ABRAXAS S404A/S406A (Supplementary Fig. 5b, c). We conclude that a fully assembled BRCA1-A complex is required to limit end resection and viability in ATM-deficient cells.

### BRCA1-A regulates chromatin and nuclease accessibility at damaged forks

**Fig. 2:**
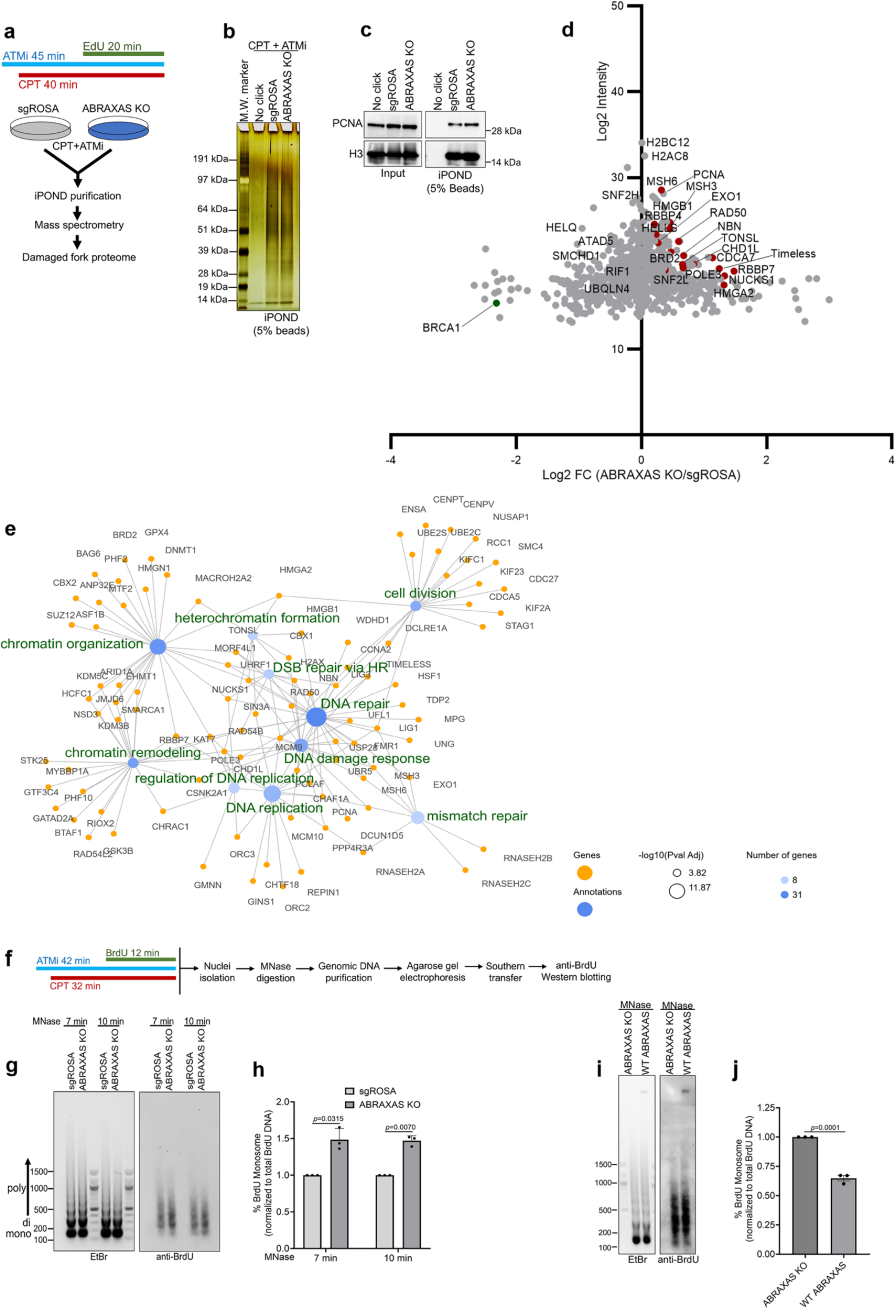
BRCA1-A loss promotes chromatin accessibility and nuclease recruitment at damaged forks in ATM-inhibited cells. **a** Experimental scheme of the iPOND-MS in control (sgROSA) and ABRAXAS KO HEK293T cells. iPOND purified samples (5% beads) were analyzed by silver staining (**b**) and Western blotting (**c**). **d** Scatter plot showing quantification of the iPOND-MS data (*n* = 5). X axis shows log2 fold change (FC) of proteins in ABRAXAS KO cells over sgROSA. Y axis shows average log2 intensity of proteins across sgROSA and ABRAXAS KO cells. Proteins shown in red include nucleases, HR repair factors and chromatin remodelers. **e** Gene-annotation clusters network showing top 10 significantly enriched GO terms for the upregulated proteins in ABRAXAS KO cells. **f** Experimental scheme of BrdU-coupled chromatin accessibility assay in control (sgROSA) and ABRAXAS KO HEK293T cells treated with CPT (1 µM) +/- ATMi (250 nM). **g** Representative Ethidium bromide (EtBr)-stained agarose gel (left) and Southern-Western blots (right) of MNase digested BrdU-labeled nucleosome fractions derived from cells as described in (**f**). MNase digestion time (min) is indicated. **h** Bar graph showing relative percentage of BrdU-labeled mononucleosome fractions normalized to total amount of BrdU-labeled DNA per lane. Data represent mean ± SEM of *n* = 3 independent experiments; *p* values (*p* = 0.0315, *p* = 0.0070) are indicated. **i**, **j** Representative Ethidium bromide (EtBr)-stained agarose gel (left) and Southern-Western blots (right) of MNase digested BrdU-labeled nucleosome fractions derived from ABRAXAS KO or ABRAXAS complemented HEK293T cells treated as described in (**f**). Nuclei were subjected to MNase digestion for 10 min followed by Southern-Western blotting (**i**). Bar graph showing decreased BrdU-labeled mononucleosome fractions upon ABRAXAS complementation (**j**). Data represent mean ± SEM of *n* = 3 independent experiments; *p* values (*p* = 0.0001) are indicated. In **h**, **j**, *p* values were calculated with unpaired two-tailed t test. In **g**, **i** DNA ladder is shown. In **b**, **c**, molecular weight markers are shown. In **b**, **c**, **g**, **i** experiments were performed three times with similar results. Source data are provided as a Source Data file.

To identify mechanisms underlying BRCA1-A mediated suppression of end resection, we characterized damaged fork proteome in ABRAXAS KO cells using iPOND coupled with mass spectrometry (iPOND-MS). WT or ABRAXAS KO HEK293T cells were exposed to combined CPT and ATMi followed by iPOND purification and mass spectrometry analysis (Fig. 2a, b). Western blot revealed specific enrichment of PCNA in iPOND samples as compared to no-click control (Fig. 2c), thereby validating the specificity of the iPOND purification. BRCA1 was depleted in ABRAXAS KO cells, suggesting the A-complex targets BRCA1 to damaged forks (Fig. 2d). MRE11-RAD50-NBS1 (MRN) complex components RAD50 and NBN along with EXO1 nuclease were enriched at damaged forks in ABRAXAS KO cells. Additionally, HR proteins including TIMELESS and TONSL, and several chromatin remodelers were elevated at damaged forks in ABRAXAS KO cells. Gene Ontology (GO) analysis further showed significant enrichment of the DDR response, chromatin organization and remodeling pathways upon BRCA1-A loss (Fig. 2e). These results are consistent with a hypothesis that BRCA1-A restricts nuclease access to damaged forks by regulating chromatin accessibility in ATM deficient cells.

Chromatin remodeling allows physical access of nucleases and repair factors to damaged DNA templates^56–61^. ATM kinase activity is critical for chromatin relaxation following DSB induction^62,63^. In response to replication stress, nascent chromatin around replications forks undergoes dynamic changes via nucleosome modifications and remodeling, thereby facilitating fork processing and other transactions^61,64^. We hypothesized that BRCA1-A regulates chromatin accessibility at damaged forks following ATMi given the increased abundance of resection nucleases along with chromatin remodelers in ABRAXAS null cells (Fig. 2d, e). To determine accessibility specifically at damaged replication forks, we labeled nascent chromatin with a short pulse of BrdU followed by partial MNase digestion and subsequent detection of BrdU labeled chromatin fragments by Southern-Western blotting (Fig. 2f). Compared to control cells, we observed significantly increased BrdU-labeled mono nucleosome fractions in ABRAXAS KO cells, suggesting increased chromatin accessibility upon BRCA1-A loss (Fig. 2g, h). Complementation of ABRAXAS KO cells with WT ABRAXAS reduced chromatin accessibility at CPT-damaged forks under ATMi conditions (Fig. 2i, j), confirming the specificity of these observations.

### Increased end resection upon BRCA1-A loss confers drug resistance in ATM-deficient cells

**Fig. 3:**
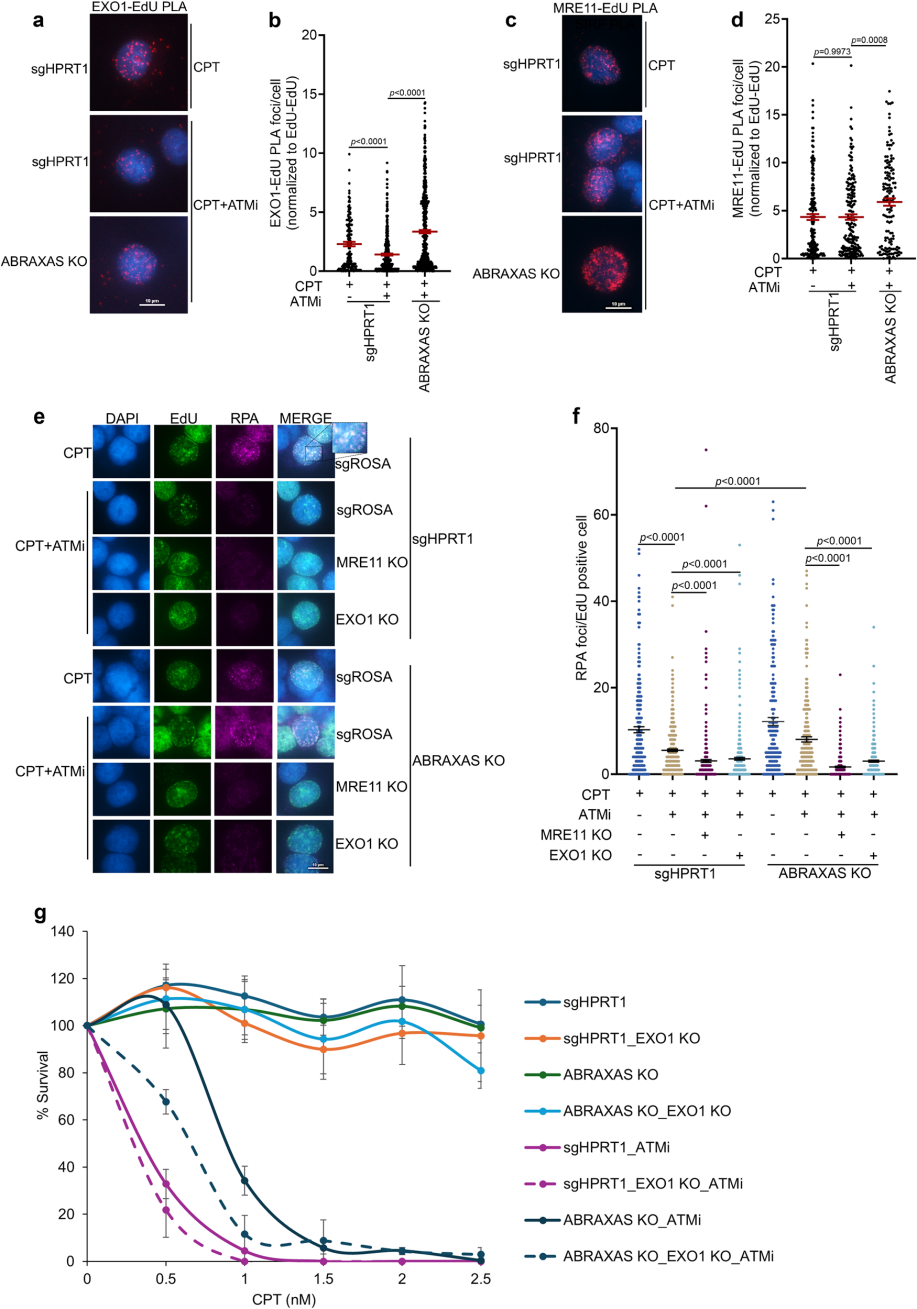
EXO1 and MRE11-mediated end resection confers drug resistance in BRCA1-A deficient cells. **a**, **b** Representative images of EXO1-EdU PLA SIRF in control (sgHPRT1) and ABRAXAS KO HT-29 cells treated with CPT (1 µM) +/- ATMi (250 nM) for 40 min. Cells were optionally pretreated with ATMi for 10 min and labeled with EdU during the last 20 min of CPT treatment. ATMi was present throughout the experiment (**a**). Scatter plot showing quantification of EXO1-EdU PLA foci normalized to average EdU-EdU signal in individual experimental conditions (**b**). Data represent mean ± SEM derived from at least 139 cells (*n* = 2 independent experiments); *p* values (*p* < 0.0001, *p* < 0.0001) are indicated. **c**, **d**, Representative images of MRE11-EdU PLA SIRF in cells described and treated as in (**a**). **c** Scatter plot showing quantification of the data from experiment described in (**c**). **d** Data represent mean ± SEM derived from at least 140 cells (*n* = 2 independent experiments); *p* values (*p* = 0.9973, *p* = 0.0008) are indicated. **e** Representative images of RPA immunofluorescence in control (sgHPRT1) and ABRAXAS KO HT-29 cells infected with sgROSA or knocked out of MRE11 or EXO1 following treatment with CPT (50 nM) +/- ATMi (250 nM) for 1 h. Cells were concomitantly labeled with EdU (10 µM) to identify S-phase cells. ATMi was added 10 min before CPT treatment. An inset shows colocalization of RPA with EdU spot. **f** Scatter plot showing quantification of the RPA foci in EdU positive cells as described in (**e**). Data represent mean ± SEM derived from at least 240 cells (*n* = 2 independent experiments); *p* values (*p* < 0.0001, *p* < 0.0001, *p* < 0.0001, *p* < 0.0001, *p* < 0.0001, *p* < 0.0001) are indicated. **g** Relative colony survival (%) of the indicated genotypes of HT-29 cells treated as indicated with CPT and ATMi. Data represent mean ± SD derived from *n* = 3 independent experiments. In **a**, **c**, **e**, images were acquired with 100X magnification. Scale bar is shown. In **b**, **d**, **f** *p* values were calculated with unpaired two-tailed t test. Source data are provided as a Source Data file.

Controlled nucleolytic processing of damaged forks is critical for HR-dependent fork recovery and cell survival^48,49,65^. Because iPOND-MS revealed fork enrichment of EXO1 and MRN nucleases upon BRCA1-A loss, we investigated whether these nucleases promote end resection in this setting. We validated our iPOND-MS results using single molecule PLA-SIRF in cells treated with CPT alone or in combination with ATMi. We observed a significant decrease in the EXO1-EdU PLA foci upon ATMi that was restored following ABRAXAS KO, consistent with our iPOND-MS results (Fig. 3a, b). We also detected significantly increased fork association of MRE11 nuclease upon BRCA1-A loss in ATMi-treated cells (Fig. 3c, d). These results suggest BRCA1-A loss generates replication fork intermediates that engage resection nucleases. We subsequently knocked out MRE11 and EXO1 and analyzed end resection by RPA immunofluorescence in ABRAXAS KO cells treated with a low dose (50 nM) of CPT in combination with ATMi (Fig. 3e, f, Supplementary Fig. 6a, b). MRE11 and EXO1 KO significantly reduced RPA foci in ABRAXAS KO cells exposed to combined CPT and ATMi (Fig. 3e, f), indicating elevated end resection upon BRCA1-A loss in ATM-deficient cells is dependent on MRE11 and EXO1 nucleases. Consistent with the end resection data, EXO1 KO partially resensitized ABRAXAS KO cells to combined CPT and ATMi treatment (Fig. 3g and Supplementary Fig. 6c). These results suggest that Top1i + ATMi resistance upon BRCA1-A loss occurs via MRE11 and EXO1-mediated end resection.

### ATM inhibition promotes SUMO and ubiquitin dependent BRCA1-A localization to damaged forks

**Fig. 4:**
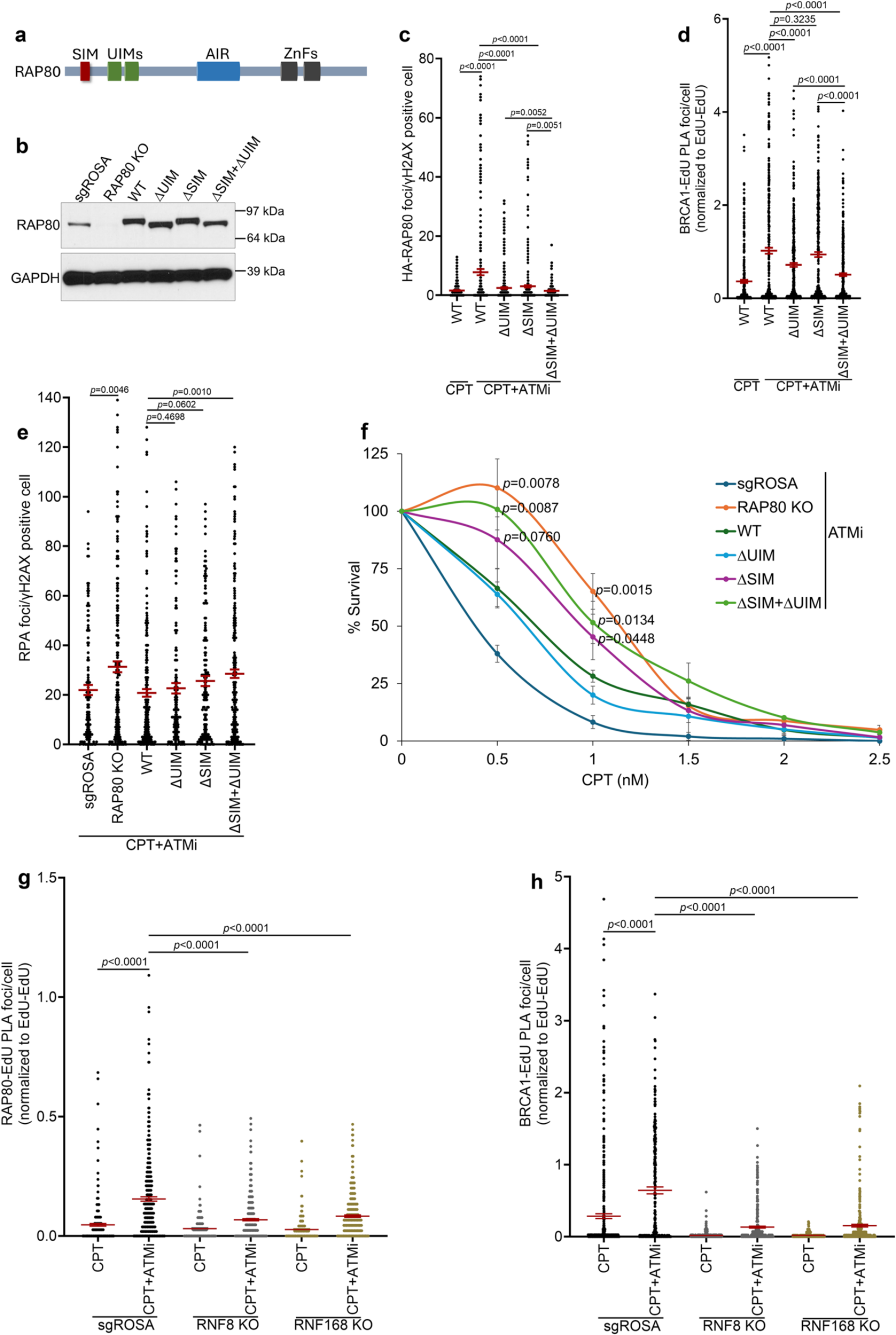
Combined SUMO-ubiquitin recognition determines BRCA1-A function at damaged forks. **a** Schematic showing structural domains of RAP80. SIM, SUMO interaction motif; UIMs, Ubiquitin interaction motifs; AIR, ABRAXAS interacting region; ZnFs, Zinc finger domains. **b** Western blots showing RAP80 protein levels in control (sgROSA) and RAP80 KO HT-29 cells complemented with WT or indicated RAP80 mutants. GAPDH serves as loading control. Molecular weight markers are shown. Western blotting was repeated three times with similar results. **c** Quantification of HA-RAP80 foci in the indicated genotypes treated with CPT (1 µM) +/- ATMi (250 nM) for 1 h. *p* values (*p* < 0.0001, *p* < 0.0001, *p* < 0.0001, *p* = 0.0052, *p* = 0.0051) were determined from at least 230 cells (*n* = 2 independent experiments). **d** Quantification of BRCA1-EdU PLA foci in cells as described in (**b**). Cells were treated with CPT (50 nM) +/- ATMi (250 nM) for 40 min. *p* values (*p* < 0.0001, *p* < 0.0001, *p* = 0.3235, *p* < 0.0001, *p* < 0.0001, *p* < 0.0001) were determined from at least 329 cells (*n* = 2 independent experiments). **e** Quantification of RPA immunofluorescence in the indicated genotypes treated as in (**c**). *p* values (*p* = 0.0046, *p* = 0.4698, *p* = 0.0602, *p* = 0.0010) were determined from at least 125 cells (*n* = 2 independent ex*p*eriments). **f** Relative colony survival (%) of cells as described in (**b**). Cells were treated with 100 nM ATMi and the indicated doses of CPT. *p* values (*p* = 0.0078, *p* = 0.0087, *p* = 0.0760, *p* = 0.0015, *p* = 0.0134, *p* = 0.0448) were determined for RAP80 KO (EV), ∆SIM or ∆SIM + ∆UIM over WT at CPT concentrations of 0.5 and 1 nM; unpaired two-tailed t test. Data represents mean ± SD from *n* = 3 biological experiments. **g** Quantification of RAP80-EdU PLA foci in the indicated genotypes of HT-29 cells treated with 50 nM CPT +/- ATMi (250 nM) for 1 h. *p* values (*p* < 0.0001, *p* < 0.0001, *p* < 0.0001) were determined from at least 279 cells (*n* = 2 independent experiments). **h** Quantification of BRCA1-EdU PLA foci in cells treated as in (**g**). *p* values (*p* < 0.0001, *p* < 0.0001, *p* < 0.0001) were determined from at least 239 cells (*n* = 2 independent experiments). In (**c**, **d**, **e**, **g**, **h**), data represent mean ± SEM; *p* values were calculated with unpaired two-tailed t test. Source data are provided as a Source Data file.

Double strand break (DSB) recognition by the A- complex is mediated by RAP80 binding to K63-linked polyubiquitin chains via its tandem ubiquitin-interacting motifs (UIMs)^18–20^. Additionally, RAP80 harbors a small SUMO interaction motif (SIM) that contributes to RAP80 and BRCA1 damage localization^28^ (Fig. 4a). In vitro binding experiments demonstrated that recombinant RAP80 has an 80-fold higher binding affinity for hybrid SUMO-ubiquitin chains compared to monomeric SUMO-2 or diUb alone^28^. We therefore assessed how RAP80 ubiquitin and SUMO recognition contributes to BRCA1-A damage accumulation during ATM kinase deficiency. We complemented RAP80 KO cells with RAP80 WT or deletion mutants lacking UIMs, SIM, or both SIM and UIM domains (Fig. 4b) and tested RAP80 damage localization. WT cells showed increased RAP80 damage accumulation upon combined CPT and ATMi treatment (Fig. 4c and Supplementary Fig. 7a). In contrast, individual deletion of UIM or SIM showed decreased RAP80 foci formation which was even more pronounced upon combined UIM and SIM loss (Fig. 4c and Supplementary Fig. 7a). In accordance, inhibition of SUMOylation by the SUMO E1 inhibitor ML-792 partially reduced RAP80-EdU PLA signal in cells exposed to a low dose of CPT (50 nM) in combination with ATMi (Supplementary Fig. 7b, c). While RAP80 UIM deletion reduced BRCA1 fork binding, we did not see a statistically significant decrease in BRCA1-EdU signal upon RAP80 SIM deletion in ATMi-treated cells (Fig. 4d). However, combined SIM and UIM deletion significantly suppressed BRCA1 damage localization as compared to deletion of individual domain alone (Fig. 4d). These results indicate that while RAP80 UIM is adequate to recruit BRCA1 at forks, combined SUMO and ubiquitin recognition by RAP80 is required for stable association of BRCA1 with damaged forks. Consistent with these results, SIRF-PLA experiments revealed a significant increase in SUMO 2/3 and ubiquitin fork abundance upon ATMi (Supplementary Fig. 7d, e).

To determine the impact of ubiquitin or SUMO recognition on fork resection, we examined RPA foci formation in RAP80 WT or mutant cells exposed to CPT plus ATMi. While WT RAP80 complementation suppressed end resection in RAP80 KO cells, concurrent deletion of SIM and UIM domains significantly rescued RPA signal (Fig. 4e and Supplementary Fig. 8a). This reflects requirements for both SUMO and ubiquitination at damaged forks upon ATMi. We further investigated whether SIM and UIM deletion affects CPT sensitivity in ATM-inhibited cells. RAP80 KO showed CPT resistance when combined with ATMi, whereas complementation with WT RAP80 reinstated drug sensitivity (Fig. 4f and Supplementary Fig. 8b). In agreement with the end resection data, we observed an overall significant increase in drug resistance upon combined loss of SIM and UIM, but not upon deletion of individual domain alone. Together, these results indicate that combined SUMO and ubiquitin recognition by RAP80 is required for maximal suppression of resection and drug hypersensitivity in ATM-deficient cells.

Given their central role in DSB ubiquitin signaling^66^, we tested if E3 ubiquitin ligases RNF8 and RNF168 are involved in fork ubiquitylation. Fork ubiquitylation was significantly diminished upon RNF8 or RNF168 KO in ATMi-treated cells (Supplementary Fig. 9a, b), accompanied by reduced fork binding of RAP80 (Fig. 4g) and BRCA1 (Fig. 4h). These results were further supported by increased fork binding of RNF8 (Supplementary Fig. 9c) and RNF168 (Supplementary Fig. 9d) upon ATMi, consistent with previous findings^43^. These results suggest that RNF8 and RNF168-dependent increased fork ubiquitination contributes to BRCA1-A recruitment to damaged forks upon ATM inhibition. ATM phosphorylates H2AX and MDC1 to promote RNF8 and RNF168 recruitment to damaged chromatin^66^. We found that H2AX or MDC1 KO reduced RNF8-/RNF168-EdU PLA signal (Supplementary Fig. 9c, d). However, RNF8 or RNF168 fork abundance was found to be increased to some extent upon ATMi even in H2AX or MDC1 KO cells (Supplementary Fig. 9c, d). This points to an additional H2AX/MDC1 independent mechanism of RNF8/168 fork recruitment under ATM-deficient conditions.

### BRCA1-A prevents fork reversal in ATM-deficient cells

Top1 poisons induce high rates of replication fork reversal, a RAD51-dependent protective mechanism that limits formation of DSBs and facilitates repair or bypass of DNA lesions^46,47,67^. Fork reversal is catalyzed by ATP-dependent DNA translocases including SMARCAL1, ZRANB3, HLTF and FBH1^68^. Immunofluorescence imaging results of RPA (Fig. 1c, d) and native IdU (Fig. 1e, and Supplementary Fig. 2c, d) using low nanomolar dose of CPT are consistent with increased end-resection at reversed fork substrates in BRCA1-A deficient cells. We extended this analysis in cells treated with hydroxyurea (HU), which causes reversed fork degradation in BRCA1 or BRCA2 deficient cells^51,69,70^. BRCA1 depletion led to fork degradation, while ABRAXAS KO did not affect stability of HU-stalled forks (Supplementary Fig. 10a). We observed a moderate but significant decrease in replication tract length in ABRAXAS KO cells when exposed to ATMi (Supplementary Fig. 10a). Similar results were observed in native IdU immunofluorescence experiments (Supplementary Fig. 10b), suggesting controlled resection of HU-induced reversed forks occurs when BRCA1-A loss is concurrent with ATM-deficiency.

**Fig. 5:**
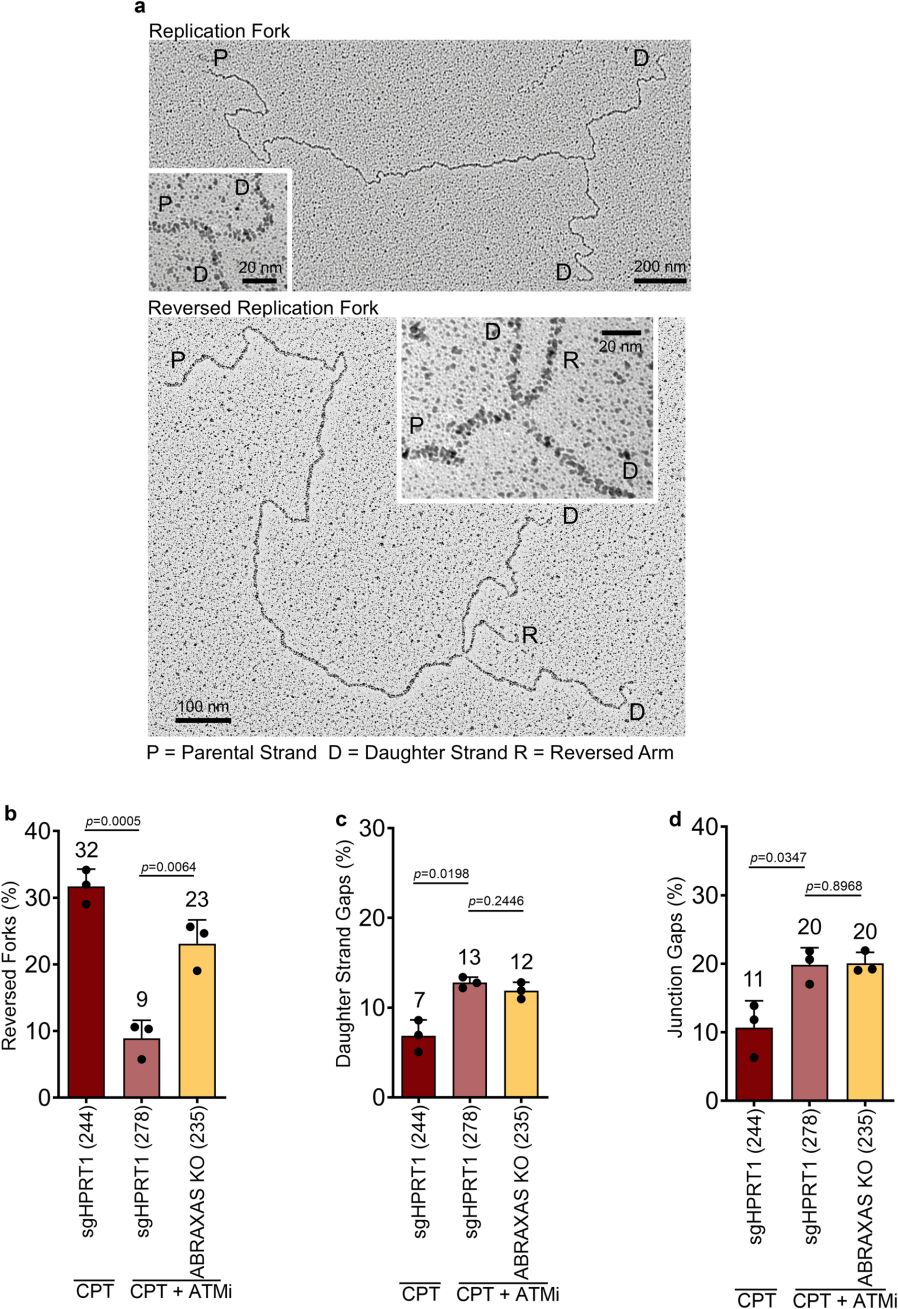
BRCA1-A complex prevents fork reversal upon ATM inhibition. **a** Top panel: Electron micrograph showing a typical replication fork with a three-way junction. Bottom panel: Electron micrograph of a typical reversed replication fork with a four-way junction. Representative images are from psoralene-crosslinked genomic DNA isolated from ABRAXAS KO HT-29 cells treated with CPT+ATMi. P, parental strands; D, daughter strands; R, reversed arm. Cells were treated with 50 nM CPT for 1 h. ATMi (250 nM) was added optionally 10 min prior to CPT treatment and present throughout the experiment. Scale bar is shown. Experiment was repeated three times with similar results. **b** Bar graph showing frequency of fork reversal in control (sgHPRT1) cells treated with CPT +/-ATMi or ABRAXAS KO cells treated with CPT+ATMi. Fractions of reversed forks (number above bars) as a percentage of total number of replication intermediates analyzed (bottom, within parentheses) are shown. Bar graphs showing fractions of post-replicative daughter strand gaps (**c**) and junction gaps (**d**) detected under experimental conditions as described in (**a**). In **b**–**d**, data represent mean ± SD from *n* = 3 independent biological experiments; *p* values (**b**; *p* = 0.0005, *p* = 0.0064, **c**; *p* = 0.0198, *p* = 0.2446, **d**; *p* = 0.0347, *p* = 0.8968) are indicated; unpaired two-tailed t test. Source data are provided as a Source Data file.

We performed electron microscopy (EM) to quantitatively determine the abundance of reversed fork structures following a 50 nM dose of CPT treatment that is established to cause fork reversal without generating DSBs^46,47,71,72^ (Fig. 5a, b). Consistent with published results^46,47^, we detected an average of 32% reversed fork structures upon CPT treatment across three independent biological experiments (Fig. 5b). We observed a > 3-fold decrease in fork reversal events in ATMi + CPT treated cells, revealing a critical role of ATM kinase in promoting damaged fork reversal. Notably, ABRAXAS KO restored fork reversal by 2.5-fold in ATMi treated cells (Fig. 5b). The frequency of post-replicative ssDNA gaps (Fig. 5c) and fork junction gaps (Fig. 5d) were increased upon ATMi. This may reflect repriming and accumulation of uncoupled forks with extended parental ssDNA at fork junctions, respectively due to impaired fork reversal in ATM-inhibited cells. However, EM analysis did not reveal significant changes in the frequency of gap formation in ABRAXAS KO cells (Fig. 5c, d), indicating the BRCA1-A complex does not affect gap sizes that are detectable by EM.

### Reversed forks are resection substrates in cells deficient for ATM and BRCA1-A

**Fig. 6:**
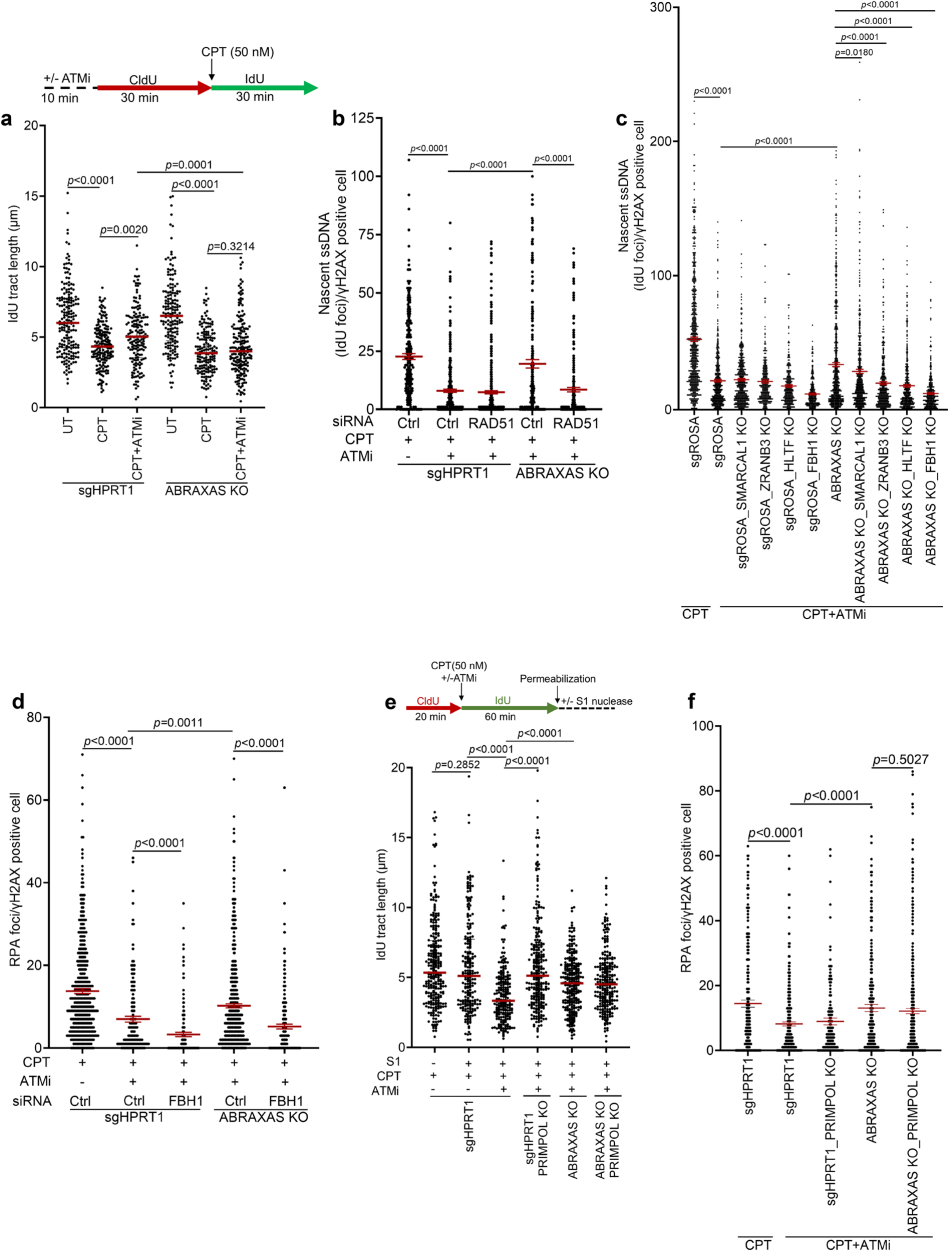
BRCA1-A blocks resection at reversed forks in ATM-inhibited cells. **a** Schematic showing fork slowing assay in control (sgHPRT1) or ABRAXAS KO HT-29 cells treated with CPT (50 nM) ± ATMi (250 nM). Scatter plot represents IdU tract length in individual genotypes treated as indicated. Data represent median IdU tract length with interquartile range derived from at least 150 DNA fibers (*n* = 2 independent experiments); *p* values (*p* < 0.0001, *p* = 0.0020, *p* = 0.0001, *p* < 0.0001, *p* = 0.3214) are indicated; two-tailed Mann–Whitney test. **b** Quantification of nascent ssDNA in the indicated genotypes of HT-29 cells upon RAD51 depletion. Cells were treated with CPT (50 nM) ± ATMi (250 nM) for 1 h. Data represent mean ± SEM derived from at least 230 cells (*n* = 2 independent experiments); *p* values (*p* < 0.0001, *p* < 0.0001, *p* < 0.0001) are indicated. **c** Quantification of nascent ssDNA in the indicated genotypes of U2OS cells treated as in (**b**). Data represent mean ± SEM derived from at least 272 cells (*n* = 2 independent experiments); *p* values (*p* < 0.0001, *p* < 0.0001, *p* = 0.0180, *p* < 0.0001, *p* < 0.0001, *p* < 0.0001) are indicated. **d** Quantification of RPA foci in control (sgHPRT1) or ABRAXAS KO HT-29 cells upon siRNA mediated depletion of FBH1. Seventy-two h post-transfection, cells were treated as in (**b**). Data represent mean ± SEM derived from at least 147 cells (*n* = 2 independent experiments); *p* values (*p* < 0.0001, *p* < 0.0001, *p* = 0.0011, *p* < 0.0001) are indicated. **e** Schematic showing S1 nuclease assay with CPT (50 nM) ± ATMi (250 nM). Scatter plot showing quantification of IdU tract length in the indicated HT-29 genotypes. Data represent median IdU tract length with interquartile range derived from at least 222 DNA fibers (*n* = 2 independent experiments); *p* values (*p* = 0.2852, *p* < 0.0001, *p* < 0.0001, *p* < 0.0001) are indicated; two-tailed Mann–Whitney test. **f** Quantification of RPA immunofluorescence in the indicated genoty*p*es of HT-29 cells treated as in (**b**). Experiments were performed three times with similar results. Data represent mean ± SEM derived from at least 129 cells (*n* = 2 independent experiments); *p* values (*p* < 0.0001, *p* < 0.0001, *p* = 0.5027) are indicated. In **b**, **c**, **d**, **f**, *p* values were calculated with unpaired two-tailed t test. Source data are provided as a Source Data file.

Because CPT-induced fork reversal slows down replication, we assessed fork speed using DNA fiber analysis as a surrogate measure of fork reversal^47,67,71,73^. ATMi partially rescued replication tract length in CPT-treated cells, consistent with compromised fork reversal under this condition (Fig. 6a). However, ATMi did not significantly increase fork speed in ABRAXAS KO cells, thus reflecting restored fork reversal in these cells (Fig. 6a), in agreement with EM data (Fig. 5).

These results imply that BRCA1-A loss generates substrates for nucleolytic resection by restoring fork reversal in ATM-inhibited cells. To test this hypothesis, we treated ABRAXAS KO cells with 50 nM CPT ± ATMi and examined nascent ssDNA by native IdU assay after depletion of RAD51, which is reported to be essential for replication fork reversal^47,67^. RAD51 depletion diminished nascent ssDNA in ABRAXAS KO cells exposed to ATMi (Fig. 6b and Supplementary Fig. 11a, b), consistent with fork reversal being the source of resection substrates in BRCA1-A deficient cells. To further test the importance of fork reversal for resection, we determined ssDNA levels upon knockout of fork reversal enzymes SMARCAL1, ZRANB3, HLTF or FBH1 (Fig. 6c and Supplementary Fig. 11c). SMARCAL1 KO did not show an appreciable change in IdU labeled ssDNA. In contrast, KO of HLTF, ZRANB3 or FBH1 suppressed ssDNA in ABRAXAS KO cells exposed to CPT+ATMi, with FBH1 loss showing a dramatic reduction in native ssDNA generation (Fig. 6c and Supplementary Fig. 11c). Consistent with these results, FBH1 depletion suppressed enhanced RPA signal in ABRAXAS KO cells, confirming reversed forks are resection substrates in these cells (Fig. 6d and Supplementary Fig. 11d).

Post-replicative single-stranded DNA gaps also arise following replication stalling via PRIMPOL mediated repriming^73–75^, representing a second possibility for DNA substrates that could serve as resection substrates in the context of concomitant ATM kinase and BRCA1-A deficiency. PRIMPOL-dependent ssDNA gaps were formed in ATMi-treated cells as revealed by reduced fiber tract length when exposed to S1 nuclease (Fig. 6e). We observed a significant rescue in DNA fiber tract length upon ABRAXAS KO, suggesting BRCA1-A loss suppresses ssDNA gaps in ATM-inhibited cells. This seemingly contrasts with EM analysis that showed unaltered ssDNA gaps in BRCA1-A deficient cells (Fig. 5c, d). However. this discrepancy is likely attributed to the limitation of the EM approach to detect gaps shorter than 40–60 nucleotides^76^. Nucleolytic processing extends ssDNA gaps resulting in RPA coated ssDNA on parental DNA strand^77,78^. However, PRIMPOL KO did not affect RPA foci in ABRAXAS KO cells treated with 50 nM CPT ± ATMi, suggesting resection at gaps is not responsible for the elevated ssDNA (Fig. 6f and Supplementary Fig. 11e, f).

### BRCA1-A loss restores fork restart and chromosome stability in ATM-deficient cells

**Fig. 7:**
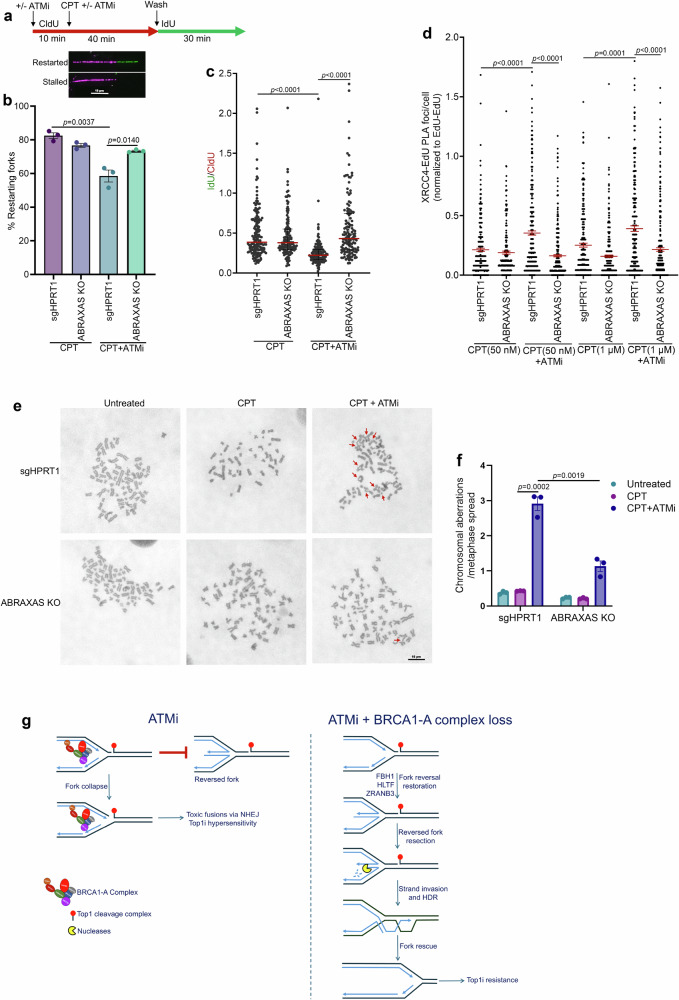
BRCA1-A loss promotes damaged fork recovery and suppresses chromosomal aberrations in ATM-inhibited cells. **a** Schematic showing fork restart assay with CPT (50 nM) +/- ATMi (250 nM). Representative fiber images of a restarted and a stalled fork are shown. **b** Bar graph showing fractions of restarted forks (%) in the indicated genotypes of HT-29 cells treated as indicated. Data represent mean ± SEM derived from more than 300 fibers analyzed over *n* = 3 independent experiments; *p* values (*p* = 0.0037, *p* = 0.0140) are indicated. **c** IdU/CldU ratios determined from the experiment described in (**a**, **b**). *p* values (*p* < 0.0001, *p* < 0.0001) were determined from at least 135 DNA fibers (*n* = 3 independent experiments); two-tailed Mann–Whitney test. Data represent median IdU/CldU with interquartile range. **d** Quantification of XRCC4-EdU PLA foci in control (sgHPRT1) and ABRAXAS KO HT-29 cells treated as indicated. Data represent mean ± SEM from at least 271 cells (*n* = 3 independent experiments); *p* values (*p* < 0.0001, *p* < 0.0001, *p* = 0.0001, *p* < 0.0001) are indicated. **e** Representative images of metaphase chromosomes prepared from control (sgHPRT1) and ABRAXAS KO HT-29 cells treated as indicated. Red arrows indicate chromosomal aberrations. Images were acquired with 100X magnification. **f** Quantification of the data from (**e**). Data represent mean ± SEM of at least 60 metaphase spreads from *n* = 3 independent experiments.; *p* values (*p* = 0.0002, *p* = 0.0019) are indicated. **g** Model illustrating how BRCA1-A complex loss causes Top1i resistance in ATM deficient cells. ATM inhibition (ATMi) promotes BRCA1-A complex engagement at damaged forks, resulting in inhibition of fork reversal and eventual replication collapse. Illegitimate NHEJ at collapsed forks causes toxic chromosome fusions leading to Top1i hypersensitivity (left). BRCA1-A complex loss restores fork reversal catalyzed by FBH1, HLTF, and ZRANB3, thereby generating resection substrates for MRE11 and EXO1 nucleases. Controlled resection of a reversed fork generates 3´-ssDNA that invades parental duplex ahead of the fork via HDR mechanism^49,68,79^. This leads to fork rescue, resulting in Top1i resistance in ATM inhibited cells (right). In **b**, **d**, **f**, *p* values were calculated with unpaired two-tailed t test. In **a**, **e**, scale bars are shown. Source data are provided as a Source Data file.

Fork reversal allows damage fork recovery by homology-directed repair (HDR). Controlled nucleolytic resection of a reversed fork generates a 3′-single-stranded end for RAD51-mediated strand invasion of the duplex ahead of the fork to promote replication restart^49,79^. We investigated how ATM and BRCA1-A status affect fork restart following CPT treatment using DNA fiber assay (Fig. 7a). While ATMi significantly decreased fork restart, ABRAXAS KO partially restored it (Fig. 7b). We further determined DNA fiber tract (IdU) length following replication restart as a read out of fork restart efficiency (Fig. 7c and Supplementary Fig. 12a, b). We observed a rescue of restarted tract length upon ABRAXAS KO, suggesting BRCA1-A loss allows efficient replication resumption of CPT-stalled forks in ATMi treated cells. We further assessed resection kinetics following drug withdrawal in WT control and ABRAXAS KO cells by RPA immunofluorescence (Supplementary Fig. 12c, d). ATM inhibition delayed resection, as revealed by reduced RPA foci after 1 h of continuous CPT (50 nM) treatment, followed by a progressive increase during a 30 min recovery period. This argues that impaired fork reversal causes initial resection delay upon ATM inhibition. Although ABRAXAS KO initially restored resection in ATMi-treated cells, unlike WT cells it did not lead to further increase in RPA signal within the first 30 min of recovery (Supplementary Fig. 12c, d). These results accord well with increased fork restart we observed upon BRCA1-A loss (Fig. 7b, c and Supplementary Fig. 12b) as no further increase in resection during this period presumably reflects ongoing fork reactivation following end resection.

Impaired reversal results in fork breakage due to replication runoff at Top1 cleavage complexes (Top1cc)^46^. NHEJ-mediated illegitimate joining of broken DNA ends leads to chromosomal aberrations, radial chromosomes, and cytotoxicity^44,80^. Because BRCA1-A prevents fork reversal upon ATMi (Fig. 5b), we determined whether this leads to increased fork engagement of NHEJ factors. ATMi increased XRCC4-EdU PLA signal at both low (50 nM) and high (1 µM) CPT dose (Fig. 7d). However, ABRAXAS KO suppressed XRCC4 fork abundance in ATM-inhibited cells. Consistent with previous reports in *ATM*-null mouse cells, metaphase chromosome analysis further revealed a striking increase in chromosomal aberrations in ATMi treated cells, with a high abundance of radial structures (Figs. 7e, 7f). These were significantly reduced following ABRAXAS KO (Fig. 7e, f). ATMi-induced chromosomal aberrations were also found to be reduced in cells expressing RAP80 lacking both SIM and UIM domains (Supplementary Fig. 12e). These results are consistent with a model that BRCA1- A recognition of damaged forks in ATMi treated cells drives heightened chromosomal aberrations via toxic NHEJ (Fig. 7g).

## Discussion

ATM kinase activity is central to DNA repair and required for cell survival following replication damage^39,81–84^. Accordingly, ATM inhibition produces cellular hypersensitivity to fork damaging agents, Top1i or PARPi, and these combinations are currently being investigated in clinical trials (NCT02588105). In this study, we show that BRCA1-A prevents fork reversal in ATM-deficient cells and restricts the generation of end resection substrates required for damaged fork repair. Our results support a model where ATMi promotes BRCA1-A engagement at damaged forks via SUMO and ubiquitin recognition, resulting in fork reversal blockade and eventual fork collapse (Fig. 7g). This engages toxic NHEJ at collapsed forks leading to chromosomal aberrations and cell death. Conversely, BRCA1-A loss restores fork reversal catalyzed by FBH1, HLTF or ZRANB3 ATP-dependent translocases, thereby generating substrates for controlled nucleolytic processing and subsequent fork recovery by homology-directed repair (HDR).

Loss of either NHEJ factors or BRCA1-A suppresses drug hypersensitivity in ATM-deficient cells, suggesting a critical role of BRCA1-A in governing repair outcome following replication fork damage in these genetic settings^35,38–40,43,85,86^. This represents a mechanistically distinct resistance process from BRCA1 deficient cancers where 53BP1 loss confers chemoresistance^44,45^. It is noteworthy that BRCA1 plays dichotomous roles in each genetic context, highlighting its versatility in the DNA damage response through differential recognition of chromatin modifications. BRCA1-A prevents resection dependent damaged fork recovery in the context of ATM deficiency whereas 53BP1 loss is required to restore end resection and HR in BRCA1 mutant cells to cause PARPi or Top1i resistance. These differences further suggest that BRCA1-A and 53BP1 recognize different structures at damaged chromatin to suppress end-resection. 53BP1 requires RNF168 dependent H2AK15Ub and histone H4K20Me2 for damage recognition and suppression of end resection by nucleating the Shieldin complex. BRCA1-A localization to damaged forks required dual SUMO-ubiquitin recognition coincident with increased resection and CPT resistance in RAP80 SIM + UIM deleted mutants. BRCA1 interaction with ABRAXAS was also necessary for fork recognition and hypersensitivity to CPT+ ATMi. This implicates BRCA1-BARD1 damage recognition elements in association with the A-complex in creating a restrictive chromatin state that suppresses nuclease access and end resection. The BARD1-BRCT interacts with H2AK15Ub, and its Ankyrin repeats bind H4K20Me0 that are deposited on newly replicated chromatin^29,30^. Along with RAP80 binding to SUMO-ubiquitin, BARD1 recognition of unmethylated histone H4 on nascent chromatin may further contribute to the different role of BRCA1-A in comparison to 53BP1 in limiting resection at damaged replication forks.

Our findings provide insights into how ATM regulates chromatin state of replication forks in response to DNA damage. iPOND-MS analysis revealed increased abundance of resection nucleases along with chromatin remodeling factors upon BRCA1-A loss in ATM-inhibited cells. This argues that BRCA1-A prevents fork resection by regulating chromatin accessibility to restrict nuclease access. This notion was buttressed by increased chromatin accessibility at damaged forks upon BRCA1-A loss. ATM signaling promotes chromatin accessibility surrounding DSB sites, particularly those located within heterochromatin^62,63,87^. On the other hand, recent findings suggest that replication stress induces a closed chromatin conformation that facilitates fork recruitment of BRCA1-BARD1 and inhibits nucleolytic processing^64^. We envision that ATM loss results in a chromatin environment that favors BRCA1-A engagement at damaged forks in a manner dependent on increased chromatin SUMOylation and ubiquitination. This in turn creates a less accessible chromatin state aided by multivalent nucleosome interactions and nucleosome bridging mediated by the BRCA1-BARD1 heterodimer in association with the A-Complex^31,88^, thereby restricting nuclease access. This idea is supported by the observed increase in chromatin accessibility together with nuclease enrichment at damaged forks upon BRCA1-A loss in ATM deficient cells. The enhanced end resection and resistance to combined CPT and ATMi treatment in BRCA1-A deficient cells were alleviated upon genetic deletion of MRE11 and EXO1 nucleases. This agrees with recent findings demonstrating reliance of BRCA1 but not BRCA2 mutated cancer cells on EXO1 nuclease for survival^89,90^. Although mechanistically distinct, our results further establish restored end resection as a key determinant of viability in both BRCA1 and ATM mutated cancers^39,44^.

ATM plays a poorly understood role in promoting end resection at damaged forks^40,43,91,92^. Using electron microscopy, we provide direct experimental evidence of how damaged replication fork reversal is modulated by the ATM and BRCA1-A status. ATMi markedly reduced fork reversal, whereas BRCA1-A loss substantially restored it in ATM inhibited cells. Thus, BRCA1-A loss generates reversed fork substrates for resection in ATM-deficient cells. This raises important questions regarding the physiologic regulation of BRCA1-A by ATM in normal somatic cells in the absence of drug treatment. We speculate that BRCA1-A prevents inappropriate fork reversal during normal replication. ATM kinase activation at endogenously damaged forks would serve to remove BRCA1-A to enable fork reversal and subsequent resection dependent damage repair. Our observations also present non-mutually exclusive possibilities that ATM directs fork reversal via either removal of BRCA1-A by direct phosphorylation or through phosphorylation of other substrates that affect fork SUMOylation, ubiquitylation and chromatin state^13,19,20,55,93–98^. This also highlights how generation of resection substrates following replication stress is regulated in a manner that depends on different genetic contexts^99^.

Reversed forks are transient intermediates that rapidly restore back by reverse branch migration or undergo resection-dependent fork restart by HR^49,50,79,100^. Thus, fork reversal prevents replication fork collapse as well as unrestrained replication via PRIMPOL-mediated repriming that generates post replicative ssDNA gaps^68,101^. ATM inhibition resulted in compromised fork restart and a concomitant increase in PRIMPOL-dependent ssDNA gaps which were found to be suppressed upon BRCA1-A loss. These observations support a model that fork reversal blockade by BRCA1-A upon ATM kinase inhibition causes replication fork collapse, while leading to some forks being subject to continued replication via PRIMPOL resulting in accumulation of ssDNA gaps.

DNA end resection at stressed forks could be either physiological or pathological, depending on genetic context^49,51,65,70,102,103^. Extended nucleolytic degradation of reversed forks has been linked to chemotherapy response in BRCA1/2-deficient cells^103^. We show that nucleolytic processing of reversed forks upon BRCA1-A loss elicits a pro-survival response in ATM-deficient backgrounds. This agrees with published results demonstrating that controlled nucleolytic resection of reversed forks is indispensable for damage repair and replication restart^48,49,65^. Thus, stalled fork processing operates in a mechanistically distinct manner in BRCA1/2-deficient cells in comparison to cells that experience combined loss of ATM and BRCA1-A. BRCA1/2 deficiency results in extensive degradation of unprotected forks leading to chromosomal aberrations and chemosensitivity. In contrast, BRCA1-A loss in ATM-deficient cells restores resection substrates via fork reversal, thereby promoting restart-enabling limited resection and cell viability. Consequently, fork reversal is a critical determinant of genome stability in ATM deficient cells and may represent a therapeutic target to either reduce or exacerbate replication stress in ATM mutated individuals and associated cancers, respectively.

## Methods

### Cell lines

HT-29 (HTB-38, ATCC) and HEK293T (CRL-3216, ATCC) cell lines were purchased from ATCC. Cell lines were cultured in 1X Dulbecco’s modified Eagle’s medium (GIBCO) supplemented with 10% bovine calf serum (Corning) and 1% Pen Strep (Gibco) at 37 °C with 5% CO_2_. SMARCAL1, ZRANB3, HLTF and FBH1 KO U2OS cell lines were kindly provided by David Cortez, Vanderbilt University School of Medicine.

### CRISPR-Cas9 gene editing

CRISPR-based gene knockout was carried out using either *Streptococcus pyogenes* Cas9 (SpCas9) or *Staphylococcus aureus* Cas9 (SaCas9) system. For SpCas9, stable cell lines expressing SpCas9 were first generated by lentiviral transduction with lenti-SpCas9 hygro (Addgene, plasmid #104995), and subsequent selection with hygromycin (500 µg/ml). sgRNA guides were cloned into either lentiGuide-Puro (Addgene, plasmid #52963) or lentiGuide-neo^104^ (U6-sgRNA-P2A-Neo, reconstructed from Addgene plasmid #52963) vector digested with BsmBI-v2 (New England Biolabs) following the protocol described previously^105^. sgRNA plasmids (2.7 µg) were packaged into lentivirus particles by co-transfecting with packaging plasmids psPAX2 (2 µg, Addgene, plasmid #12260) and pMD2.G (1.3 µg, Addgene, plasmid #12259) into HEK293T cells grown in a 6-well plate using lipofectamine 2000 (Invitrogen). Lentivirus supernatant was collected 48 h post-transfection, filtered through a 0.45-µm filter (Millipore) and subsequently used to transduce the target cells using polybrene (8 µg/ml, Sigma-Aldrich) by spin infection at 931 × *g* for 90 min. Stable transduced cells were selected by either puromycin (4 µg/ml) or neomycin (800 µg/ml), and knockout efficiencies were verified by Western blotting. ABRAXAS KO HEK293T cells were generated by transducing spCas9-expressing (lenti-SpCas9 hygro) stable HEK293T cells with lentivirus particles carrying ABRAXAS sgRNA guides cloned into lentiGuide-Puro plasmid. ABRAXAS or RAP80 KO HT-29 cells were generated by infecting spCas9-expressing stable HT-29 cells with lentivirus particles harboring respective guide RNAs cloned into lentiGuide-neo vector. For generating ABRAXAS KO HT-29 cells using the SaCas9 system, cells were transduced with lentivirus particles carrying ABRAXAS sgRNA guides cloned into SaLgCn (U6-sgRNA-EFS-SaCas9-P2A-Neo^80^) plasmid engineered to express both SaCas9 and sgRNA. Control sgROSA and sgHPRT1 harboring cell lines were generated using SpCas9 and SaCas9 systems, respectively. To make ATM-ABRAXAS, MRE11-ABRAXAS, EXO1-ABRAXAS and PRIMPOL-ABRAXAS double KO cells, ABRAXAS was knocked out using SaCas9, while SpCas9 system was used to KO the second gene of interest. ABRAXAS was KO in SMARCAL1, ZRANB3, HLTF or FBH1 KO U2OS cells using SaCas9 system^99^. For MRE11 knockout, experiments were conducted 48 h following puromycin selection. sgRNA guide sequences are provided in Supplementary Table 1.

### Site-directed mutagenesis and generation of stable cell lines

ABRAXAS and RAP80 mutants were generated by site-directed mutagenesis of pOZ-N-FH ABRAXAS^106^ (Addgene, Plasmid #27495) and pOZ-N-FH RAP80^106^ (Addgene, Plasmid #27501) retrovirus expression plasmids, respectively using Q5 Site-Directed Mutagenesis kit (#E0554S, New England Biolabs). Retrovirus particles were generated by co-transfection of the pOZ-expression plasmids and pCL-Ampho packaging vector with 2:1 ratio into Phoenix cells using lipofectamine 2000 (#11668027, Invitrogen). Retrovirus supernatant was collected 48 h post-transfection and filtered through a 0.45 µm filter. ABRAXAS or RAP80 KO cells were transduced with pOZ-retrovirus particles using polybrene (8 µg/ml, Sigma-Aldrich) by spin infection at 931 x *g* for 90 min. Stably transduced cells were selected on anti-IL2-receptor antibody coated magnetic beads 48 h after virus infection and overexpression was confirmed by Western blotting.

### siRNA transfection

HT-29 cells were transfected two consecutive days with either non-targeting control siRNA (D-001810-01-05, ON-TARGETplus, Horizon Discovery) or siRNA targeting RAD51 (L-003530-00-0005, ON-TARGETplus, Horizon Discovery), FBH1 (M-017404-00-0005, siGENOME, Horizon Discovery) or BRCA1 (M-003461-02-0005, siGENOME, Horizon Discovery) at a final concentration of 100 nM using DharmaFECT 1 transfection reagent (T-2001-02, Horizon Discovery). Cells were fixed for immunofluorescence experiments 72 h after the 1st transfection and knockdown was verified by Western blotting.

### iPOND mass spectrometry

iPOND was performed as described previously with modifications^107^. Control (sgROSA) and ABRAXAS KO HEK293T cells (~100 × 10^6^ cells for each genotype) treated with 1 µM CPT (S1288, Selleck Chemicals) plus 250 nM ATMi (AZD0156, S8375, Selleck Chemicals) were pulse labeled with 10 µM EdU (A10044, Thermo Scientific) for 20 min. ATMi was added 5 min before CPT treatment and present throughout the experiment. An additional set of control cells were kept for no-click negative control. Cells were fixed immediately with 1% formaldehyde in 1X PBS for 20 min at RT followed by glycine (1.25 M) quenching. Cells were collected by scraping and pelleted by centrifugation at 900 × *g* for 5 min at 4 °C. Cells were washed three times with 1X ice-cold PBS followed by permeabilization with 0.25% Triton X-100 in PBS for 30 min at RT. Cells were pelleted by centrifugation at 900 × *g* for 5 min at 4 °C and washed with 0.5% BSA in 1X PBS. Cell pellets were resuspended in click reaction cocktails containing 20 μM biotin-azide (B10184, Thermo Scientific), 2 mM CuSO4 (C7631, Sigma Aldrich), 10 mM sodium L-ascorbate (A4034, Sigma Aldrich) in 1X PBS, vortexed and incubated for 2 h at RT with rotation. For no-click negative control, biotin-azide was not included in the click reaction cocktails. Cells were pelleted by centrifugation at 900 × *g* for 5 min at 4 °C and washed with 0.5% BSA in 1X PBS. Cell pellets were resuspended in lysis buffer (1% SDS in 50 mM Tris-HCl, pH 8.0) at a concentration of 100 × 10^6^ cells per 4 ml and sonicated using a microtip sonicator (Branson 150) set at 35% amplitude with 5 pulses (20 S on and 40 S pause). Lysates were clarified twice by centrifugation at 16,100 × *g* for 10 min at RT, filtered through an 80 µm nylon membrane (NY8004700, Millipore Sigma) and diluted 1:1 (v/v) with 1X ice-cold PBS containing protease inhibitors. Lysates were incubated with streptavidin agarose beads (#69203, Millipore Sigma) at a concentration of 50 µl (packed volume) beads per 100 × 10^6^ cells overnight at RT with rotation. Beads were washed once with cold lysis buffer, once with 1 M NaCl and final two washes with cold lysis buffer. Proteins were eluted by boiling the beads in 4X LDS sample buffer (NP0007, Thermo Scientific) at 95 °C, and iPOND purification was evaluated by either silver staining or Western blotting using antibodies for replication fork associated proteins.

Mass spectrometry analysis of iPOND purified protein samples (*n* = 5) was carried out in Taplin Biological Mass Spectrometry Facility, Harvard Medical School. Beads were washed at least five times with 100 µl 50 mM ammonium bicarbonate then 5 µl (200 ng/µl) of modified sequencing-grade trypsin (Promega, Madison, WI) was spiked in and the samples were placed in a 37 °C room overnight. The samples were then centrifuged and the liquid removed. The extracts were then dried in a speed-vac (~1 h). Samples were re-suspended in 50 µl of HPLC solvent A (2.5% acetonitrile, 0.1% formic acid) and desalted by STAGE tip^108^. On the day of analysis, the samples were reconstituted in 10 µl of HPLC solvent A. A nano-scale reverse-phase HPLC capillary column was created by packing 2.6 µm C18 spherical silica beads into a fused silica capillary (100 µm inner diameter x ~ 30 cm length) with a flame-drawn tip^109^. After equilibrating the column, each sample was loaded via a Famos auto sampler (LC Packings, San Francisco CA) onto the column. A gradient was formed, and peptides were eluted with increasing concentrations of solvent B (97.5% acetonitrile, 0.1% formic acid). As peptides eluted, they were subjected to electrospray ionization and then entered into a Velos Orbitrap Elite ion-trap mass spectrometer (Thermo Fisher Scientific, Waltham, MA). Peptides were detected, isolated, and fragmented to produce a tandem mass spectrum of specific fragment ions for each peptide. Peptide sequences (and hence protein identity) were determined by matching protein databases with the acquired fragmentation pattern by the software program, Sequest (Thermo Fisher Scientific, Waltham, MA)^110^. All databases include a reversed version of all the sequences and the data was filtered to between one and two percent peptide false discovery rate. For fold change calculation, normalization was done by multiplying each spectrum count in a sample by the average number of spectra across all samples/total number of spectra in the respective sample. Proteins with ≥ 6 peptide count detected in total in control and ABRAXAS KO cells across all replicates and showed > 5 average fold change in iPOND samples over no click (NC) negative control were considered for fold change analysis. Log2 fold change was calculated from *n* = 5 biological replicates and scatter plot was generated in GraphPad Prism 10. iPOND mass spectrometry data is provided in Supplementary Data 1 and 2. The mass spectrometry proteomics data have been deposited to the ProteomeXchange Consortium via the PRIDE^111^ partner repository with the dataset identifier PXD077767.

### Gene ontology (GO) analysis

Gene ontology analysis of proteins identified from iPOND-MS analysis was done using GeneCodis 4^112^. Proteins enriched by ≥ 1.20-fold in ABRAXAS KO cells over control (sgROSA) cells were analyzed for GO biological processes and GO molecular functions. GO enrichment analysis data are provided in Supplementary Data 3.

### Colony survival assay

Cells were plated on 6-well (~1500) or 12-well (~1000) cell culture plates and treated with CPT and ATMi 4 h after cell seeding. Cells were allowed to form colonies for two to three weeks. After 1X PBS wash, colonies were fixed with 100% methanol, subsequently stained with staining solution (25% methanol+ 0.5% crystal violet). The total number of colonies was normalized with plating efficiency and relative colony survival was determined as percentage of colonies survived following drug treatments. Graphs were generated in MS Excel Version 2605 Build 16.0.20026.20166.

### Immunofluorescence

Cells grown onto coverslips or 8-well chamber slides (#229168, Celltreat) were treated with CPT and ATMi as indicated in the figures. For experiments using EdU incorporation, cells were concomitantly labeled with EdU (10 µM) to identify S-phase cells. Cells were washed twice with ice-cold 1X PBS followed by nuclear pre-extraction for 10 min on ice using CSK buffer (10 mM PIPES, pH 7.0; 100 mM NaCl; 300 mM Sucrose; 3 mM MgCl_2_; 1 mM EGTA, 0.5% Triton X-100). For EdU detection, cells were subjected to click reaction using click reaction cocktails containing 20 μM Alexa Fluor™ 488 Azide (A10266, Thermo Scientific), 2 mM CuSO_4_, 100 mM sodium L-ascorbate diluted in 1X PBS for 1 h at RT. Cells were washed twice with 1X PBS and fixed with 4% paraformaldehyde (#50-980-495, Electron Microscopy Sciences) diluted in 1X PBS solution for 20 min on ice. Cells were washed three times with ice-cold 1X PBS, permeabilized with 0.5% Triton X-100 for 10 min on ice and incubated in blocking buffer (5% BSA with 10% goat serum in 1X PBS) at 4 °C for 30 min. Cells were incubated in primary antibodies diluted in blocking buffer overnight at 4 °C. Cells were counterstained with γH2AX to mark S-phase cells that acquire replication fork damage due to fork collision with CPT-induced TOP1-DNA cleavage complexes. Cells were washed three times with 1X PBS followed by incubation in AlexaFluor 647- or AlexaFluor 488-conjugated secondary antibodies (Thermo Scientific) for 1 h at RT. Cells were washed three times with 1X PBS, stained with DAPI (D9542, Sigma-Aldrich, 4 µg/ml diluted in 1X PBS) and mounted with ProLong Gold Antifade mountant (P36930, Thermo Scientific). Immunofluorescence images were acquired by CoolSnap MYO camera of Nikon Eclipse 80i fluorescence microscope using a Plan Apo VC 60× Oil DIC N2 objective lens. Images captured with 100X magnification were acquired using a 100X oil objective lens of Nikon Eclipse Ti2 microscope. Images were quantified using CellProfiler 4.2.5 image analysis software^113^. Nuclei with γH2AX or EdU intensity above background were scored positive for γH2AX or EdU respectively using “Two-class OTSU” thresholding method in CellProfiler 4.2.5. Graphs were generated in GraphPad Prism 10. Antibodies used in immunofluorescence experiments are listed in Supplementary Table 2.

### BrdU-coupled MNase accessibility assay

HEK293T cells were treated with CPT (1 µM) and ATMi (500 nM) and pulse labeled with 20 µM BrdU for 12 min. Cells were washed with 1X ice-cold PBS, trypsinized and pelleted by centrifugation at 300 × *g* for 10 min at 4 °C. Equal number of cells (~30 × 10^6^) in each sample were resuspended in 5 ml of ice-cold NP-40 lysis buffer (10 mM TrisHCl pH 7.4, 10 mM NaCl, 3 mM MgCl2, 0.5% Nonidet P-40, 0.15 mM spermine, and 0.5 mM spermidine), incubated on ice for 5 min and centrifuged at 120 × g for 10 min at 4 °C. The nuclei were washed with 2.5 ml of MNase digestion buffer (10 mM Tris-HCl pH 7.4, 15 mM NaCl, 60 mM KCl, 0.15 mM spermine and 0.5 mM spermidine) and pelleted by centrifugation for 10 min 4 °C. The nuclei were resuspended in 1 ml MNase digestion buffer containing 1 mM CaCl_2_, and 100 µl of resuspended nuclei were subjected to MNase (250 gel units) digestion at 37 °C for the time periods as specified in the respective figures. Reactions were stopped by adding MNase stop buffer (100 mM EDTA and 10 mM EGTA pH 7.5) diluted 1:5 in digestion buffer. Samples were incubated at 37 °C overnight after the addition of 3 µl of proteinase K (25 mg/ml., Invitrogen, EO0491) and 10 µl of 20% SDS. Genomic DNA was purified by Phenol chloroform extraction methods and ethanol precipitation. Equal amount of DNA (4 µg) in each sample was run on 1.2% agarose gel, stained with Ethidium Bromide (EtBr) and photographed using a GelDoc system (Biorad). The gel was treated with 0.2 N HCL for 10 min, washed several times in water and treated with denaturation solution (1.5 M NaCl, 0.5 M NaOH) for 30 min with gentle rocking. The gel was neutralized by neutralization solution (3 M NaCl, 0.5 M Tris pH 7.5) for 30 min and subjected to Southern transfer to Hybond-N+ charged nylon membrane (RPN203B, Cytiva) using 20X SSC buffer (3 M NaCl, 0.3 M sodium citrate) at RT overnight. Membrane was crosslinked in a UVP crosslinker (120 mJ/cm^2^), blocked in 5% milk for 1 h and incubated in anti-BrdU antibody (1:200, #347580, BD Biosciences) overnight at 4 °C. Membrane was washed three times with 1X PBST (Phosphate Buffer Saline with 0.1% Tween 20), incubated in anti-mouse secondary antibody for 1 h at RT and washed 5 times with 1X PBST. BrdU labeled DNA fragments were detected using Western lightning plus-ECL (NEL105001EA, Revvity Health Sciences Inc.). BrdU labeled nucleosomes were quantified in ImageJ (https://imagej.net/ij/) and MNase accessibility was determined by fractions of BrdU labeled mononucleosomes as a percentage of total BrdU-labeled DNA in each lane. Graphs were generated in GraphPad Prism 10.

### SIRF assay

SIRF assay was performed as described previously with modifications^114,115^. HT-29 cells were plated in 8-well chamber slides at 60% confluency the day before the experiments. Cells were optionally pretreated with 250 nM ATMi for 10 min followed by incubation in CPT (1 µM or 50 nM) containing medium for 40 min. Cells were pulse labeled with 125 µM of EdU (A10044, Invitrogen) 20 min prior to the end of CPT treatment. ATM inhibitor was present during the entire experiment. In experiments using SUMO E1 inhibitor, cells were pre-treated with 1 µM ML-792 (S8697, Selleck Chemicals) for 16 h before CPT + /- ATMi addition. Cells were washed two times with ice-cold 1X PBS followed by pre-extraction with CSK buffer (10 mM PIPES, pH 7.0; 100 mM NaCl; 300 mM Sucrose; 3 mM MgCl_2_; 1 mM EGTA, 0.5% Triton X-100) for 10 min on ice. Cells were fixed with 2 % paraformaldehyde for 15 min at RT. Cells were washed two times with ice-cold 1X PBS and permeabilized with 0.25% Triton X-100 for 15 min at RT. After washing two times with 1X PBS, cells were subjected to click reaction using 20 μM biotin-azide, 2 mM CuSO4, 100 mM sodium L-ascorbate diluted in 1X PBS for 1 h at RT. After washing with 1X PBS, slides were blocked with blocking buffer (5% BSA with 10% goat serum in 1X PBS) for 1 h at 4 °C followed by primary antibody incubation at 4 °C overnight. Primary antibodies used in SIRF immunofluorescence experiments are listed in Supplementary Table 2. An EdU-EdU SIRF was included to determine the total EdU incorporation in each experimental condition using mouse anti-biotin and rabbit anti-biotin antibodies. Slides were washed three times with wash buffer A (0.01 M Tris, 0.15 M NaCl, and 0.05% Tween 20, pH 7.4) followed by incubation with anti–mouse plus and anti–rabbit minus PLA probes diluted 1:5 in blocking solution for 1 h at 37 °C. After three washes in wash buffer A, ligation reaction was performed at 37 °C for 30 min using Duolink ligation buffer (diluted 1:5 in water) and ligase (diluted 1:40 in water) enzymes provided with Duolink® In Situ Detection Reagents Red kit (DUO92008, Sigma-Aldrich). Slides were briefly washed two times with wash buffer A and subjected to rolling circle amplification at 37 °C for 100 min using amplification mix containing Duolink amplification reagent (diluted 1:5 in water) and rolling circle polymerase (diluted 1:80 in water). Slides were washed three times with wash buffer B (0.2 M Tris and 0.1 M NaCl) and one time with 0.01× diluted wash buffer B. Slides were stained with DAPI (D9542, Sigma Aldrich, 4 µg/ml diluted in 1X PBS) and mounted with ProLong Gold Antifade mountant (P36930, Thermo Scientific). Images were acquired by Nikon Eclipse Ti2 fluorescence microscope using a 100× Oil objective lens or a 60× Oil DIC N2 objective lens of Nikon Eclipse 80i fluorescence microscope. SIRF-PLA foci or intensity were quantified using CellProfiler 4.2.5 software and normalized with the average EdU-EdU signal in each sample. Scatter plots were generated in GraphPad Prism 10.

### Electron microscopy analysis

EM analysis of replication intermediates was performed as described previously^73,116,117^. Briefly, control and ABRAXAS KO HT-29 cells (~60 × 10^6^ cells in each sample) were treated with 50 nM CPT + /− 250 nM ATMi for 1 h. Cells were washed with 1X ice cold PBS, collected by trypsinization and pelleted by centrifugation at 931 × *g* for 5 min at 4 °C. Cells were resuspended in 1X ice-cold PBS, transferred to 60 mm tissue culture dishes placed on a cold metal block. Genomic DNA was psoralene-crosslinked by addition of 4,5′,8-Trimethylpsoralen (TMP, T6137, Sigma Aldrich) in a spread-out fashion at a final concentration of 10 µg/ml and irradiated in a UVP crosslinker at 120 mJ/cm^2^ for 3 min. The psoralene-crosslinking was repeated two more times and cells were pelleted by centrifugation at 931 × *g* for 5 min at 4 °C. Cells were washed three times with 1X ice-cold PBS, resuspended in cold lysis buffer (1.28 M Sucrose, 40 mM Tris-HCl pH 7.5, 20 mM MgCl2, 4% Triton X-100; diluted 1:4 with water) in a 1:4 ratio and incubated on ice for 10 min. Lysed cells were pelleted by centrifugation at 524 × *g* for 15 min at 4 °C. After washing once with cold lysis buffer, nuclei were resuspended in 200 µl cold 1X PBS and subsequently resuspended in 5 ml of digestion buffer (800 mM guanidine-HCl, 30 mM Tris-HCl pH 8.0, 30 mM EDTA pH 8.0, 5% Tween-20, 0.5% Triton X-100). Samples were subjected to Proteinase K (EO0491, Invitrogen) digestion (200 µl of 20 mg/ml) at 50 °C for 2 h. Genomic DNA was extracted by standard chloroform:isoamylalcohol and isopropanol precipitation method and digested with PvuII HF (R3151S, New England Biolabs) restriction enzyme at 37 °C for 4 h. Replication intermediates were enriched on plasmid miniprep columns (#12123, QIAGEN) and subsequently concentrated using Amicon Ultra size exclusion columns (UFC510096, Millipore Sigma). For EM visualization, samples were prepared by spreading the concentrated DNA on a carbon-coated grid in presence of benzyl-dimethyl-alkylammonium chloride (B6295, Millipore Sigma) as well as n,n-dimethylformamide (#227056, Millipore Sigma), followed by low angle platinum rotary shadowing. Images were acquired with a JEOL JEM-1400 electron microscope using a bottom mounted AMT XR401 camera. Image analysis was done using ImageJ software^118^. EM analysis allows distinguishing duplex DNA from ssDNA. DNA duplex is expected to appear as a 10 nm thick fiber following platinum coating step necessary for EM visualization, whereas ssDNA has a reduced thickness of 5–7 nm and appears lighter in contrast to that of dsDNA. Criteria used for the assignment of a three-way junction, indicative of a typical replication fork, include the joining of three DNA fibers into a single junction, with two generally symmetrical daughter strands and a single parental strand. Reversed replication forks comprise four DNA strands joined at a single junction. This consists of two commonly symmetrical daughter strands, one parental strand and an additional typically shorter fourth strand, representative of the reversed arm. The length of the two daughter strands corresponding to the newly replicated duplex oftentimes are equal (b = c), whereas the length of the parental arm and the regressed arm can vary (a ≠ b = c ≠ d). Conversely, canonical Holliday junction structures are characterized by arms of equal length (a = b, c = d). Particular attention was given to the junction of the reversed replication fork to observe the presence of a bubble structure, indicating that the junction is opened and that it was simply not the result of the occasional crossover of two DNA molecules. These four-way junctions of reversed replication forks may also be collapsed and other indicators such as daughter strand symmetry, presence of single-stranded DNA at the junction or the entire structure itself, all are considered during analysis^117^. The frequency of reversed forks, junction gaps and daughter strand gaps in a sample was computed using Prism software.

### Detection of nascent ssDNA by IdU labeling under non-denaturing conditions

To detect nascent ssDNA, cells were treated with CPT +/- ATMi and concomitantly labeled with 250 µM IdU. Drug incubation and IdU labeling time is indicated in respective figures and legends. Nuclei were pre-extracted, fixed and permeabilized as described under Immunofluorescence method section. After incubation in blocking buffer (5% BSA with 10% goat serum in 1X PBS) for 30 min at 4 °C, fixed cells were incubated with mouse anti-IdU (1:100, #347580, BD Pharmigen) and rabbit anti-γH2AX (1:500, #39117, Active Motif) antibodies diluted in blocking buffer overnight at 4 °C. After three PBS wash, cells were incubated with anti-mouse AlexaFluor 488 (1:500, A-11029, Thermo Scientific) and anti-rabbit AlexaFluor 647 (1:500, A-21245, Thermo Scientific) conjugated secondary antibodies for 1 h at RT. Cells were washed with 1X PBS, stained with DAPI (D9542, Sigma-Aldrich, 4 µg/ml diluted in 1X PBS) and mounted using ProLong Gold Antifade mountant (P36930, Thermo Scientific). IdU immunofluorescence was examined by Nikon Eclipse 80i fluorescence microscope using a 60× Oil DIC N2 objective lens or Nikon Eclipse Ti2 fluorescence microscope equipped with a 100× oil objective lens. Images were quantified using CellProfiler 4.2.5 software and scatter plots were generated in GraphPad Prism 10.

### DNA fiber assay

DNA fiber assay was performed as described previously^119^. Briefly, exponentially growing HT-29 cells (~0.2 × 10^6^) were sequentially labeled with 25 μM CldU (C6891, Sigma Aldrich) and 250 μM IdU (I7125, Sigma Aldrich), and treated with CPT, ATMi or hydroxyurea (H8627, Sigma Aldrich) as indicated in the respective figures and legends. Cells were washed with ice-cold 1X PBS, trypsinized, and ~2000 cells were lysed using lysis buffer (200 mM Tris-HCl pH 7.4, 50 mM EDTA and 0.5% SDS) on a silane coated slide (#5070, Newcomer Supply). DNA fibers were spread by gravity flow, air dried and fixed in fixative solution (Methanol: Acetic acid, 3:1). Slides were treated with 2.5 M HCl for 1 h at RT, neutralized with 400 mM Tris-HCl, pH 7.4. After PBST wash, slides were blocked with blocking buffer (5% BSA and 10% goat serum in 1X PBS) at 4 °C overnight. Slides were incubated with rat anti-CIdU antibody (1:100, ab6326, Abcam) for 1 h at RT. After three PBST wash, slides were incubated with AlexaFluor 647-conjugated anti-rat secondary antibody (1:100, A-21247, ThermoFisher Scientific) for 1 h at RT and washed three times with PBST. Next, the slides were incubated with mouse anti-BrdU antibody (1: 40, #347580, BD Pharmigen) for 1 h at RT, washed with PBST and subsequently incubated with AlexaFluor 488-conjugated anti-mouse secondary antibody (1:100, A-11029, ThermoFisher Scientific) for 1 h at RT. Slides were washed three times in PBST and mounted with Prolong Gold antifade mountant (P36930, ThermoFisher Scientific). DNA fibers were imaged by Nikon Eclipse 80i fluorescence microscope using a Plan Apo VC 60× Oil DIC N2 objective lens. Fibers were quantified in ImageJ (https://imagej.net/ij/). Fork restart efficiency was determined by fractions of CldU-IdU (red-green) labeled fibers as a percentage of total CldU (red and red-green) labeled fibers. In DNA fiber experiments using hydroxyurea, fork degradation was determined by calculating IdU to CldU tract length ratios (IdU/CldU) of individual DNA fibers. Graphs were generated in GraphPad Prism 10.

### S1 nuclease assay

S1 nuclease assay using DNA fiber technique was performed as described previously^120^. Briefly, asynchronously growing HT-29 cells (~2 × 10^5^) were labeled with CldU (25 μM, C6891, Sigma Aldrich) for 20 min followed by IdU labeling (250 μM, I7125, Sigma Aldrich) for 60 min during which CPT (50 nM) and ATMi (250 nM) was added. Cells were washed with 1X PBS and permeabilized with CSK100 buffer (100 mM NaCl, 10 mM MOPS, pH 7.3  mM MgCl_2_, 300 mM Sucrose, 0.5% Triton-X 100) for 10 min at RT. After permeabilization, cells were washed once with 1X PBS, once with S1 nuclease buffer (30 mM sodium acetate, 10 mM zinc acetate, 5% glycerol, 50 mM NaCl, pH 4.6) and incubated in S1 nuclease buffer containing 20 U/ml S1 nuclease enzyme for 30 min at 37 °C. Cells were collected in 1X PBS + 0.1% BSA by scrapping and centrifuged at 4600 × *g* for 5 min at 4 °C. DNA fiber spreads were prepared, immunostained and analyzed as described for DNA fiber assay. DNA fibers were imaged by Nikon Eclipse 80i fluorescence microscope using a Plan Apo VC 60× Oil DIC N2 objective lens. Graphs were generated in GraphPad Prism 10.

### Metaphase chromosome analysis

Metaphase chromosomes were prepared as described previously^80^. Briefly, HT-29 cells (~5 × 10^6^) growing in 10 cm dishes were treated with 2.5 nM CPT in combination with 50 nM ATMi for 16 h followed by metaphase arrest using KaryoMAX Colcemid solution (0.1 μg/ml) (#15212012, ThermoFisher Scientific) for 6 h. Cells were washed with ice-cold 1X PBS, trypsinized, and collected by centrifugation at 100×g for 5 min. Cell pellets were resuspended in 75 mM prewarmed (37 °C) KCL in a drop-wise manner using slow vortexing followed by incubation at 37 °C for 30 min. Cells were fixed two times by dropwise addition of 6 ml freshly prepared fixative solution (methanol: acetic acid, 3:1) with gentle vortexing. After centrifugation at 100 × *g* for 5 min, cell pellets were resuspended in 2 ml fixative solution and stored at −20 °C overnight. Cells were resuspended in 1 ml freshly prepared fixative solution followed by metaphase chromosome preparation by dropping 200–300 μl of the cell suspension on silane-coated glass slide (#5070, Newcomer Supply) presoaked in 40% acetic acid. The slides were air dried, recovered overnight and stained with Giemsa (48900, Sigma-Aldrich) followed by mounting using Prolong Gold antifade mountant (P36930, ThermoFisher). Images were acquired using Nikon Eclipse Ti2 fluorescence microscope equipped with a 100× Oil objective lens with brightfield settings. Data were analyzed in ImageJ (https://imagej.net/ij/). Graphs were generated in GraphPad Prism 10.

### Western blotting

Cells were lysed in 2X LDS sample buffer (NP0007, Thermo Scientific) supplemented with benzonase (E1014, Sigma-Aldrich) and 1.5 mM MgCl_2_. Proteins were separated onto NuPAGE™ 4–12% Bis-Tris (NP0335BOX, Thermo Scientific) gels, transferred onto 0.2 µm nitrocellulose blotting membrane (#10600004, Cytiva) by wet transfer at 100 constant volts for 2 h at 4 °C. The membranes were blocked with 5% milk diluted in 1X PBS for 1 h followed by primary antibody incubation overnight at 4 °C. Membranes were washed three times with PBST and incubated with HRP-conjugated anti-mouse (NA931, Amersham, 1:2500 dilution) or anti-rabbit (NA934, Amersham, 1:2500 dilution) secondary antibodies at RT for 1 h. Membranes were washed with PBST, and protein bands were detected using Western lightning plus-ECL (NEL105001EA, Revvity Health Sciences Inc.). Antibodies used in Western blotting are listed in Supplementary Table 2. Uncropped and unprocessed scans of all blots are provided in the Source Data file or as Supplementary Figs. in the Supplementary Information.

### Statistics and reproducibility

Unpaired two-tailed *t* test and two-tailed Mann–Whitney test were performed to compute statistical significance in GraphPad Prism 10. All experiments were performed at least twice unless otherwise specified. Sample size and *p* values are indicated in figure legends and within figures.

### Figure preparation

Figures were prepared using Adobe Illustrator 2026 and MS PowerPoint Version 2605 Build 16.0.20026.20166. Schematics depicting experimental strategies were made in MS PowerPoint Version 2605 Build 16.0.20026.20166.

### Reporting summary

Further information on research design is available in the Nature Portfolio Reporting Summary linked to this article.

## Supplementary information

Supplementary Information

Description of Additional Supplementary Files

Supplementary Data 1

Supplementary Data 2

Supplementary Data 3

Reporting Summary

Transparent Peer Review file

## Source data

Source Data

## Data Availability

The iPOND mass spectrometry data generated in this study have been deposited to the ProteomeXchange Consortium via the PRIDE partner repository with the dataset identifier PXD077767. All remaining data are available within the article, supplementary information, and supplementary data files. Source data are provided with this paper.
