# Supplementary material for: The BRCA1-A complex restricts replication fork reversal-dependent DNA repair in ATM deficient cells
Source: Nat Commun. 2026 Jul 4;17:8321. doi: 10.1038/s41467-026-75271-7 (PMC13470386; doi:10.1038/s41467-026-75271-7)

Supplementary information

The BRCA1-A complex restricts replication fork reversal-dependent DNA repair in ATM deficient cells

Arindam Datta[1], Jessica Jackson[2], Yaroslav I Morozov[1], Jinghan Qiu[1], Alessandro Vindigni[2] and Roger A. Greenberg[1,*]

[1]Department of Cancer Biology, Penn Center for Genome Integrity, Basser Center for BRCA, Perelman School of Medicine, University of Pennsylvania, Philadelphia, PA 19104

[2]Division of Oncology, Washington University, St. Louis, MO 63110

*To whom correspondence should be sent:
Roger A. Greenberg: rogergr@pennmedicine.upenn.edu

Supplementary Fig.1

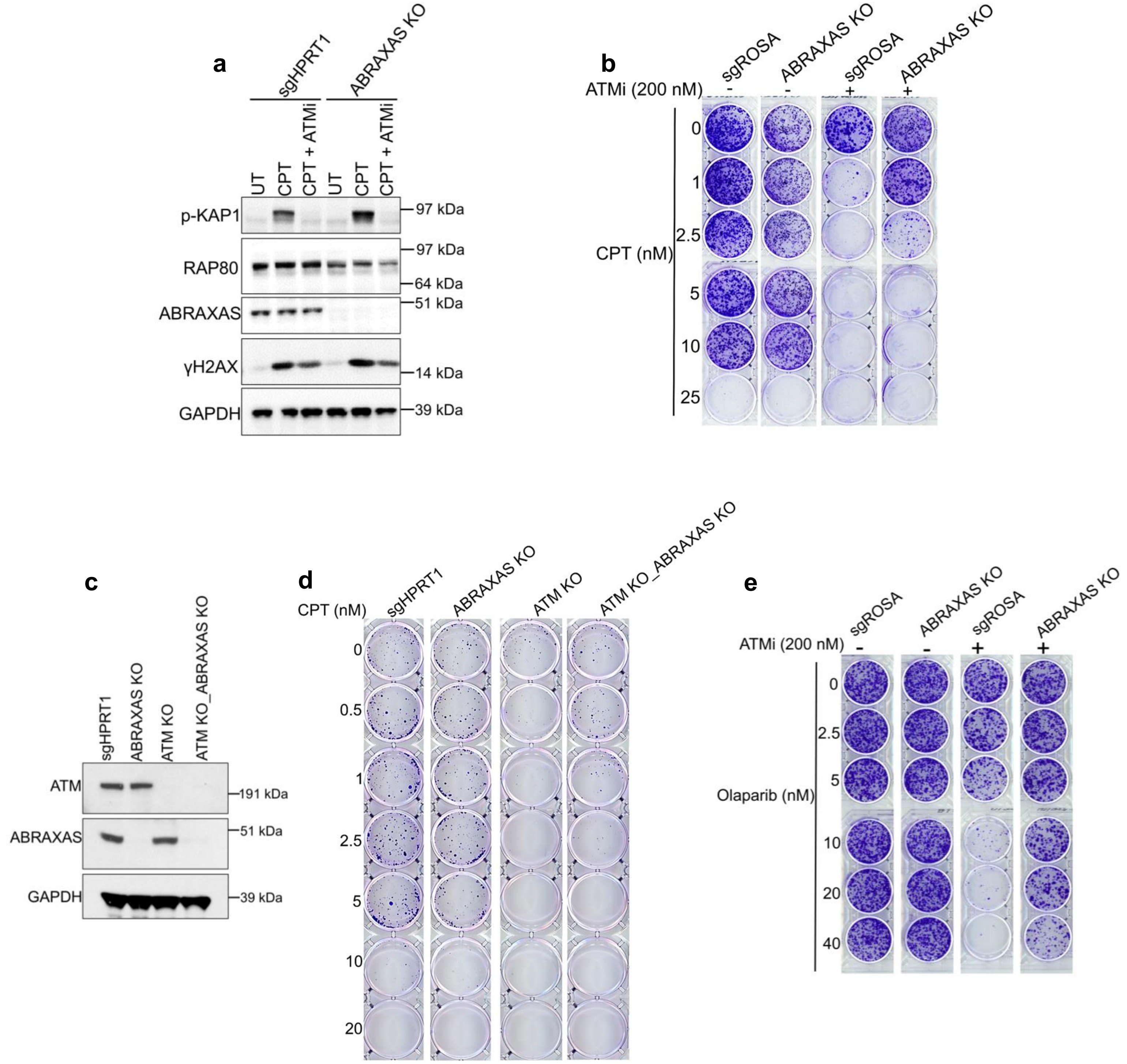

Supplementary Fig. 1. BRCA1-A complex loss confers drug resistance in ATM-deficient cancer cells. a, Western blots showing diminished CPT-induced phospho (S824)-KAP1 (p-KAP1) levels upon ATMi treatment, confirming potency of the ATM inhibitor. Control (sgROSA) and ABRAXAS KO HEK293T cells were treated with CPT (1 µM) +/- ATMi (250 nM) for 40 min and analyzed by Western blotting using the indicated antibodies. GAPDH serves as loading control. RAP80 and γH2AX derived from the same experiment were run on a different gel. Molecular weight markers are shown. Experiments were repeated two times with similar results. **b,** Colony survival assay showing CPT+ATMi resistance upon ABRAXAS KO in HEK293T cells. Experiments were repeated n=3 times with similar results. **c,** Western blots validating ATM and ABRAXAS DKO in HT-29 cells. GAPDH serves as loading control. Molecular weight markers are shown. Experiments were repeated two times with similar results. **d,** Colony survival assay showing CPT resistance in ATM and ABRAXAS DKO HT-29 cells. **e,** Colony survival assay showing olaparib + ATMi resistance in ABRAXAS KO HEK293T cells. Experiments were repeated n=3 times with similar results. In (b, d, and e), experiments were repeated three times with similar results. Source data are provided as a Source Data file.

Supplementary Fig. 2

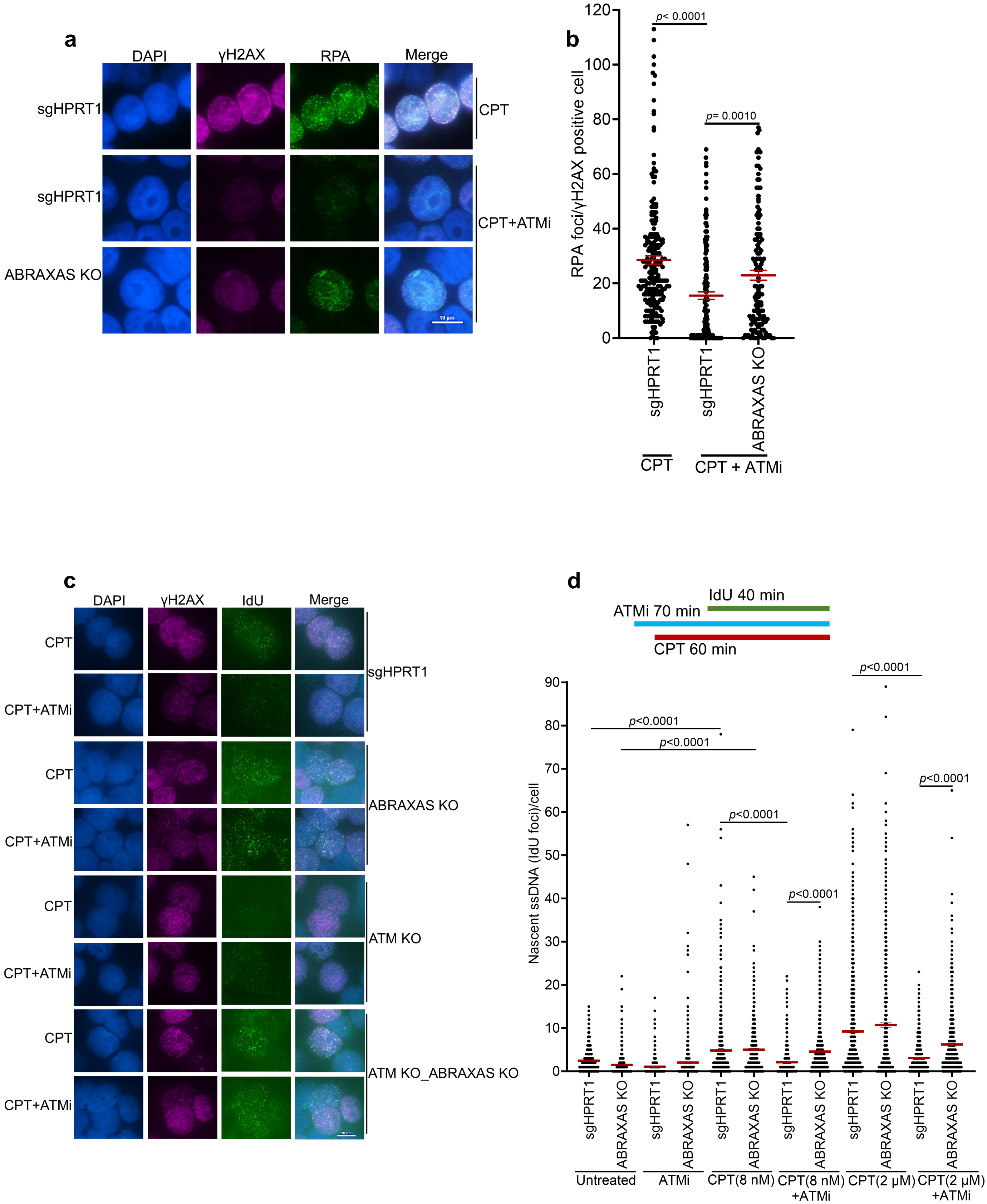

Supplementary Fig. 2. BRCA1-A complex suppresses damaged fork resection upon ATM inhibition. (a and b), Representative RPA immunofluorescence in control (sgHPRT1) and ABRAXAS KO HT-29 cells treated with CPT (1 µM) +/- ATMi (250 nM) for 1 h. ATMi was optionally added 10 min before CPT treatment and was present throughout the experiment. γH2AX immunostaining marks cells with CPT-induced replication damage. Images were acquired with 60X magnification. Scale bar is shown (**a**). Scatter plot showing quantification of RPA foci in γH2AX positive cells (**b**). Data represent mean ± SEM derived from at least 130 γH2AX positive cells (n=3 independent experiments); *p* values ($p < 0.0001$, $p = 0.0010$) are indicated. **c,** Representative images of IdU immunofluorescence detected under non-denaturing conditions in the indicated genotypes of HT-29 cells treated with 50 nM CPT +/- 250 nM ATMi. Images were acquired with 60X magnification. Scale bar is shown. **d,** Schematic showing native IdU assay in control (sgHPRT1) and ABRAXAS KO HT-29 cells treated with a low (8 nM) or high (2 µM) dose of CPT +/- 250 nM ATMi. IdU was added 20 minutes after CPT addition to label newly synthesized DNA. Quantification of ssDNA (native IdU foci) is shown. Data represent mean ± SEM derived from at least 390 cells (n=2 independent experiments); *p* values ($p < 0.0001$, $p < 0.0001$, $p < 0.0001$, $p < 0.0001$, $p < 0.0001$, $p < 0.0001$) are indicated. In (b and d), *p* values were calculated with unpaired two-tailed t test. Source data are provided as a Source Data file.

Supplementary Fig. 3

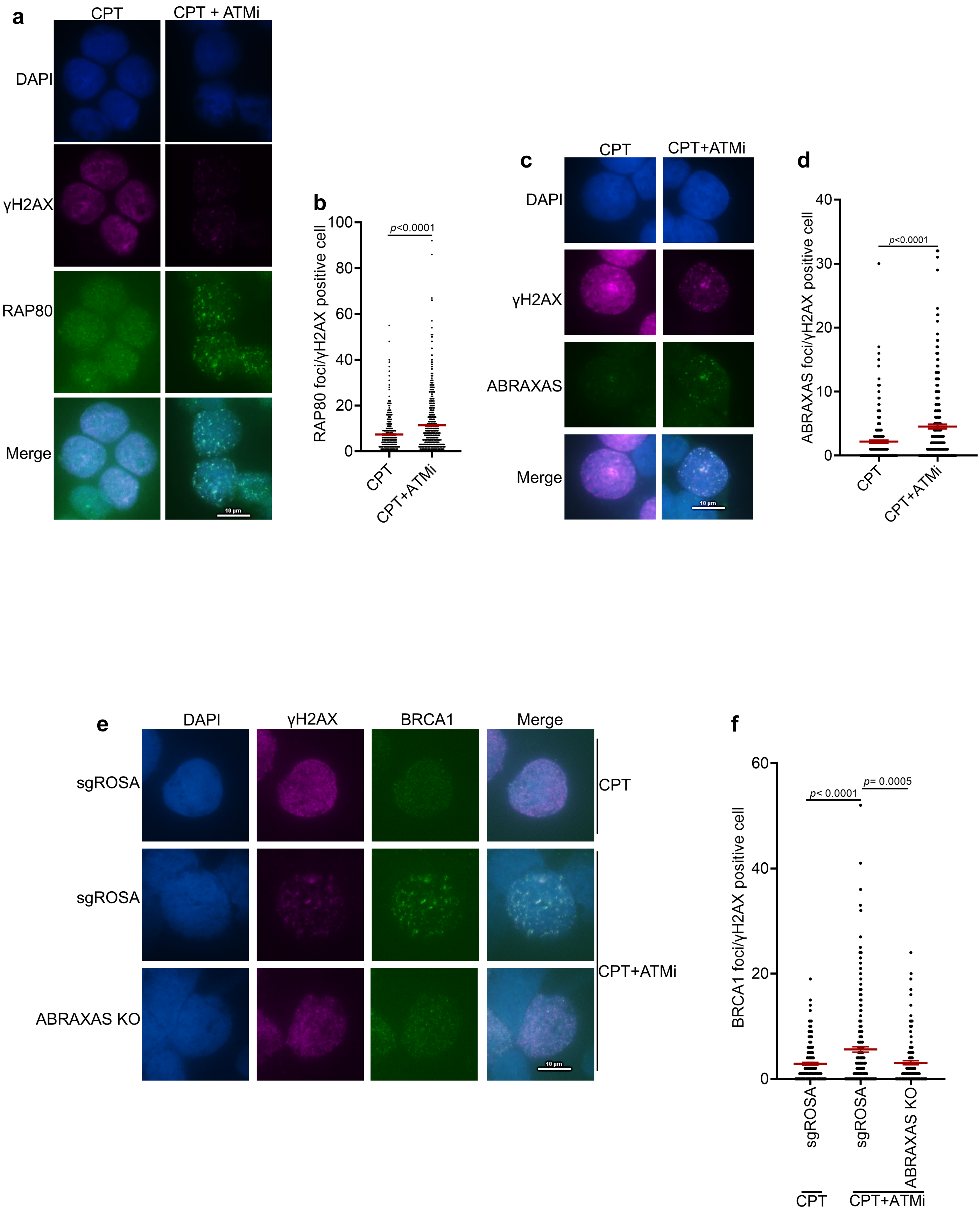

Supplementary Fig. 3. ATMi promotes BRCA1-A complex recruitment to damaged chromatin. (a and **b),** Representative immunofluorescence images of RAP80 foci in HT-29 cells treated with CPT (1 μM) alone or in combination with ATMi (250 nM) for 1 h. ATMi was added 10 min before CPT treatment. γH2AX marks cells with CPT-induced replication damage. Images were acquired with 60X magnification. Scale bar is shown. (**a**). Quantification of the RAP80 foci is shown (**b**). Experiments were performed four times with similar results. Data represent mean ± SEM derived from at least 325 cells (n=2 independent experiments); *p* values ($p < 0.0001$) are indicated. **c,** Representative immunofluorescence images showing increased HA-ABRAXAS foci in HT-29 cells upon ATM inhibition. Cells were treated with CPT (1 μM) +/- ATMi (250 nM) for 1 h. ATMi was added 10 min before CPT treatment. γH2AX marks cells with CPT-induced replication damage. Images were acquired with 60X magnification. Scale bar is shown. **d,** Quantification of the data from experiments as described in (c). Experiments were repeated three times with similar results. Data are mean ± SEM derived from at least 300 cells (n=2 independent experiments); *p* values ($p < 0.0001$) are indicated. **(e** and **f),** Representative immunofluorescence images of BRCA1 foci in control (sgROSA) and ABRAXAS KO HT-29 cells treated with CPT (1 μM) +/- ATMi (250 nM) for 1 h. ATMi was added 10 min before CPT treatment. Images were acquired with 60X magnification. Scale bar is shown. (**e**). Scatter plot showing quantification of the data (**f**). Data represent mean ± SEM derived from at least 145 cells (n=2 independent experiments); *p* values ($p < 0.0001$, $p = 0.0005$) are indicated. In (b, d and f), *p* values were calculated with unpaired two-tailed t test. Source data are provided as a Source Data file.

Supplementary Fig. 4

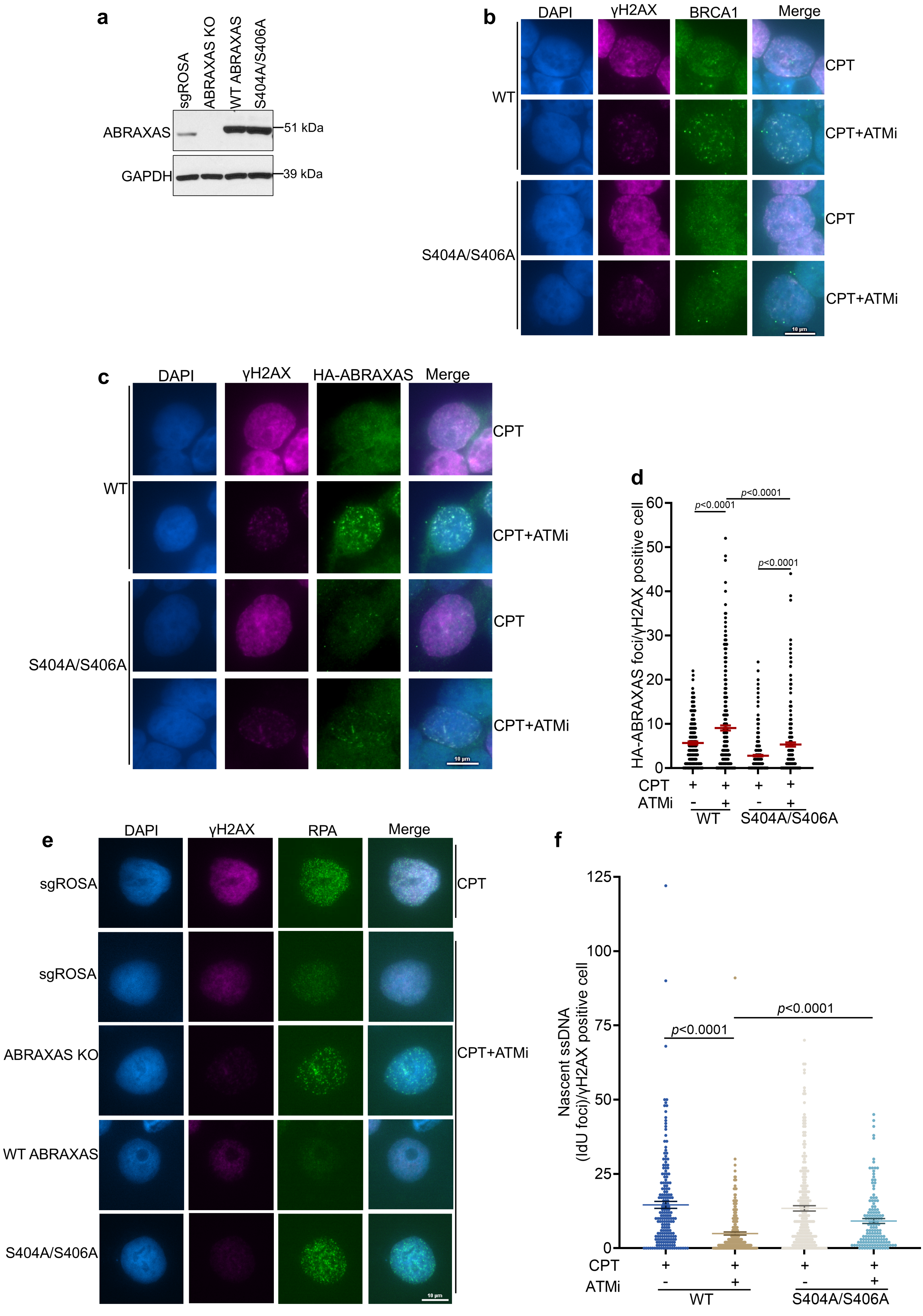

Supplementary Fig. 4. BRCA1 interaction with the A-complex suppresses end resection in ATM inhibited cells. a, Western blots showing ABRAXAS levels in ABRAXAS KO HT-29 cells complemented with WT or S404A/S406A ABRAXAS. GAPDH serves as loading control. Molecular weight markers are shown. Experiments were repeated three times with similar results. **b,** Representative immunofluorescence images of BRCA1 foci in WT and S404A/S406A ABRAXAS expressing HT-29 cells exposed to CPT (1 μM) +/- ATMi (250 nM) for 1 h. γH2AX marks cells with CPT-induced replication damage. Images were acquired with 60X magnification. Scale bar is shown. **c,** Representative immunofluorescence images of HA-ABRAXAS foci in cells described and treated as in (b). Images were acquired with 60X magnification. Scale bar is shown. **d,** Scatter plot showing quantification of the data as described in (c). Experiments were performed three times with similar results. Data represents mean ± SEM derived from at least 200 cells (n=2 independent experiments); *p* values ($p < 0.0001$, $p < 0.0001$, $p < 0.0001$) are indicated. **e,** Representative immunofluorescence images of RPA foci in control (sgROSA), ABRAXAS KO, WT and S404A/S406A ABRAXAS expressing HT-29 cells treated as described in (b). Images were acquired with 100X magnification. Scale bar is shown. **f,** Scatter plot showing quantification of nascent ssDNA (native IdU foci) in γH2AX positive WT and S404A/S406A ABRAXAS expressing HT-29 cells exposed to CPT (50 nM) alone or in combination with ATMi (250 nM) for 1 h. Data represent mean ± SEM derived from at least 142 cells (n=2 independent experiments); *p* values ($p < 0.0001$, $p < 0.0001$) are indicated. In (d and f), *p* values were calculated with unpaired two-tailed t test. Source data are provided as a Source Data file.

Supplementary Fig. 5

a

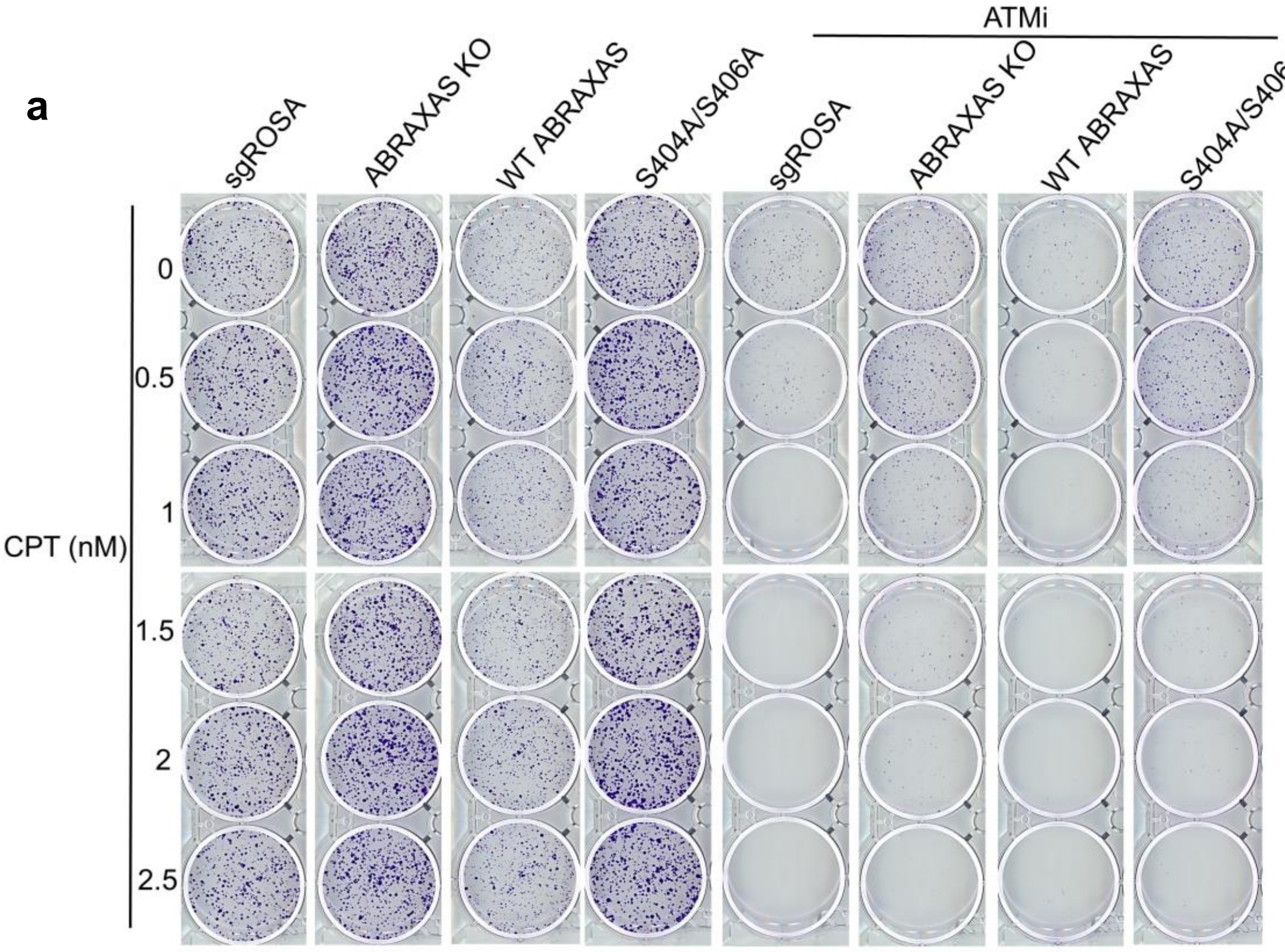

b

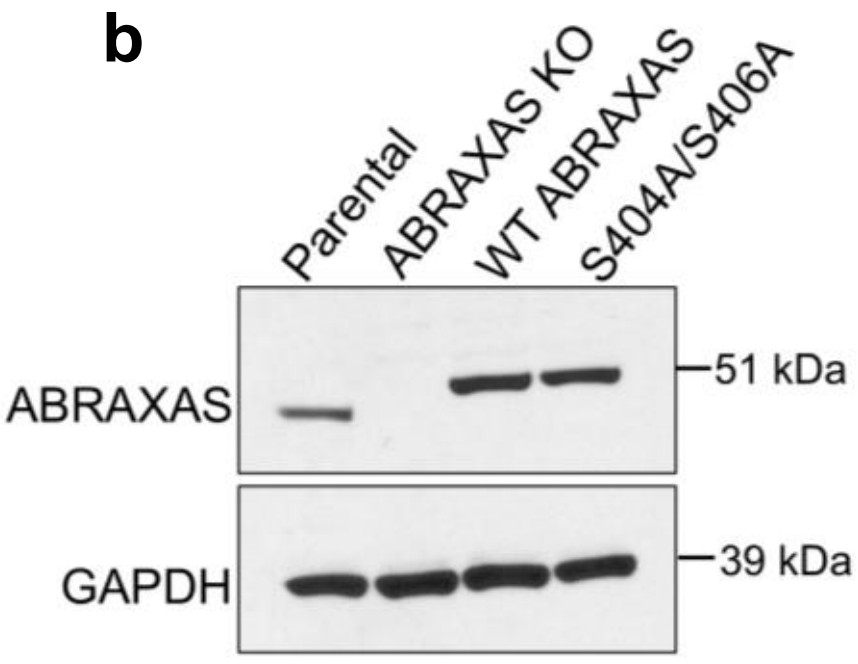

c

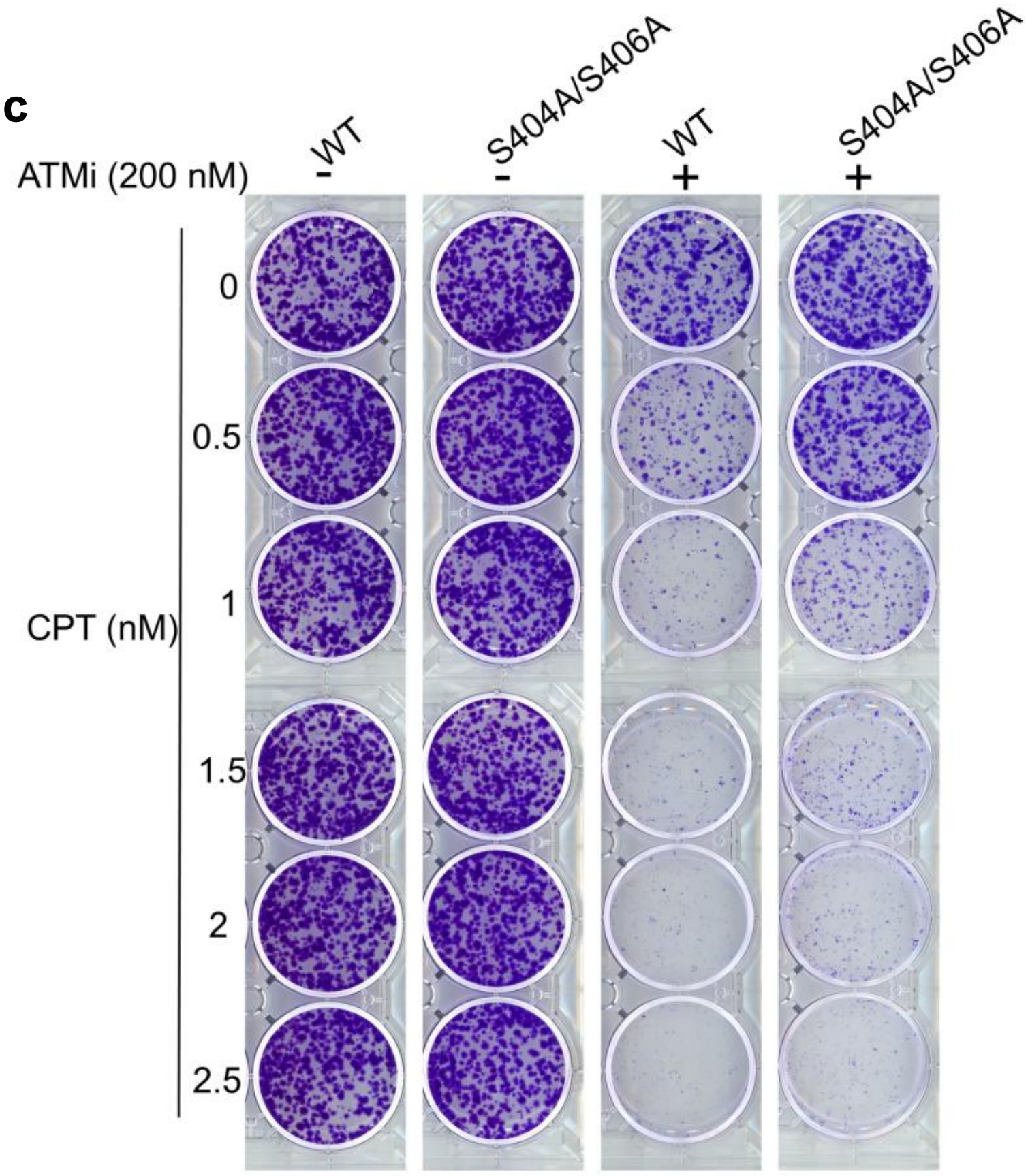

Supplementary Fig. 5. BRCA1 interaction with the A-complex suppresses cell viability upon combined CPT and ATMi treatment. a, Clonogenic survival assay showing CPT+ATMi resistance in HT-29 cells expressing ABRAXAS S404A/S406A mutant. Experiments were repeated three times with similar results. **b,** Western blots showing ABRAXAS levels in ABRAXAS KO HEK293T cells complemented with WT or S404A/S406A ABRAXAS. GAPDH serves as loading control. Molecular weight markers are shown. Experiments were repeated two times with similar results. **c,** Clonogenic survival assay showing CPT+ATMi resistance in ABRAXAS S404A/S406A expressing HEK293T cells. Experiments were repeated three times with similar results. Source data are provided as a Source Data file.

Supplementary Fig. 6

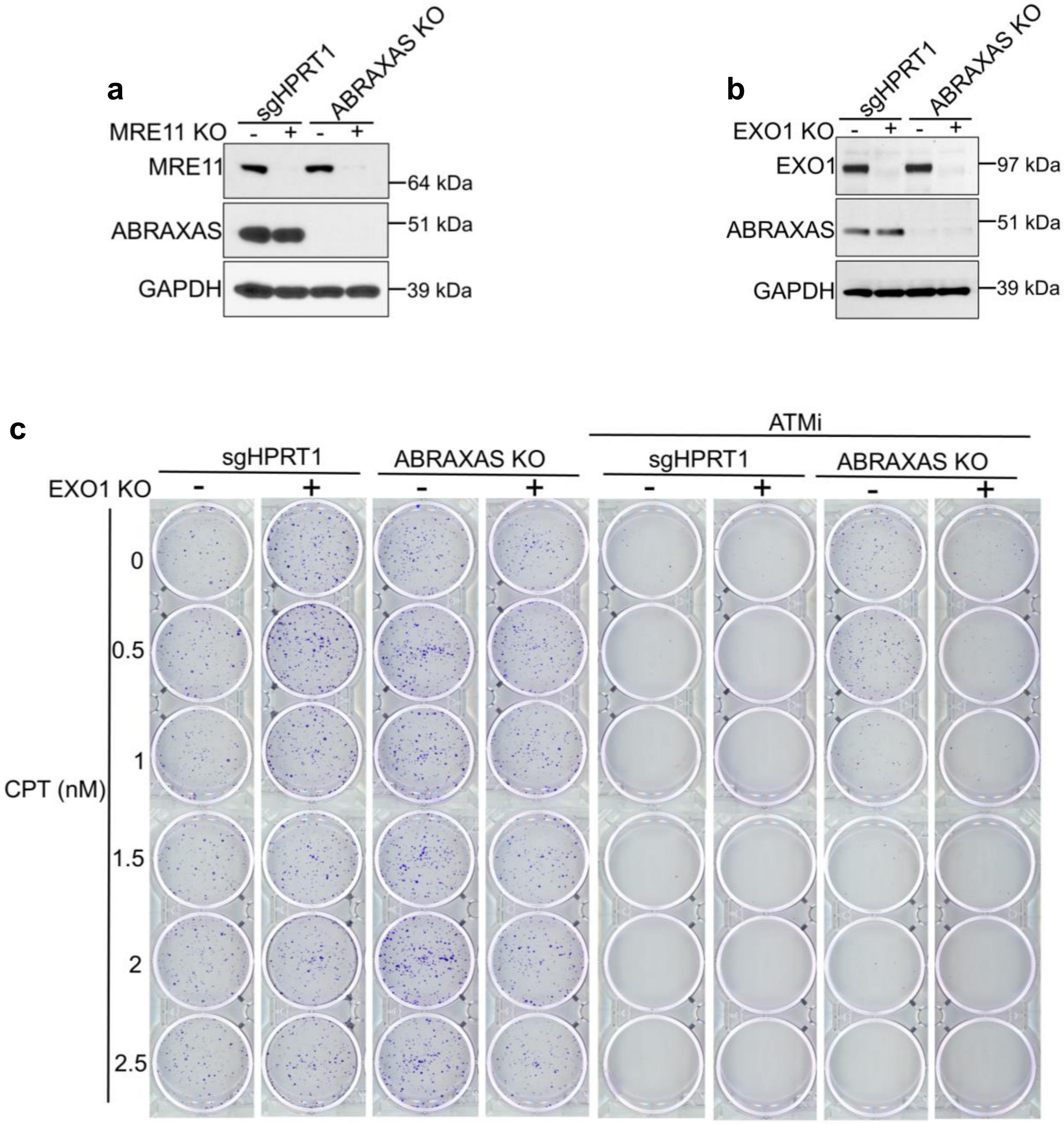

Supplementary Fig. 6. EXO1 KO re-sensitizes ABRAXAS KO cells to combined CPT and ATMi treatment. (a, b), Western blots showing MRE11 (a) or EXO1(b) KO in control (sgHPRT1) and ABRAXAS KO HT-29 cells. GAPDH serves as loading control. Molecular weight markers are shown. Experiments were repeated three times with similar results. **c,** Clonogenic survival assays showing increased CPT+ATMi sensitivity in ABRAXAS KO HT-29 cells upon EXO1 KO. Experiments were repeated n=3 times with similar results. Source data are provided as a Source Data file.

Supplementary Fig. 7

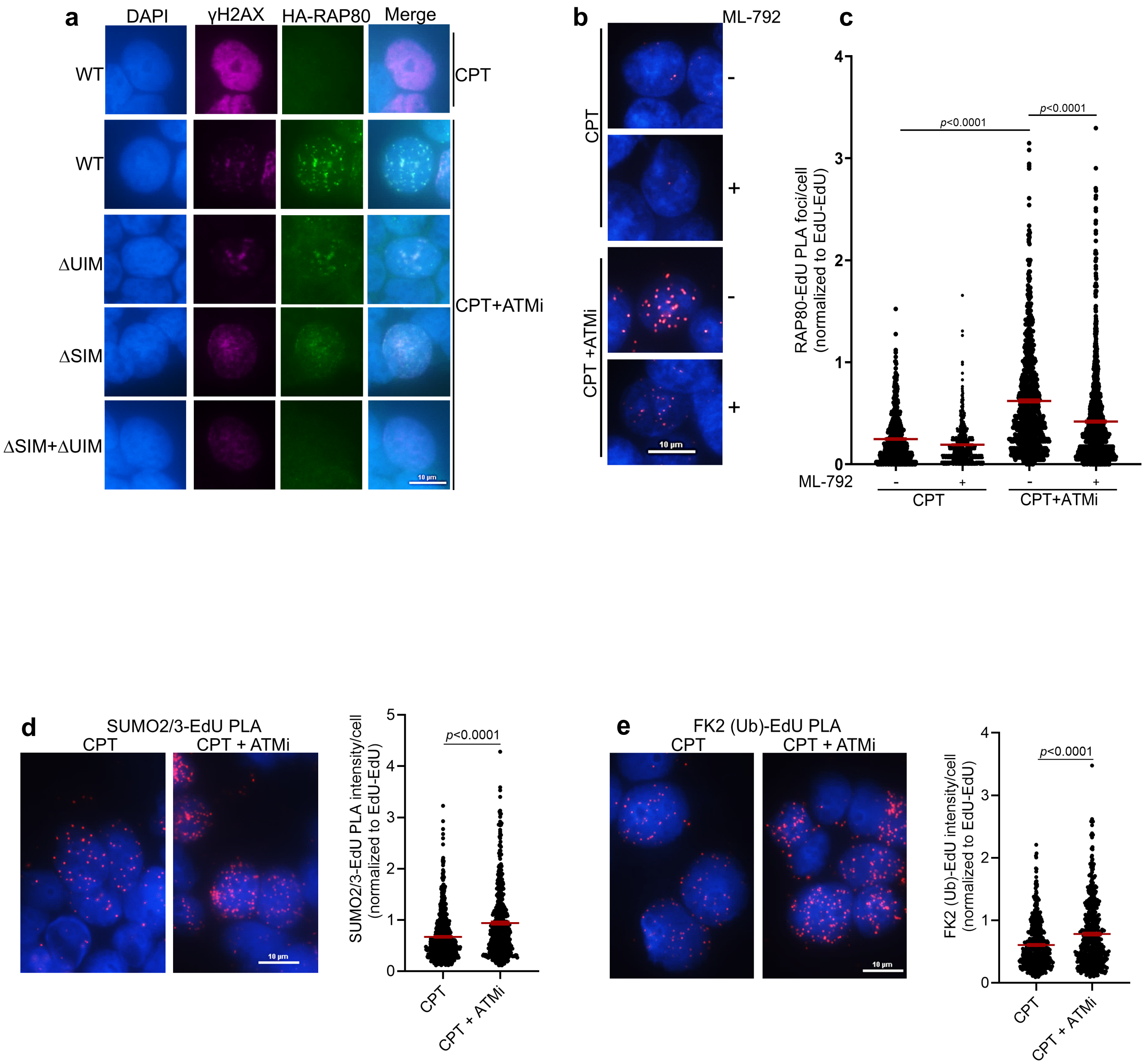

Supplementary Fig. 7. Combined SUMO and ubiquitin recognition by RAP80 facilitates BRCA1-A complex recruitment at damaged chromatin upon ATM inhibition. a, Representative immunofluorescence images of HA-RAP80 foci in WT, ΔUIM, ΔSIM or ΔSIM + ΔUIM expressing HT-29 cells treated with CPT (1 μM) +/- ATMi (250 nM) for 1 h. **(b, c)** Representative images of RAP80-EdU PLA foci in HT-29 cells treated with 50 nM CPT+/- 250 nM ATMi. Cells were incubated with ML-792 (1 μM) for 16 h prior to CPT and ATMi treatment (b). Quantification of RAP80-EdU PLA foci is shown (c). Data represent mean ± SEM derived from at least 545 cells (n=2 independent experiments); *p* values ($p < 0.0001$, $p < 0.0001$) are indicated. **d,** Left panel: Representative images of SUMO2/3-EdU PLA foci in HT-29 cells treated with 50 nM CPT+/- 250 nM ATMi as described in (b). Right panel: Scatter plot showing quantification of SUMO2/3-EdU SIRF foci normalized to average EdU-EdU signal in each experimental condition. Experiments were performed three times with similar results. Data represent mean ± SEM derived from at least 500 cells (n=2 independent experiments); *p* values ($p < 0.0001$) are indicated. **e,** Left panel: SIRF experiment showing FK2-Ub accumulation at CPT-damaged fork upon ATM inhibition. HT-29 cells were treated as described in (d). Right panel: Quantification of the data is shown. Experiments were performed four times with similar results. Data represent mean ± SEM derived from at least 506 cells (n=2 independent experiments); *p* values ($p < 0.0001$) are indicated. In (a, b, d and e), images were acquired with 60X magnification. Scale bar is shown. In (c, d and e), *p* values were calculated with unpaired two-tailed t test. Source data are provided as a Source Data file.

Supplementary Fig. 8

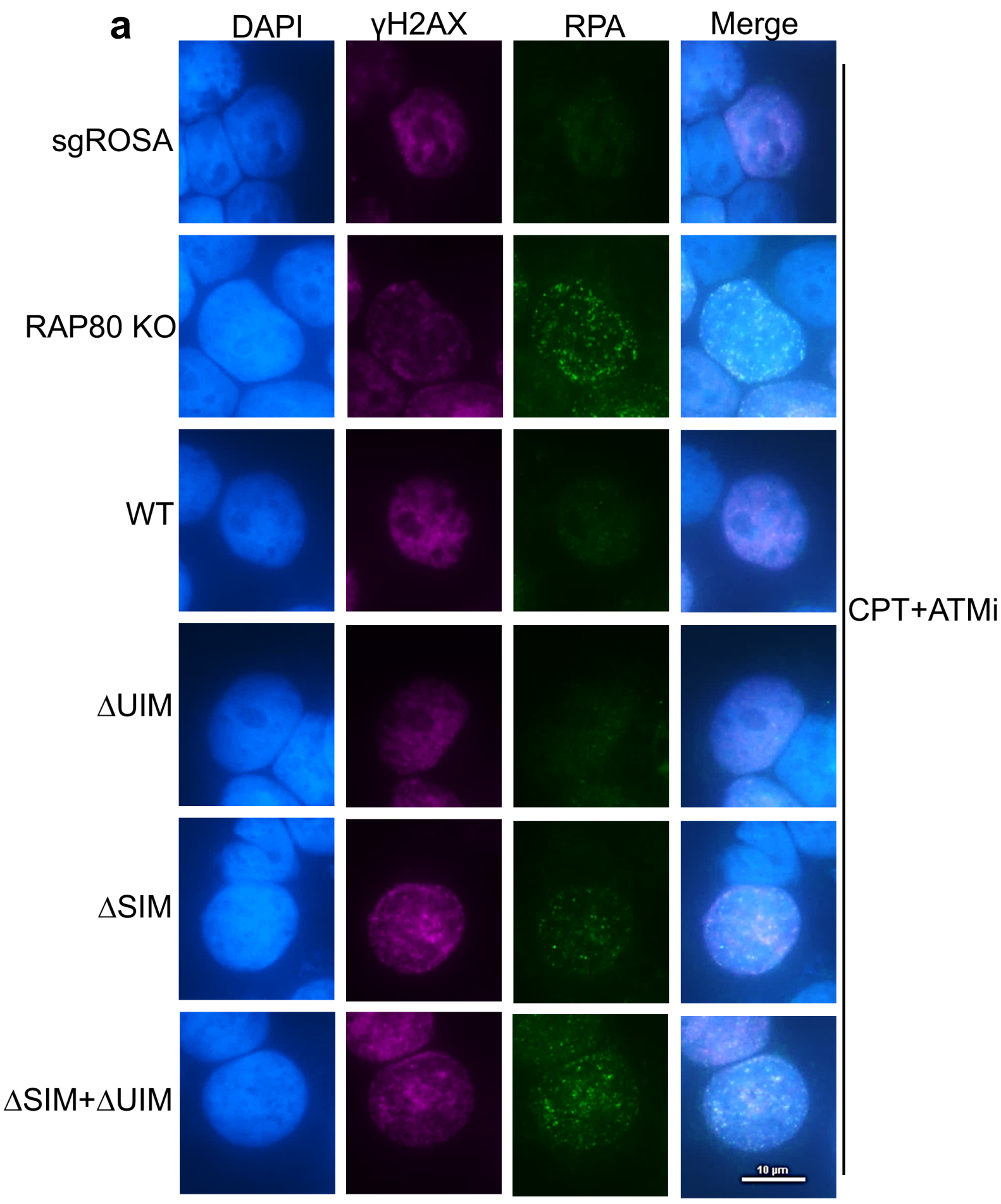

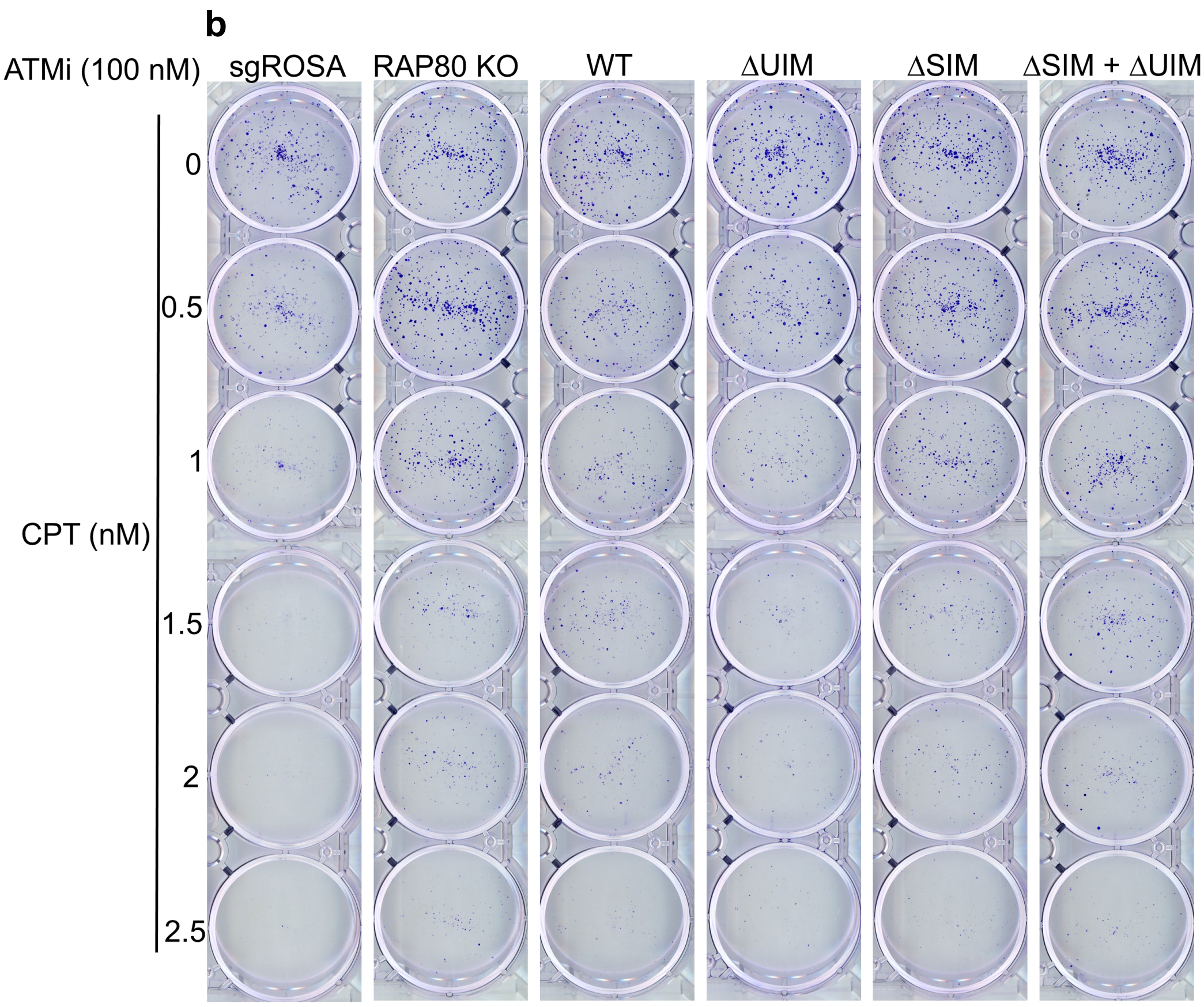

Supplementary Fig. 8. Combined SUMO and ubiquitin recognition by RAP80 suppresses end resection and viability in ATM-inhibited cells. a, Representative RPA immunofluorescence in control (sgROSA) and RAP80 KO HT-29 cells complemented with WT or RAP80 deletion mutants. Cells were pretreated with ATMi (250 nM) for 10 min followed by CPT (1 μM) + ATMi (250 nM) treatment for 1 h. Images were acquired with 60X magnification. Scale bar is shown. **b,** Representative colony images of control (sgROSA) or RAP80 KO HT-29 cells harboring empty vector (EV) or complemented with WT or RAP80 deletion mutants, Cells were treated with 100 nM ATMi in combination with the indicated doses of CPT. Experiments were repeated three times with similar results.

Supplementary Fig. 9

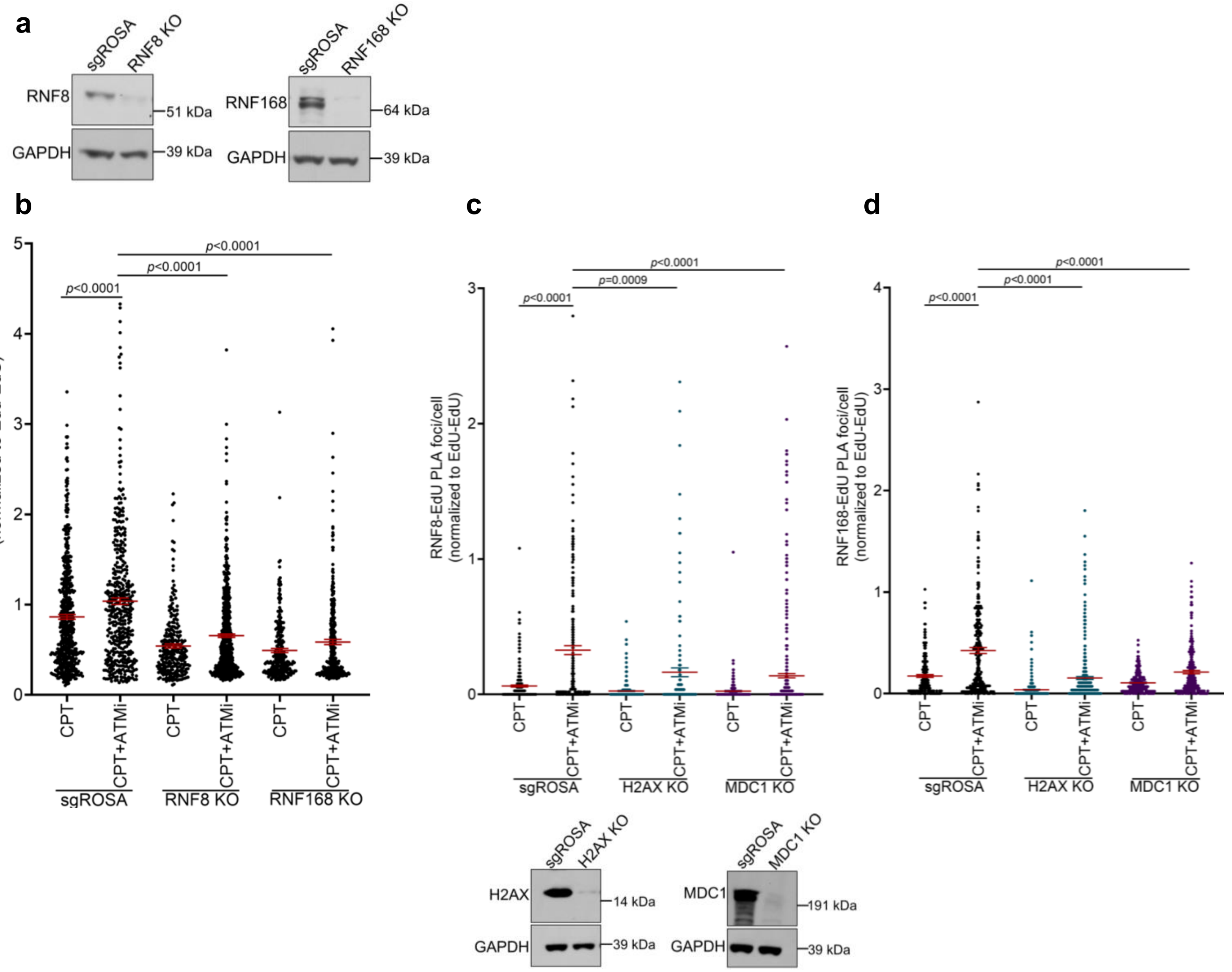

Supplementary Fig. 9. ATM inhibition increases RNF8 and RNF168 dependent ubiquitylation of damaged replication forks. a, Western blots showing RNF8 or RNF168 KO in HT-29 cells. GAPDH serves as loading control. Molecular weight markers are shown. Western blotting was repeated three times with similar results. **b,** Scatter plot showing quantification of FK2-EdU PLA signal normalized to average EdU-EdU signal in RNF8 or RNF168 KO HT-29 cells treated with 50 nM CPT+/- 250 nM ATMi. Cells were optionally pretreated with ATMi for 10 minutes followed by CPT treatment for 40 minutes. EdU was added during the last 10 min of CPT treatment. ATMi was present throughout the experiment. Data represent mean ± SEM derived from at least 261 cells (n=2 independent experiments); p values ($p < 0.0001$, $p < 0.0001$, $p < 0.0001$) are indicated. **(c, d)** Scatter plot showing quantification of RNF8-EdU (c) and RNF168-EdU (d) PLA foci normalized to average EdU-EdU PLA signal in H2AX or MDC1 KO HT-29 cells treated as in (b). Data represent mean ± SEM derived from at least 144 (c) and 182 (d) cells (n=2 independent experiments); p values (c, $p < 0.0001$, $p = 0.0009$, $p < 0.0001$; d, $p < 0.0001$, $p < 0.0001$, $p < 0.0001$) are indicated. H2AX and MDC1 KO in HT-29 cells were verified by Western blotting. GAPDH serves as loading control. Molecular weight markers are shown. Western blotting was repeated three times with similar results. In (b, c and d), p values were calculated with unpaired two-tailed t test. Source data are provided as a Source Data file.

Supplementary Fig. 10

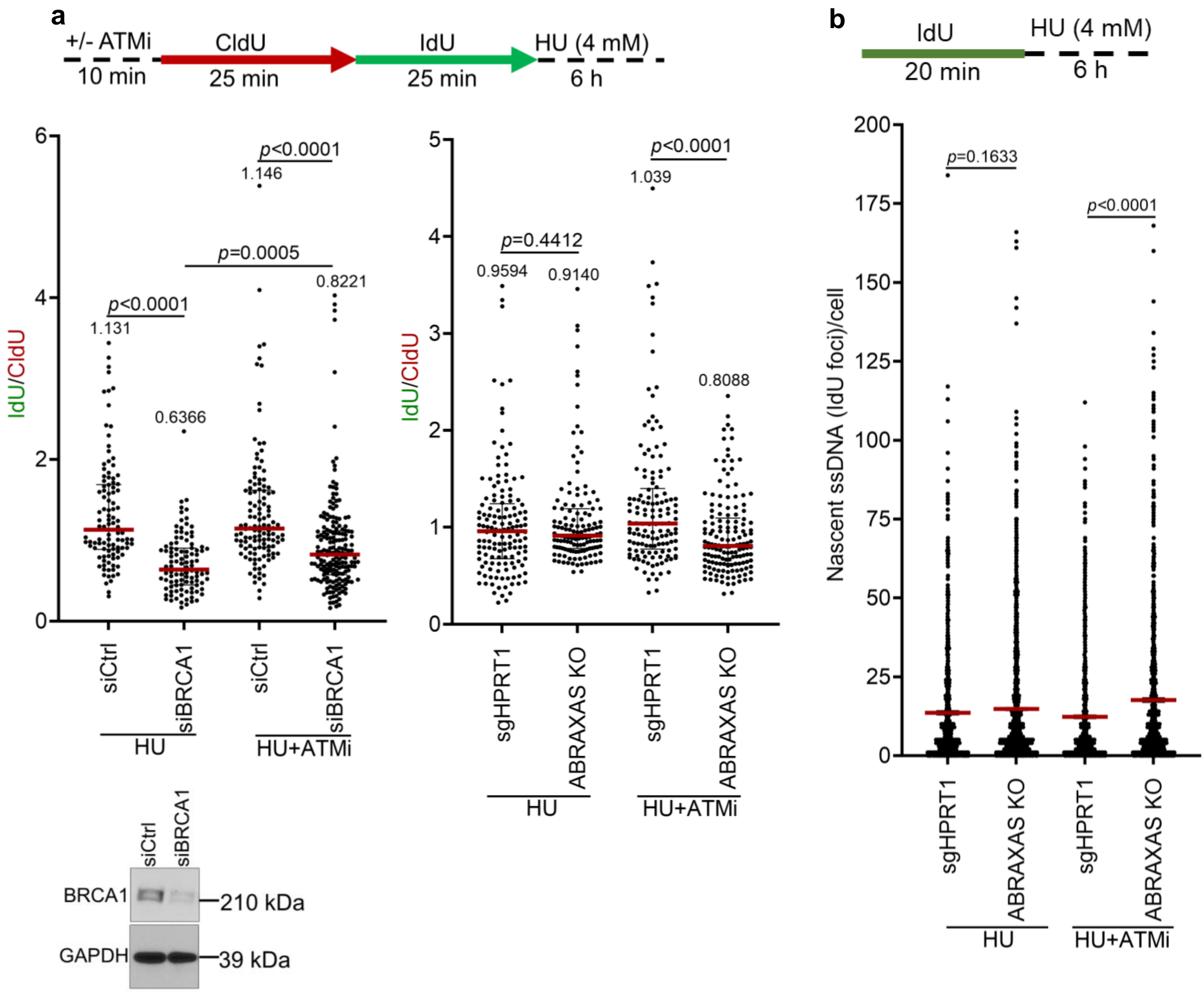

Supplementary Fig. 10. BRCA1-A complex loss causes controlled resection of HU-stalled forks in ATM-inhibited cells. a, Schematic showing the strategy of fork degradation assay using DNA fiber technique in BRCA1 depleted (left panel) or ABRAXAS KO (right panel) HT-29 cells treated with HU +/- ATMi as indicated. For BRCA1 depletion, cells were transfected with 100 nM of control or BRCA1 siRNA. Seventy-two h post transfection, cells were subjected to DNA fiber assay. Scatter plot showing quantification of the data. Data represent median IdU/CldU with interquartile range. Median IdU/CldU values are indicated. *p* values (left panel, $p < 0.0001$, $p = 0.0005$, $p < 0.0001$; right panel, $p = 0.4412$, $p < 0.0001$) were derived from at least 100 DNA fibers (n=2 independent experiments) using two-tailed Mann–Whitney test. BRCA1 knockdown was verified by Western blotting. GAPDH serves as loading control. GAPDH derived from the same experiment was run on a different gel. Molecular weight markers are shown. Western blotting was repeated two times with similar results. **b,** Schematic showing the strategy of native IdU assay with HU. Scatter dot plot showing quantification of native IdU experiment results in control (sgHPRT1) and ABRAXAS KO HT-29 cells treated with HU (4 mM) +/-ATMi (250 nM) as indicated. Data represent mean ± SEM derived from at least 829 cells (n=2 independent experiments); *p* values ($p = 0.1633$, $p < 0.0001$) are indicated, unpaired two-tailed t test. Source data are provided as a Source Data file.

Supplementary Fig. 11

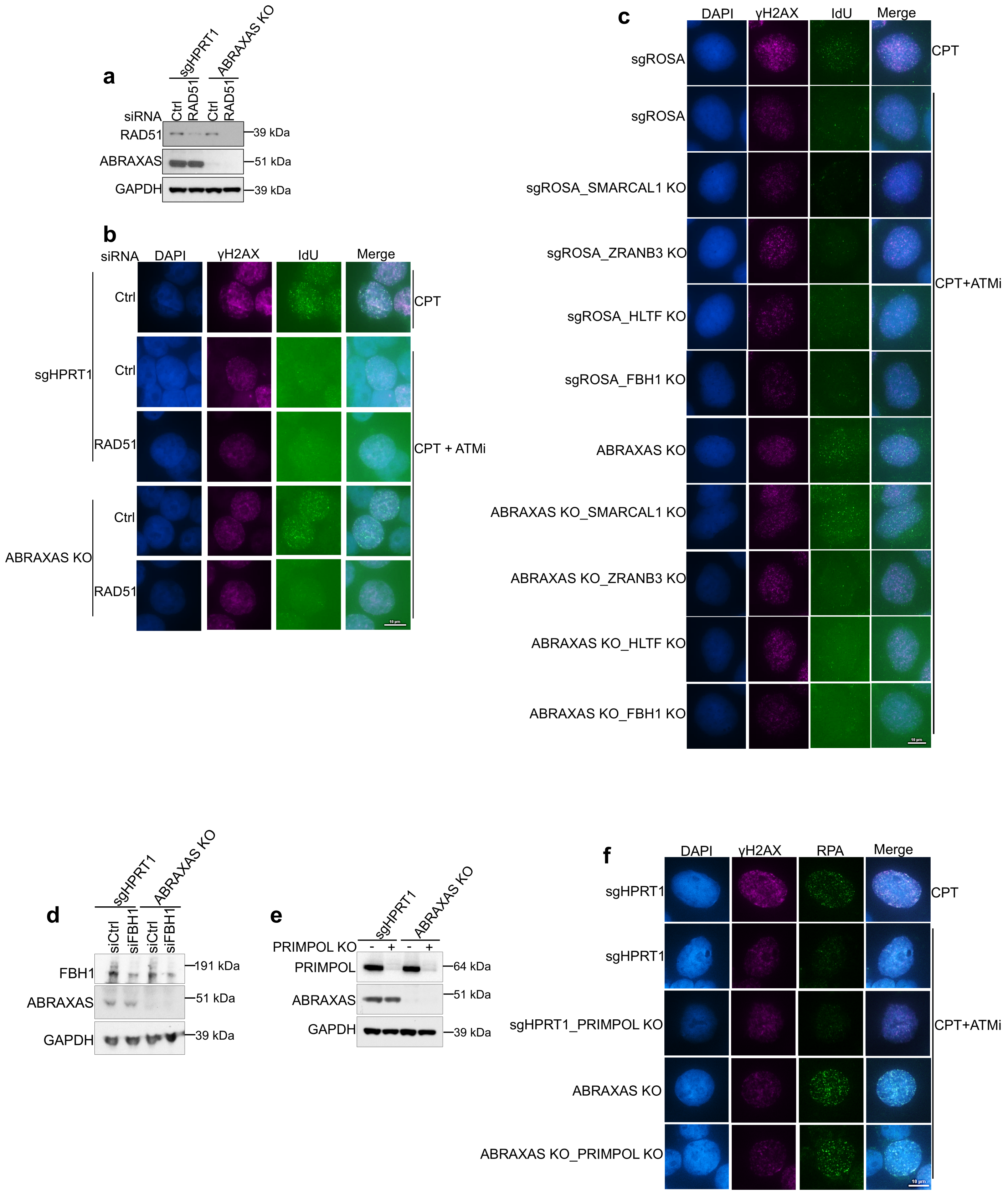

Supplementary Fig. 11. Reversed forks but not ssDNA gaps are resection substrates in BRCA1-A deficient cells. a, Western blots showing RAD51 levels in control (sgHPRT1) and ABRAXAS KO HT-29 cells transfected with 100 nM of control or RAD51 siRNA. GAPDH serves as loading control. Molecular weight markers are shown. Western blotting was repeated two times with similar results. **b**, Representative images of IdU immunofluorescence detected under non-denaturing conditions in control and ABRAXAS KO HT-29 cells upon siRNA-mediated depletion of RAD51. Experiment was performed 72 h post transfection. Cells were treated with 50 nM CPT +/- 250 nM ATMi for 1 h before processed for immunofluorescence. Images were acquired with 60X magnification. Scale bar is shown. **c,** Representative images of IdU immunofluorescence representing ssDNA at reversed forks in U2OS control (sgROSA) and ABRAXAS KO cells upon SMARCAL1, ZRANB3, HLTF or FBH1 KO. Cells were treated with 50 nM CPT for 60 minutes. IdU was added during the last 40 minutes of CPT treatment. Cells were optionally pre-treated with ATMi (250 nM) 10 minutes before CPT addition and continued throughout the experiment. Images were acquired with 60X magnification. Scale bar is shown. **d,** Western blots showing FBH1 levels in control (sgHPRT1) and ABRAXAS KO HT-29 cells transfected with 100 nM of control or FBH1 siRNA. GAPDH serves as a loading control. Molecular weight markers are shown. Western blotting was repeated two times with similar results. **e,** Western blots showing PRIMPOL KO in control (sgHPRT1) and ABRAXAS KO HT-29 cells. GAPDH serves as a loading control. Molecular weight markers are shown. Western blotting was repeated three times with similar results. **f,** Representative RPA immunofluorescence in control (sgHPRT1) and ABRAXAS KO HT-29 cells upon PRIMPOL KO. Cells were treated with 50 nM CPT +/- ATMi (250 nM) as indicated for 1 h. Images were acquired with 60X magnification. Scale bar is shown. Source data are provided as a Source Data file.

Supplementary Fig. 12

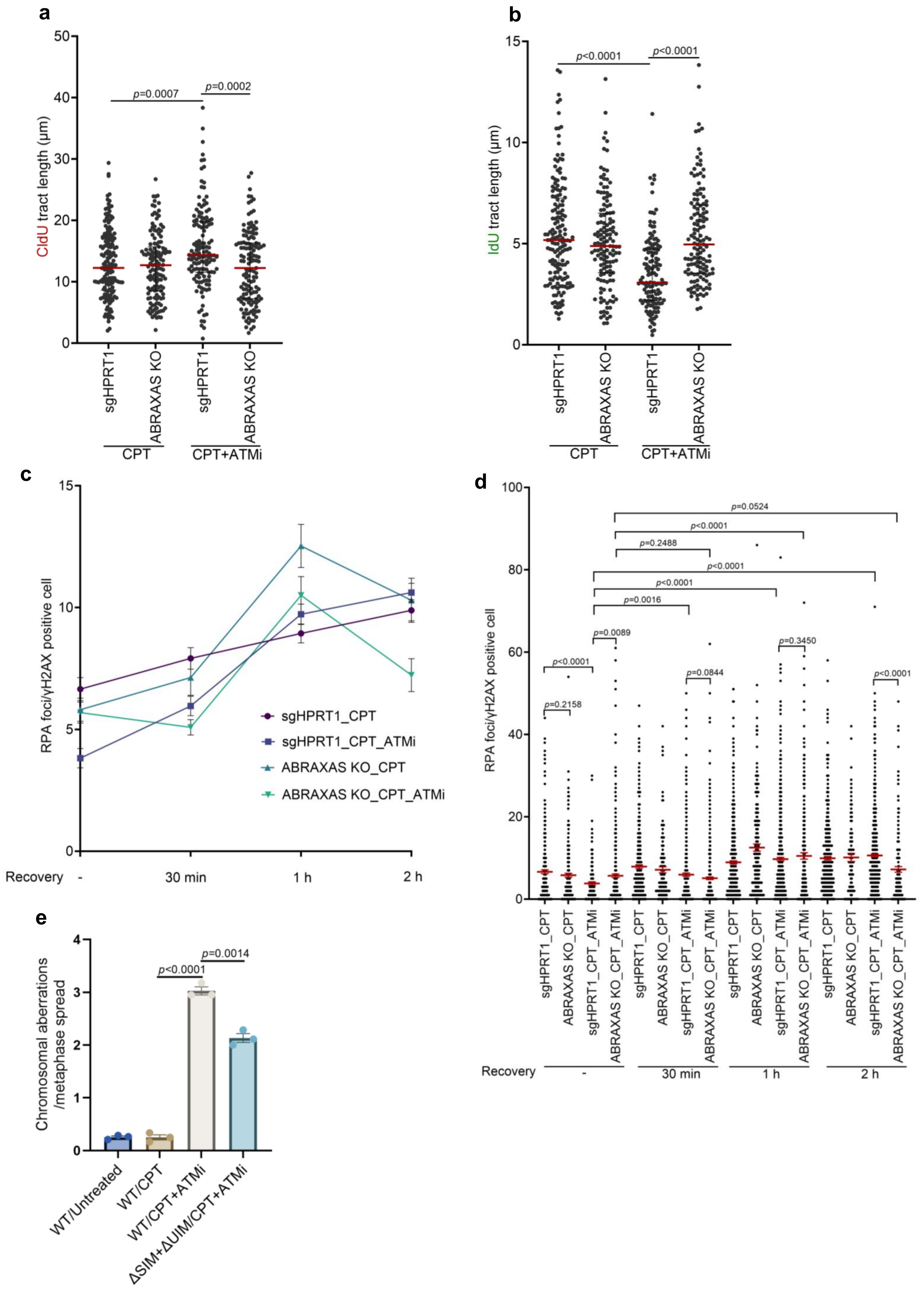

Supplementary Fig. 12. BRCA1-A complex loss rescues fork restart and chromosomal stability in ATMi-treated cells. (a, b) Scatter plot showing CldU (a) and IdU (b) tract length determined from the fork restart experiments in HT-29 cells as described in Fig. 7a. *p*-values (a, $p = 0.0007$, $p = 0.0002$; b, $p < 0.0001$, $p < 0.0001$) were determined from at least 135 DNA fibers (n=3 independent experiments) using two-tailed Mann–Whitney test. Data represent median CldU (a) and IdU (b) tract length with interquartile range. **c,** Line graph showing RPA foci following recovery from 1 h treatment with CPT (50 nM) +/- ATMi (250 nM) in control (sgHPRT1) and ABRAXAS KO HT-29 cells. Data represent mean ± SEM of n=3 independent experiments. **d.** Scatter plot showing distribution of RPA foci in experiment as described in (c). Data represent mean ± SEM derived from at least 129 γH2AX positive cells (n=3 independent experiments); *p* values ($p = 0.2158$, $p < 0.0001$, $p = 0.0089$, $p = 0.0016$, $p < 0.0001$, $p < 0.0001$, $p = 0.2488$, $p < 0.0001$, $p = 0.0524$, $p = 0.0844$, $p = 0.3450$, $p < 0.0001$) are indicated, unpaired two-tailed t test. **e,** Bar graph showing quantification of chromosomal aberrations in HT-29 cells expressing either WT or SIM+UIM deleted RAP80. Data represent mean ± SEM of n=3 independent experiments. At least 60 metaphase spreads were analyzed for each condition across three repeats; *p* values ($p < 0.0001$, $p = 0.0014$) are indicated; unpaired two-tailed t test. Source data are provided as a Source Data file.

**Supplementary Table 1.** List of sgRNAs	
**sgRNAs for spCas9**	**Sequence**
sgROSA	GAAGATGGGCGGGAGTCTTC
sgMRE11#1	GTTTGCTGCGTATTAAAGGG
sgEXO1#2	GCGTGGGATTGGATTAGCAA
sgABRAXAS	GGCGGCGGTAGCATGGA
sgRAP80#1	ATTGTGATATCCGATAGTGA
sgATM#1	TTGGTTACATACTTGGACT
sgPRIMPOL#2	GGAGCTGAAATATTAAATGC
sgRNF8#4	GAGGCTCAAAATACAGATGC
sgRNF168#1	GATACCCGGCGGCGACAGAA
sgH2AX#1	GCTCCAGGATCTCAGCGGTG
sgMDC1#1	GGTGTAACGTGGAGCCAGTA
**sgRNAs for saCas9**	**Sequence**
sgHPRT1	GTCCCAACAGCAATTCCTAAA
sgABRAXAS#1	ATATAAGGAATGTTCCAGTCG

**Supplementary Table 2.** List of antibodies			
**Antibodies for Western blotting**	**Source**	**Identifier**	**Dilution**
Mouse PCNA(PC10)	Cell Signaling Technology	Cat# 2586	1:1000
Rabbit Histone H3	Cell Signaling Technology	Cat# 9715	1:1000
Mouse anti-BrdU(B44)	BD biosciences	Cat# 347580	1:200
Mouse MRE11	GeneTex	Cat# GTX70212	1:1000
Rabbit EXO1	GeneTex	Cat# GTX109891	1:1000
Rabbit ABRAXAS	Fortis Life Sciences	Cat# A302-180A	1:1000
Rabbit RAP80 (D1T6Q)	Cell Signaling Technology	Cat# 14466	1:1000
Mouse RAD51 [14B4]	GeneTex	Cat# GTX70230	1:1000
Rabbit anti-Phospho KAP-1 (S824)	Fortis Life Sciences	Cat# A300-767A	1:1000
Mouse γH2AX (Ser139), clone JBW301	Millipore Sigmma	Cat# 05-636	1:1000
Rabbit GAPDH (14C10)	Cell Signaling Technology	Cat# 2118	1:5000
Rabbit PRIMPOL	Proteintech	Cat# 29824-1-AP	1:1000
Rabbit ATM (D2E2)	Cell Signaling Technology	Cat#2873	1:1000
Mouse RNF8 (B-2)	Santa Cruz Biotechnology	sc-271462	1:1000
Rabbit RNF168	Millipore Sigma	ABE367	1:1000
Rabbit H2AX	Fortis Life Sciences	A300-083A	1:1000
Rabbit MDC1	Abcam	ab11169	1:1000
Mouse FBH1 (2353C1a)	Abcam	ab58881	1:250
HRP-conjugated anti-mouse secondary antibody	Amersham	Cat# NA931	1:3000
HRP-conjugated anti-rabbit secondary antibody	Amersham	Cat# NA934	1:3000
**Antibodies for Immunofluorescence**	**Source**	**Identifier**	
Mouse RPA, clone RPA34-20	Millipore Sigma	Cat# MABE285	1:1000
Rat Rpa32/rpa2 (4E4)	Cell Signaling Technology	Cat #2208S	1:1000
Rabbit γH2AX (Ser139) (20E3)	Cell Signaling Technology	Cat #9718	1:1000
Rabbit γH2AX (Ser139)	Active Motif	Cat# 39117	1:1000
Mouse γH2AX (Ser139), clone JBW301	Millipore Sigma	Cat# 05-636	1:1000
Mouse MRE11	GeneTex	Cat# GTX70212	1:100
Rabbit EXO1	GeneTex	Cat# GTX109891	1:200
Rabbit RAP80 (D1T6Q)	Cell Signaling Technology	Cat# 14466	1:200
Mouse BRCA1 (D-9)	Santa Cruz Biotechnology	Cat# sc-6954	1:100
Mouse anti-HA.11 Epitope Tag	BioLegend	Cat# 901516	1:500
Mouse anti-Ubiquitin (FK2)	Millipore Sigma	Cat#04-263	1:100
Mouse Sumo 2 + Sumo 3 [8A2]	Abcam	Cat# ab81371	1:100
Rabbit anti-biotin (D5A7)	Cell Signaling Technology	Cat# 5597	1:200
Mouse anti-biotin	Millipore Sigma	Cat# SAB4200680	1:100
Mouse RNF8 (B-2)	Santa Cruz Biotechnology	sc-271462	1:100
Rabbit RNF168	Millipore Sigma	ABE367	1:200
Rat anti-CIdU	Abcam	Cat# ab6326	1:200
Mouse anti-BrdU (B44)	BD Pharmigen	Cat# 47580	1:40
AlexaFluor 488-conjugated anti-mouse secondary antibody	Thermo Scientific	Cat# A-11029	1:500
AlexaFluor 647-conjugated anti-rat secondary antibody	Thermo Scientific	Cat# A-21247	1:500
AlexaFluor 647-conjugated anti-rabbit secondary antibody	Thermo Scientific	Cat# A-21245	1:500

Uncropped blots of Supplementary Fig 1a.

Dotted outline denotes samples included in the manuscript.

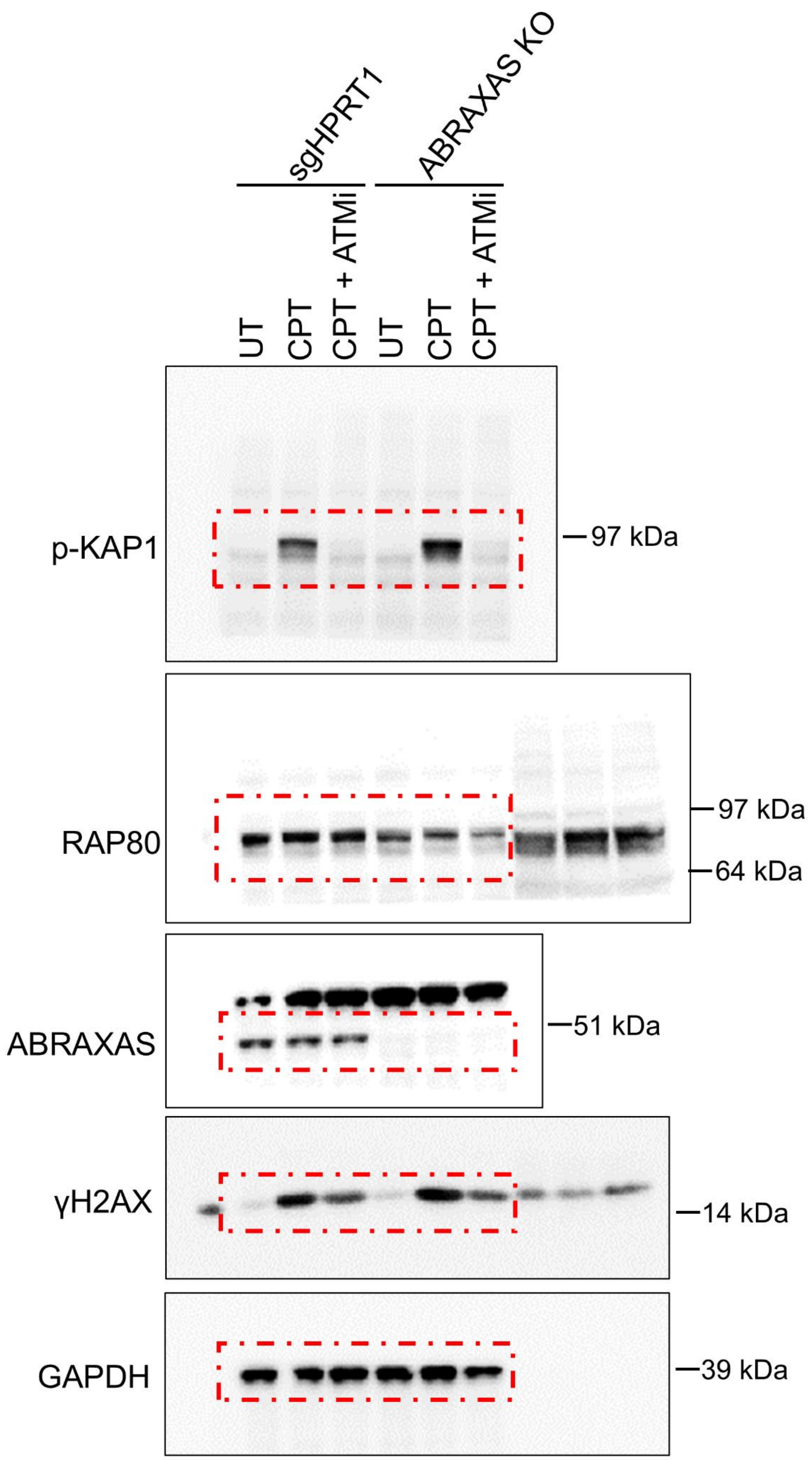

Uncropped blots of Supplementary Fig 1c.

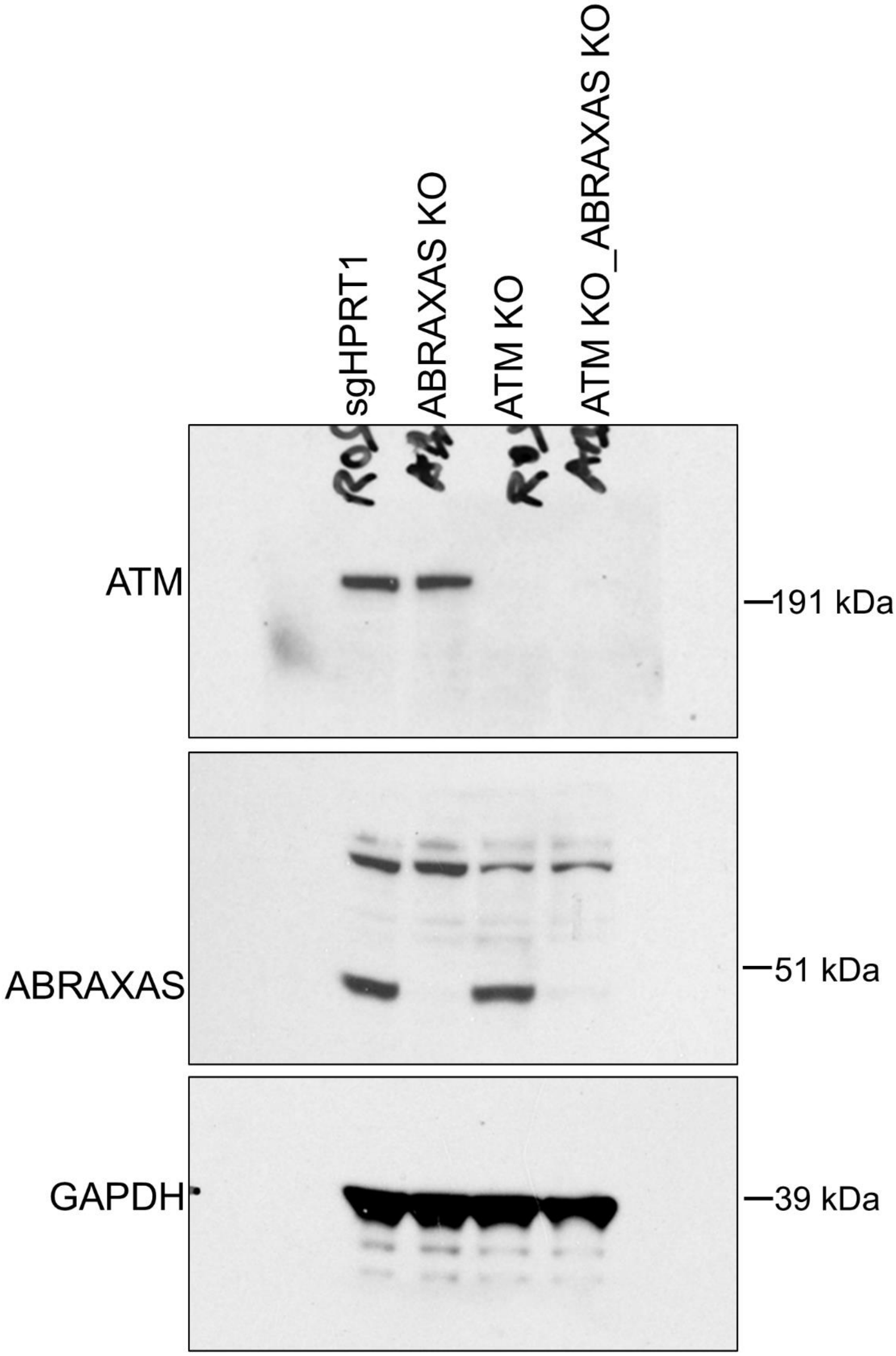

Uncropped blots of Supplementary Fig. 4a.

Dotted outline denotes samples included in the manuscript.

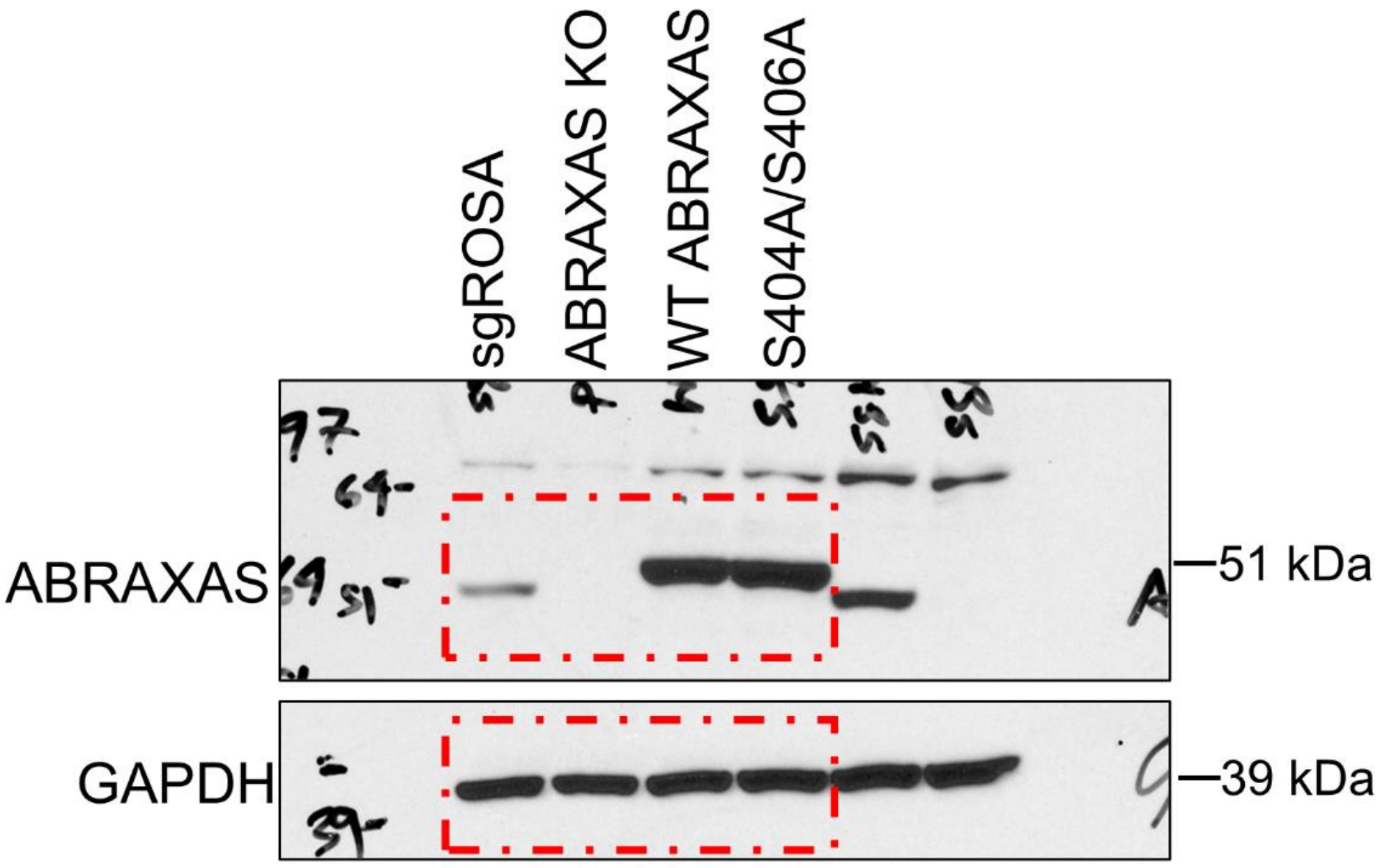

Uncropped blots of Supplementary Fig. 5b

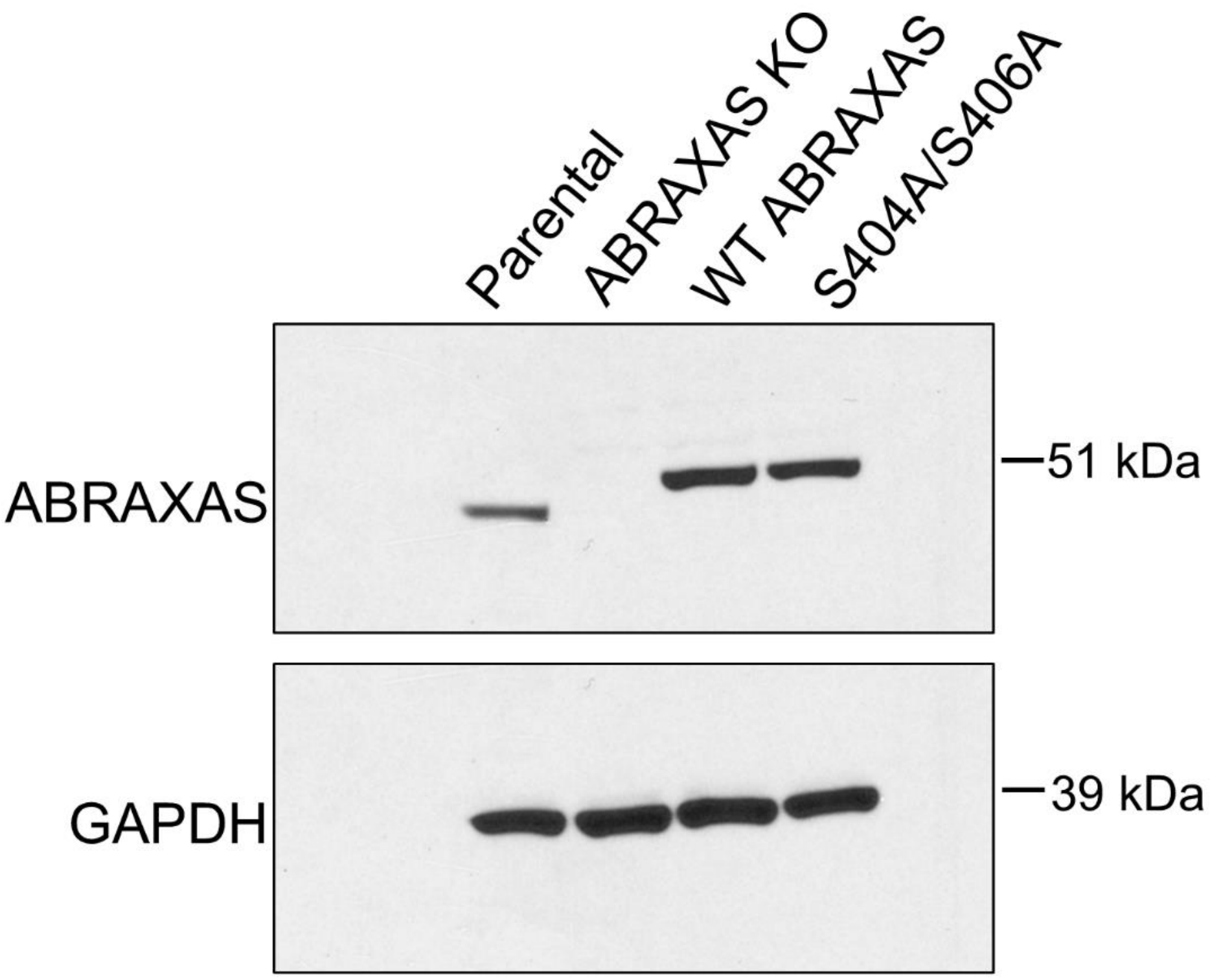

Uncropped blots of Supplementary Fig. 6a.

Dotted outline denotes samples included in the manuscript.

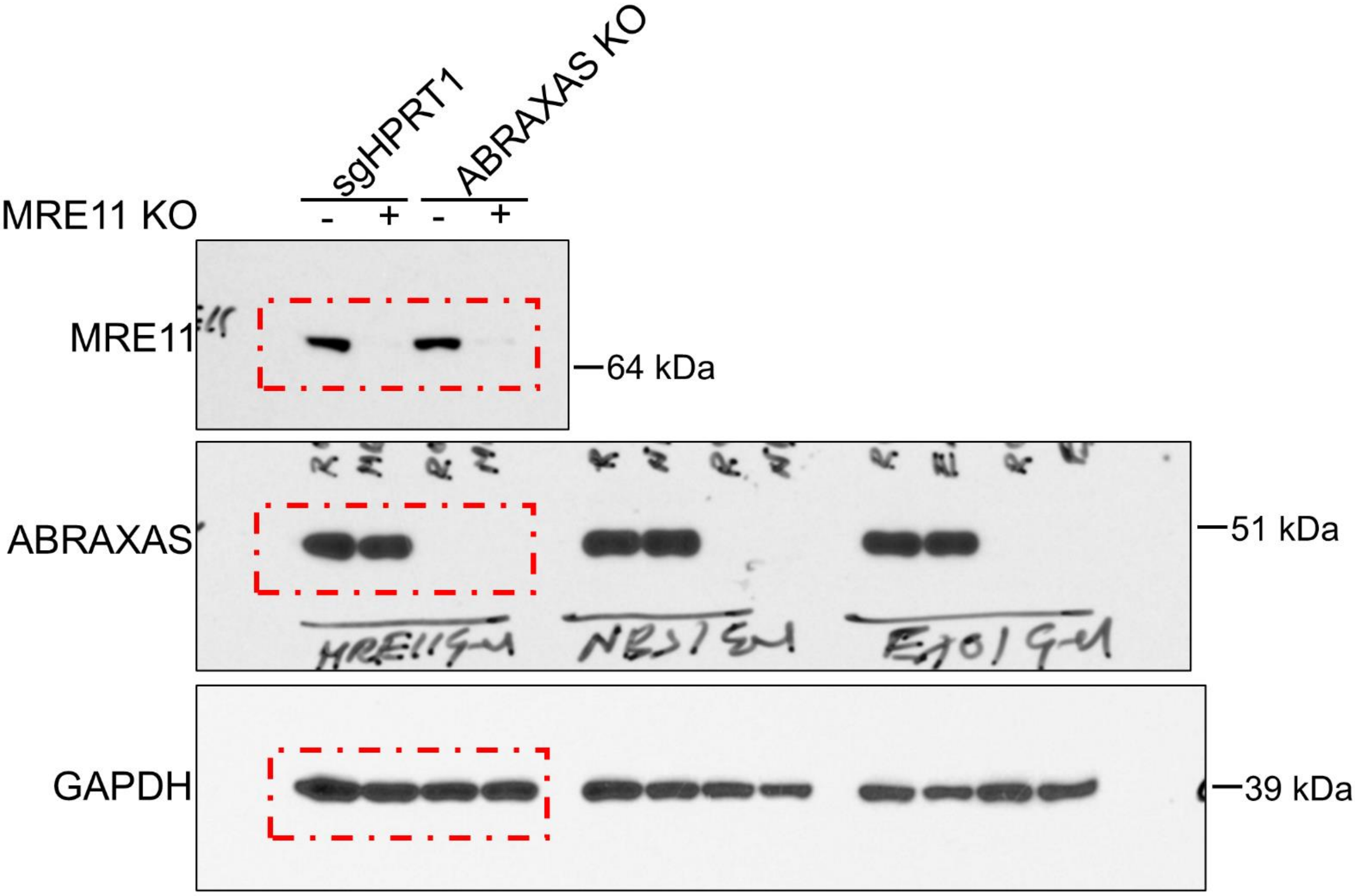

Uncropped blots of Supplementary Fig. 6b.

Dotted outline denotes samples included in the manuscript.

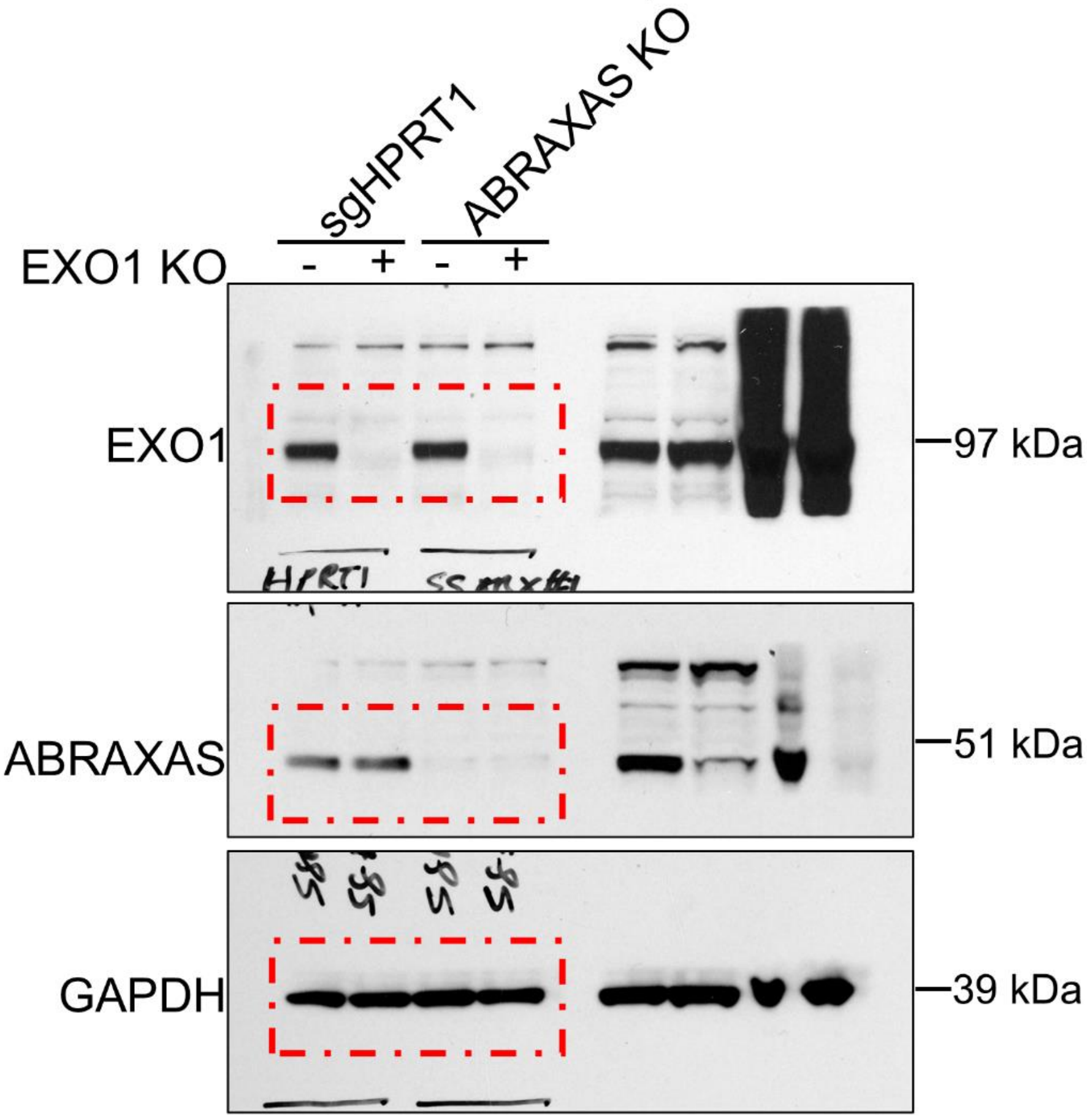

Uncropped blots of Supplementary Fig. 9a.
Dotted outline denotes samples included in the manuscript.

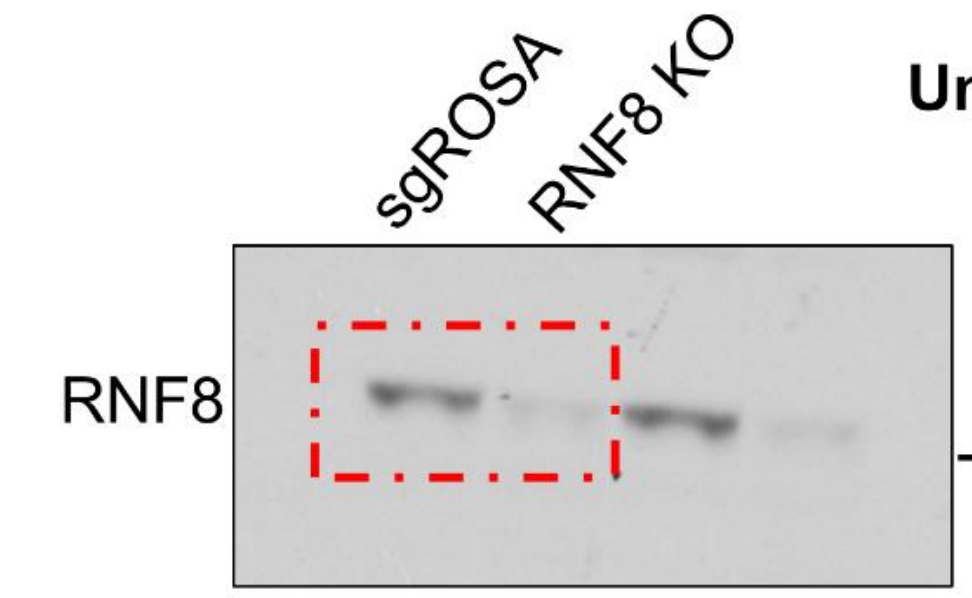

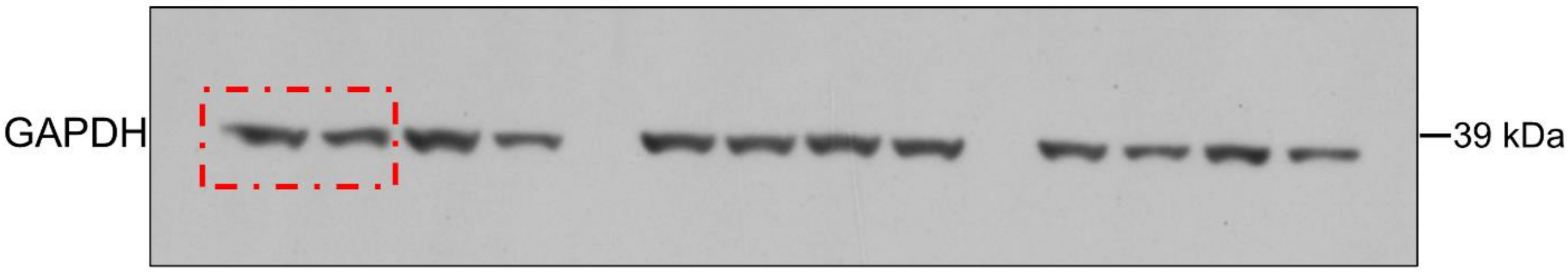

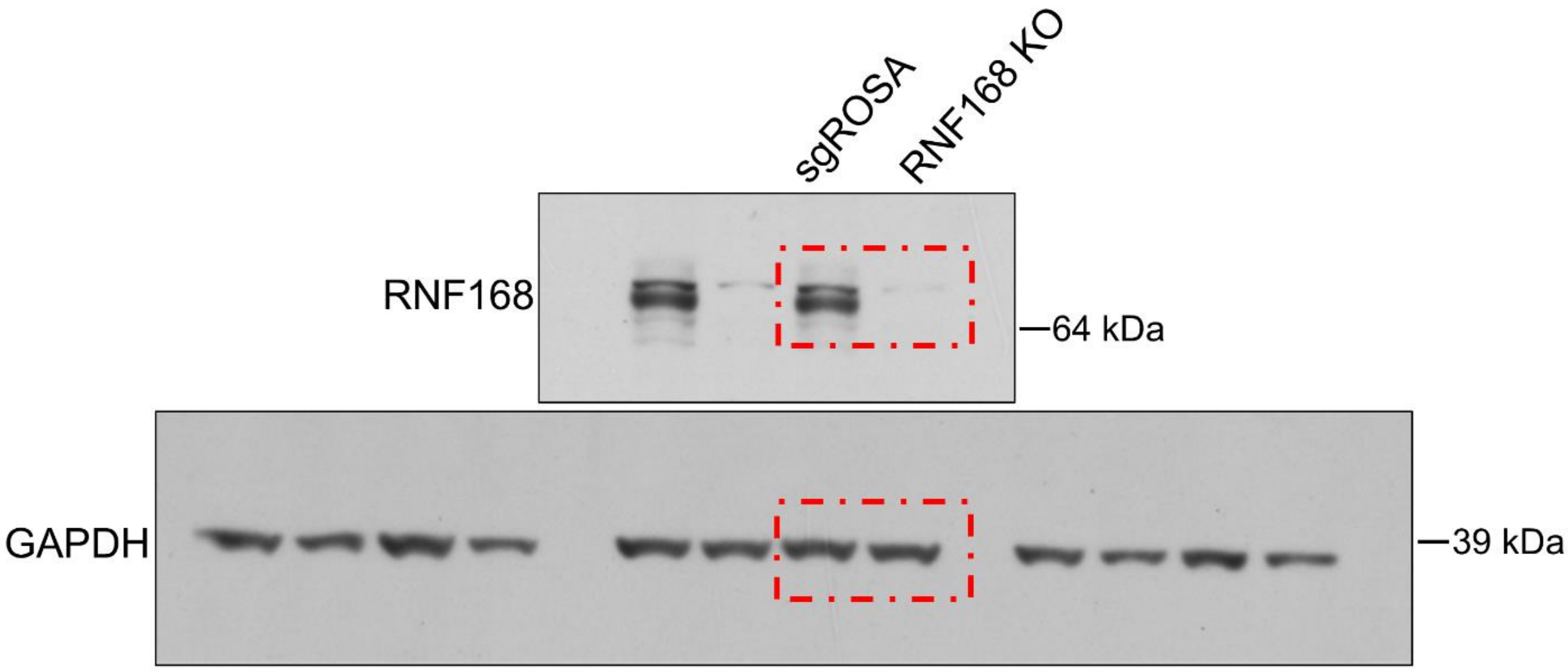

Uncropped blots of Supplementary Fig. 9c.
Dotted outline denotes samples included in the manuscript.

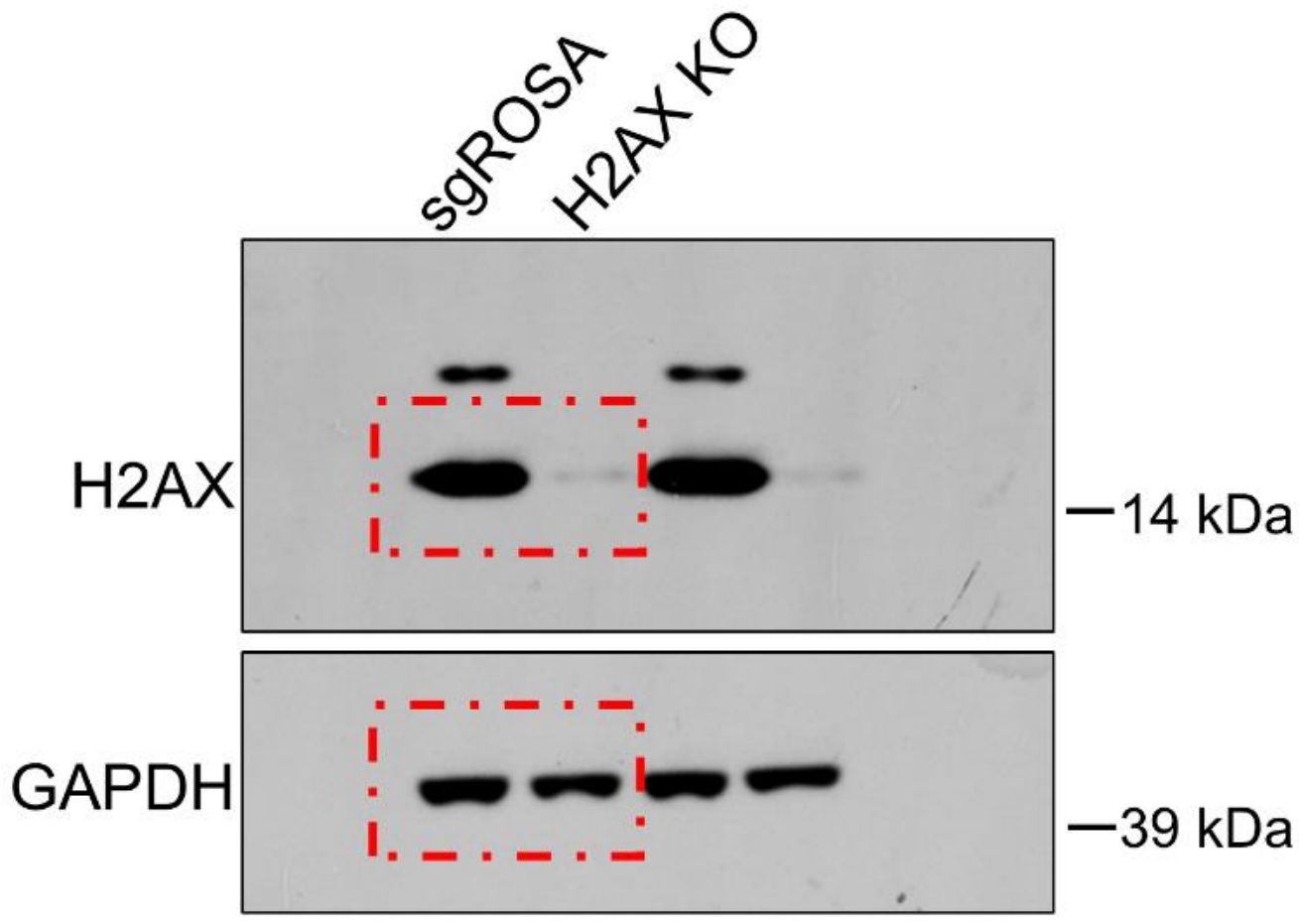

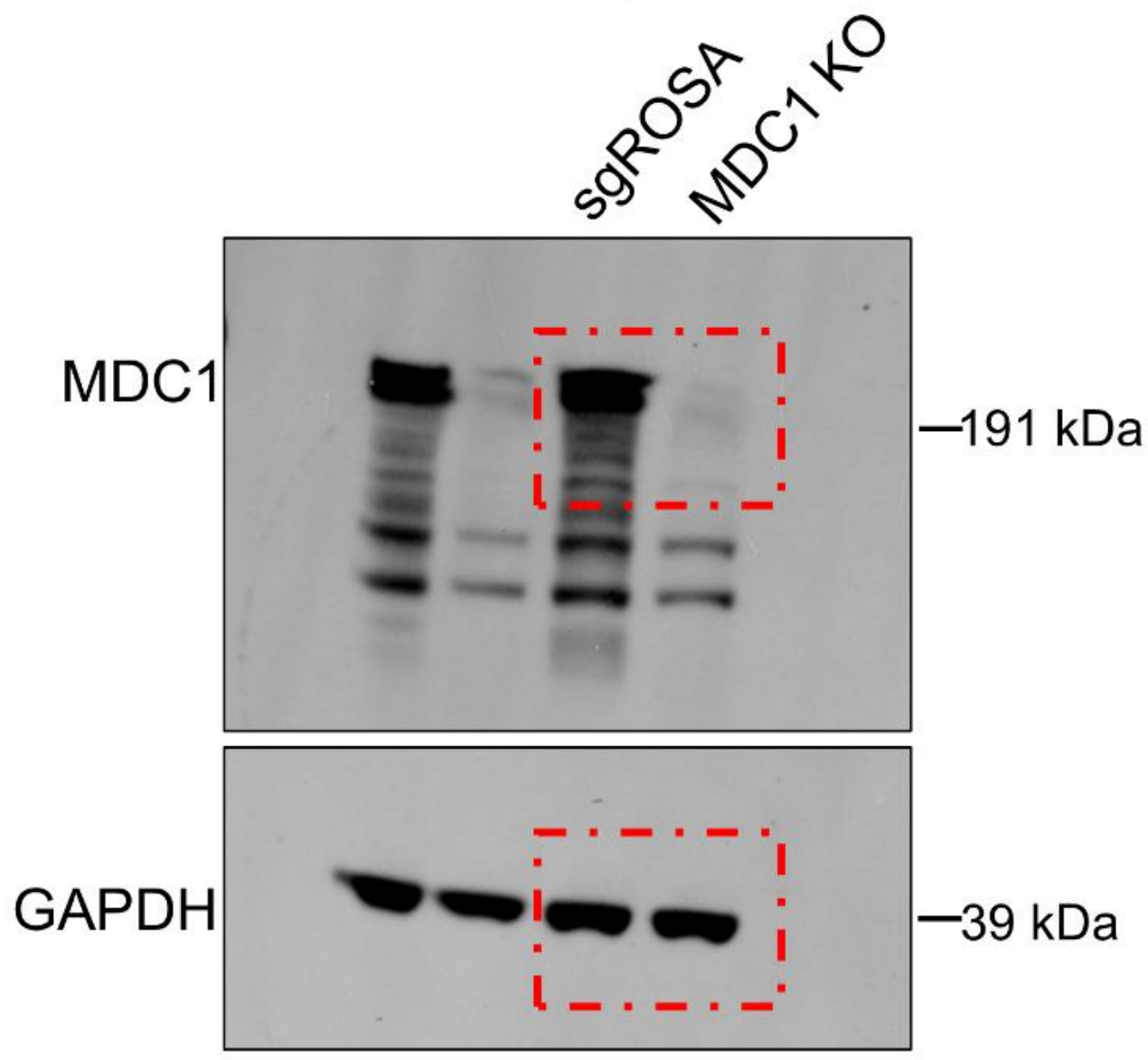

Uncropped blots of Supplementary Fig. 10a.

Dotted outline denotes samples included in the manuscript.

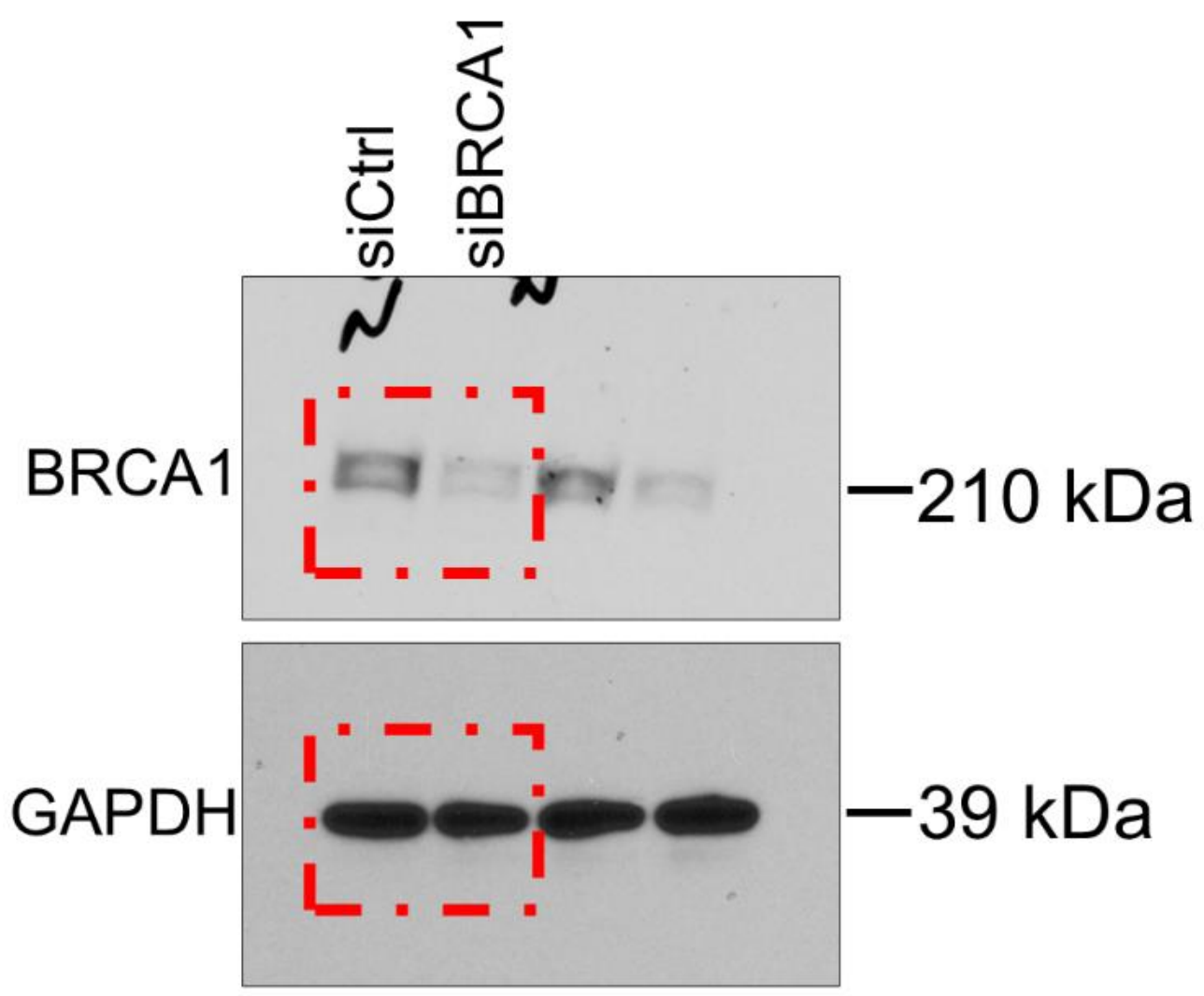

Uncropped blots of Supplementary Fig. 11a

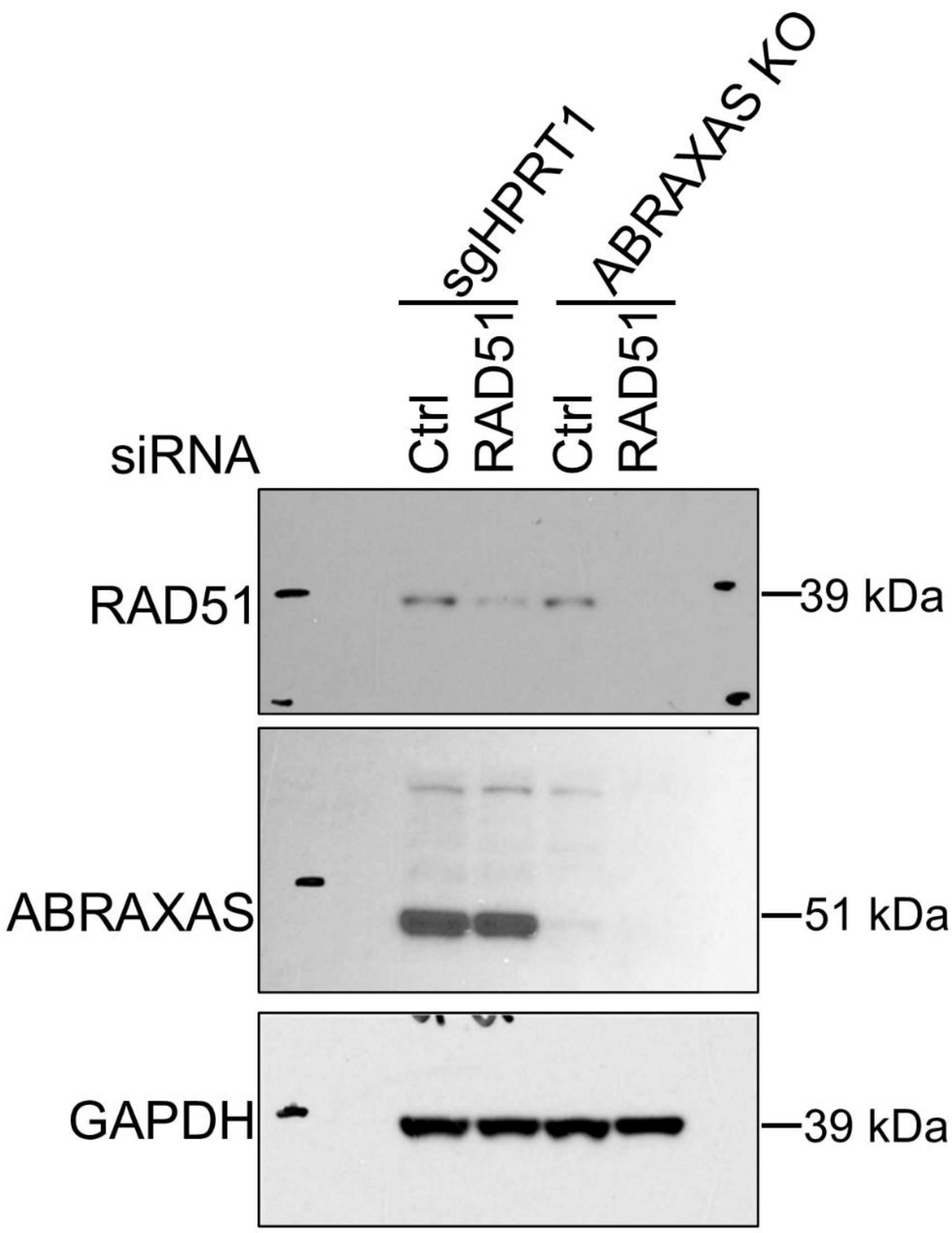

Uncropped blots of Supplementary Fig. 11d.

Dotted outline denotes samples included in the manuscript.

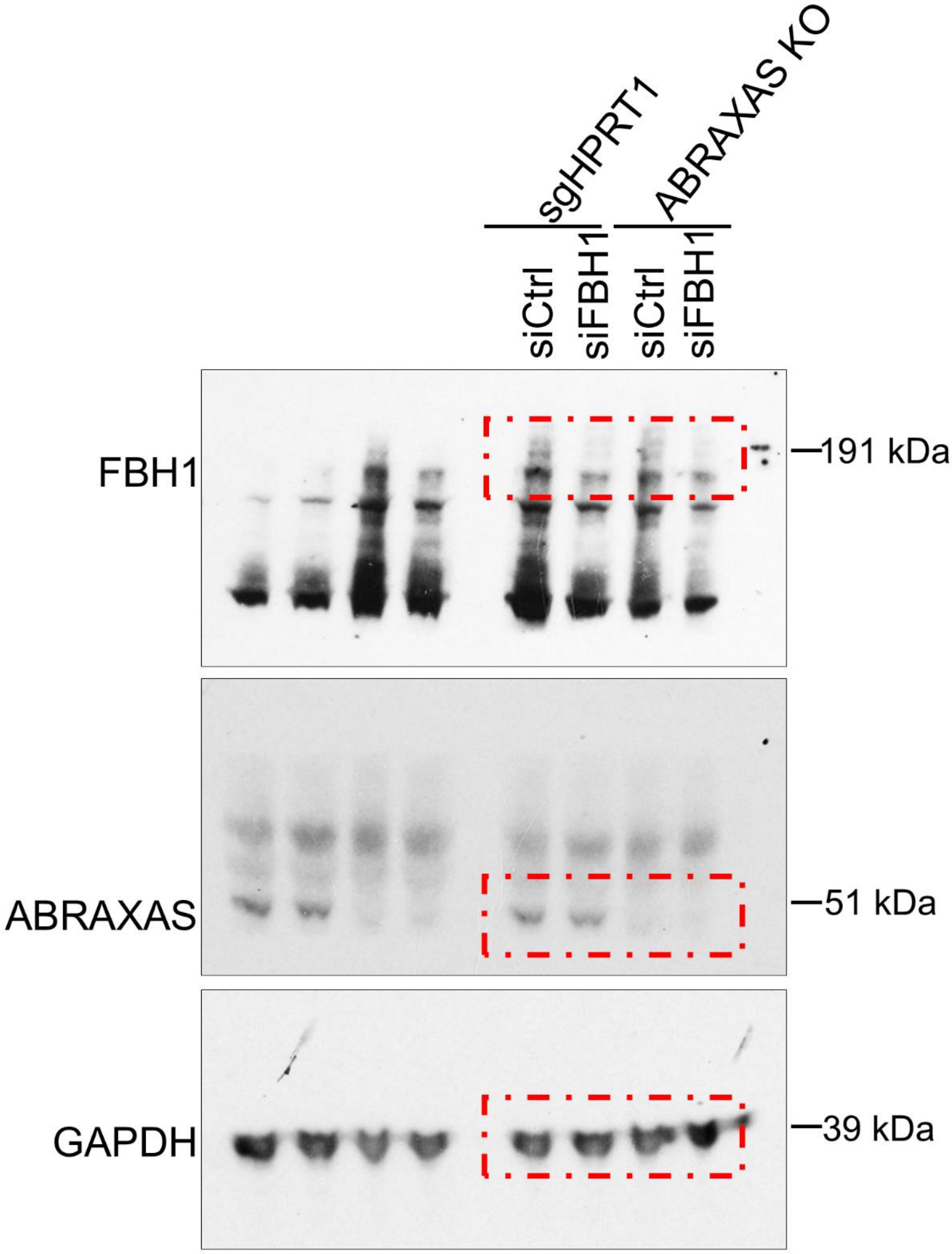

Uncropped blots of Supplementary Fig. 11e.

Dotted outline denotes samples included in the manuscrip

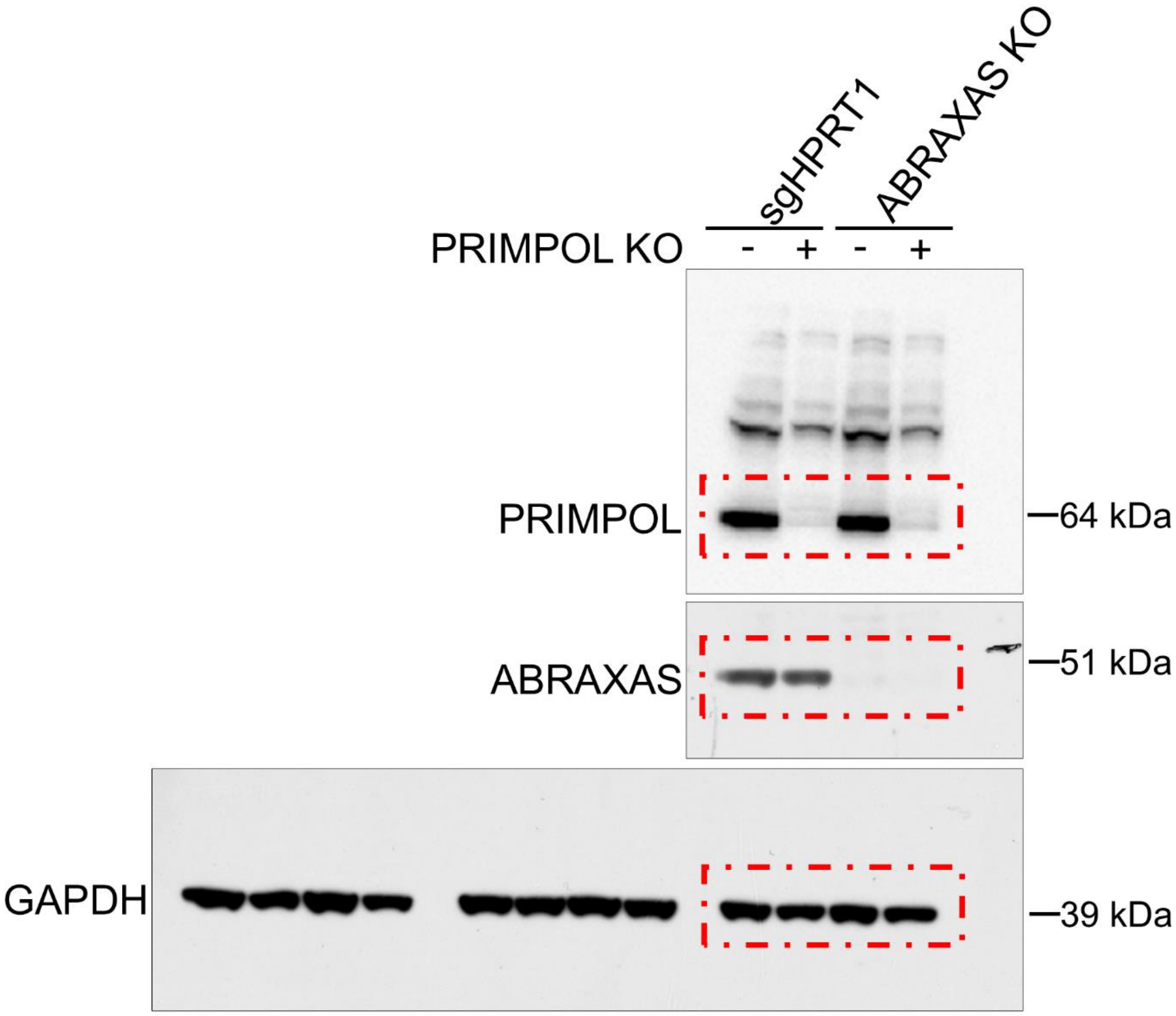

Supplement: Supplementary file 1 [file 41467_2026_75271_MOESM1_ESM.pdf]
