# Supplementary material for: The BRCA1-A complex restricts replication fork reversal-dependent DNA repair in ATM deficient cells
Source: Nat Commun. 2026 Jul 4;17:8321. doi: 10.1038/s41467-026-75271-7 (PMC13470386; doi:10.1038/s41467-026-75271-7)
Supplement: Supplementary file 2 [file 41467_2026_75271_MOESM2_ESM.pdf]

Description of Additional Supplementary Files

File Name: **Supplementary Data 1**

Description: Spectral count and intensity of all proteins identified in iPOND-MS experiments described in Fig. 2a and 2d.

File Name: **Supplementary Data 2**

Description: Name of the proteins shown in Fig. 2d. Average log2 FC in ABRAXAS KO vs sgROSA (n=5) is indicated.

File Name: **Supplementary Data 3**

Description: Gene ontology analysis data of proteins enriched by ≥ 1.20-fold in ABRAXAS KO cells over control (sgROSA) cells in iPOND-MS experiments. Analysis was done using GeneCodis 4. GeneCodis 4 uses one-sided hypergeometric distribution test and Wallenius noncentral hypergeometric distribution. p values are adjusted for multiple testing using Benjamini and Hochberg.
