# Supplementary material for: The BRCA1-A complex restricts replication fork reversal-dependent DNA repair in ATM deficient cells
Source: Nat Commun. 2026 Jul 4;17:8321. doi: 10.1038/s41467-026-75271-7 (PMC13470386; doi:10.1038/s41467-026-75271-7)
Supplement: Supplementary file 3 [file 41467_2026_75271_MOESM6_ESM.pdf]

nature portfolio

Corresponding author(s): Roger A. Greenberg

Last updated by author(s): Jun 16, 2026

Reporting Summary

Nature Portfolio wishes to improve the reproducibility of the work that we publish. This form provides structure for consistency and transparency in reporting. For further information on Nature Portfolio policies, see our Editorial Policies and the Editorial Policy Checklist.

Statistics

For all statistical analyses, confirm that the following items are present in the figure legend, table legend, main text, or Methods section.

n/a	Confirmed	
☐	☒	The exact sample size (*n*) for each experimental group/condition, given as a discrete number and unit of measurement
☐	☒	A statement on whether measurements were taken from distinct samples or whether the same sample was measured repeatedly
☐	☒	The statistical test(s) used AND whether they are one- or two-sided<br>*Only common tests should be described solely by name; describe more complex techniques in the Methods section.*
☒	☐	A description of all covariates tested
☒	☐	A description of any assumptions or corrections, such as tests of normality and adjustment for multiple comparisons
☐	☒	A full description of the statistical parameters including central tendency (e.g. means) or other basic estimates (e.g. regression coefficient) AND variation (e.g. standard deviation) or associated estimates of uncertainty (e.g. confidence intervals)
☐	☒	For null hypothesis testing, the test statistic (e.g. *F*, *t*, *r*) with confidence intervals, effect sizes, degrees of freedom and *P* value noted<br>*Give P values as exact values whenever suitable.*
☒	☐	For Bayesian analysis, information on the choice of priors and Markov chain Monte Carlo settings
☒	☐	For hierarchical and complex designs, identification of the appropriate level for tests and full reporting of outcomes
☒	☐	Estimates of effect sizes (e.g. Cohen's *d*, Pearson's *r*), indicating how they were calculated

Our web collection on statistics for biologists contains articles on many of the points above.

Software and code

Policy information about availability of computer code

Data collection	NIS-Elements BR 4.50.00 64-bit in Nikon Eclipse 80i fluorescence microscope<br>NIS-Elements AR 5.42.06 64-bit in Nikon Eclipse Ti2 fluorescence microscope<br>JEOL JEM-1400 electron microscope for EM image acquisition<br>Velos Orbitrap Elite ion-trap mass spectrometer (Thermo Fisher Scientific, Waltham, MA) for Mass spectrometry<br>Sequest (Thermo Fisher Scientific, Waltham, MA) for protein identification in Mass spectrometry
Data analysis	GraphPad Prism 10; GeneCodis 4; MS Excel Version 2605 Build 16.0.20026.20166) 64-bit; CellProfiler 4.2.5; ImageJ (https://imagej.net/ij/), MS PowerPoint Version 2605 Build 16.0.20026.20166) 64-bit

For manuscripts utilizing custom algorithms or software that are central to the research but not yet described in published literature, software must be made available to editors and reviewers. We strongly encourage code deposition in a community repository (e.g. GitHub). See the Nature Portfolio guidelines for submitting code & software for further information.

Data

Policy information about availability of data

All manuscripts must include a data availability statement. This statement should provide the following information, where applicable:

- Accession codes, unique identifiers, or web links for publicly available datasets
- A description of any restrictions on data availability
- For clinical datasets or third party data, please ensure that the statement adheres to our policy

All data are available within the article and its supplementary information files. The iPOND mass spectrometry proteomics data have been deposited to the ProteomeXchange Consortium via the PRIDE partner repository with the dataset identifier PXD077767 (https://www.ebi.ac.uk/pride/archive/projects/PXD077767).

Research involving human participants, their data, or biological material

Policy information about studies with human participants or human data. See also policy information about sex, gender (identity/presentation), and sexual orientation and race, ethnicity and racism.

Reporting on sex and gender	N/A
Reporting on race, ethnicity, or other socially relevant groupings	N/A
Population characteristics	N/A
Recruitment	N/A
Ethics oversight	N/A

Note that full information on the approval of the study protocol must also be provided in the manuscript.

Field-specific reporting

Please select the one below that is the best fit for your research. If you are not sure, read the appropriate sections before making your selection.

☒ Life sciences ☐ Behavioural & social sciences ☐ Ecological, evolutionary & environmental sciences

For a reference copy of the document with all sections, see nature.com/documents/nr-reporting-summary-flat.pdf

Life sciences study design

All studies must disclose on these points even when the disclosure is negative.

Sample size	No statistical analysis was done to pre-determine sample size for the experiments. Sample size were consistent with published studies on DNA damage repair field (Verma et al, Nature Cell Biology 2021; Gaggioli et al, Nature Cell Biology 2023; Dungrawala et al, Molecular Cell 2021; Jiang et al, JCB 2021). Sample sizes are described in the Figure Legends.
Data exclusions	In FIg. 1b, CPT concentration of 1.5 nM was not included to keep the dose curve consistent.<br>CPT concentration of 40 nM and 50 nM was not included as no colonies were formed at >20 nM CPT concentration.
Replication	Experiments were repeated independently at least two to four times with similar results. The number of replicates performed for each experiment is indicated in the Figure Legends. All results were reproducible.
Randomization	The nature of our study which involves cell-based experiments does not require any randomization due to relatively small sample size.
Blinding	Blinding to group allocation was performed for some of the immunofluorescence and colony survival data analyses, and for all EM analyses. However, it was not performed for many of the Western blotting, microscope imaging, DNA fiber experiments, cell survival assays as researchers were aware of the respective experimental conditions.

Reporting for specific materials, systems and methods

We require information from authors about some types of materials, experimental systems and methods used in many studies. Here, indicate whether each material, system or method listed is relevant to your study. If you are not sure if a list item applies to your research, read the appropriate section before selecting a response.

Materials & experimental systems

n/a	Involved in the study
☐	☒ Antibodies
☐	☒ Eukaryotic cell lines
☒	☐ Palaeontology and archaeology
☒	☐ Animals and other organisms
☒	☐ Clinical data
☒	☐ Dual use research of concern
☒	☐ Plants

Methods

n/a	Involved in the study
☒	☐ ChIP-seq
☒	☐ Flow cytometry
☒	☐ MRI-based neuroimaging

Antibodies

Antibodies used

Antibodies for Western blotting

Mouse PCNA(PC10) Cell Signaling Technology Cat# 2586 1:1000
Rabbit Histone H3 Cell Signaling Technology Cat# 9715 1:1000
Mouse anti-BrdU(B44) BD biosciences Cat# 347580 1:200
Mouse MRE11 GeneTex Cat# GTX70212 1:1000
Rabbit EXO1 GeneTex Cat# GTX109891 1:1000
Rabbit ABRAXAS Fortis Life Sciences Cat# A302-180A 1:1000
Rabbit RAP80 (D1T6Q) Cell Signaling Technology Cat# 14466 1:1000
Mouse RAD51 [14B4] GeneTex Cat# GTX70230 1:1000
Rabbit anti-Phospho KAP-1 (S824) Fortis Life Sciences Cat# A300-767A 1:1000
Mouse γH2AX (Ser139), clone JBW301 Millipore Sigmma Cat# 05-636 1:1000
Rabbit GAPDH (14C10) Cell Signaling Technology Cat# 2118 1:5000
Rabbit PRIMPOL Proteintech Cat# 29824-1-AP 1:1000
Rabbit ATM (D2E2) Cell Signaling Technology Cat#2873 1:1000
Mouse RNF8 (B-2) Santa Cruz Biotechnology sc-271462 1:1000
Rabbit RNF168 Millipore Sigma ABE367 1:1000
Rabbit H2AX Fortis Life Sciences A300-083A 1:1000
Rabbit MDC1 Abcam ab11169 1:1000
Mouse FBH1 (2353C1a) Abcam ab58881 1:250
HRP-conjugated anti-mouse secondary antibody Amersham Cat# NA931 1:3000
HRP-conjugated anti-rabbit secondary antibody Amersham Cat# NA934 1:3000

Antibodies for Immunofluorescence

Mouse RPA, clone RPA34-20 Millipore Sigma Cat# MABE285 1:1000
Rat Rpa32/rpa2 (4E4) Cell Signaling Technology Cat #2208S 1:1000
Rabbit γH2AX (Ser139) (20E3) Cell Signaling Technology Cat #9718 1:1000
Rabbit γH2AX (Ser139) Active Motif Cat# 39117 1:1000
Mouse γH2AX (Ser139), clone JBW301 Millipore Sigma Cat# 05-636 1:1000
Mouse MRE11 GeneTex Cat# GTX70212 1:100
Rabbit EXO1 GeneTex Cat# GTX109891 1:200
Rabbit RAP80 (D1T6Q) Cell Signaling Technology Cat# 14466 1:200
Mouse BRCA1 (D-9) Santa Cruz Biotechnology Cat# sc-6954 1:100
Mouse anti-HA.11 Epitope Tag BioLegend Cat# 901516 1:500
Mouse anti-Ubiquitin (FK2) Millipore Sigma Cat#04-263 1:100
Mouse Sumo 2 + Sumo 3 [8A2] Abcam Cat# ab81371 1:100
Rabbit anti-biotin (D5A7) Cell Signaling Technology Cat# 5597 1:200
Mouse anti-biotin Millipore Sigma Cat# SAB4200680 1:100
Mouse RNF8 (B-2) Santa Cruz Biotechnology sc-271462 1:100
Rabbit RNF168 Millipore Sigma ABE367 1:200
Rat anti-BrdU Abcam Cat# ab6326 1:200
Mouse anti-BrdU (B44) BD Pharmigen Cat# 47580 1:40
AlexaFluor 488-conjugated anti-mouse secondary antibody Thermo Scientific Cat# A-11029 1:500
AlexaFluor 647-conjugated anti-rat secondary antibody Thermo Scientific Cat# A-21247 1:500
AlexaFluor 647-conjugated anti-rabbit secondary antibody Thermo Scientific Cat# A-21245 1:500

Validation

Primary antibodies for Western blotting

Mouse PCNA(PC10) Cell Signaling Technology Cat# 2586: This antibody is widely used to detect PCNA and cited in >700 publications.
Rabbit Histone H3 Cell Signaling Technology Cat# 9715: This antibody is widely used to detect PCNA and cited in >1300 publications.
Mouse anti-BrdU(B44) BD biosciences Cat# 347580: This is a gold-standard antibody to detect IdU-labeled DNA in DNA fiber assay.
(Quinet A and Vindigni A., Methods Enzymol. 2017; Taglialatela and Ciccia, Mol Cell 2017). The antibody has been successfully used for native BrdU/ IdU (Couch and Cortez D., Genes Dev. 2013; Iannascoli C et al., Nucleic Acids Res. 2015) and BrdU-coupled MNase

assays (Zhu C et al Nat Commun. 2022).
Mouse MRE11 GeneTex Cat# GTX70212: KO validated by Western blotting in this study, Supplementary Fig. 6a.
Rabbit EXO1 GeneTex Cat# GTX109891: KO validated by Western Blotting in this study, Supplementary Fig. 6b.
Rabbit ABRAXAS Fortis Life Sciences Cat# A302-180A: KO validated by Western Blotting in this study, Supplementary Fig. 4a, 5b.
Rabbit RAP80 (D1T6Q) Cell Signaling Technology Cat# 14466: KO validated by Western Blotting in this study, Fig. 4b.
Mouse RAD51 [14B4] GeneTex Cat# GTX70230: KD validated by Western Blotting in this study, Supplementary Fig. 11a.
Rabbit anti-Phospho KAP-1 (S824) Fortis Life Sciences Cat# A300-767A: Widely used antibody with >200 citations. Detected ATM-dependent KAP-1 (S824) phosphorylation upon CPT treatment.
Mouse γH2AX (Ser139), clone JBW301 Millipore Sigma Cat# 05-636: This is a gold-standard antibody to detect γH2AX (Ser139) in the DNA repair field with >300 citations.
Rabbit GAPDH (14C10) Cell Signaling Technology Cat# 2118: This antibody is widely used as Western blotting loading control with >2000 citations.
Rabbit PRIMPOL Proteintech Cat# 29824-1-AP: KO validated by Western Blotting in this study Supplementary Fig. 11e.
ATM (D2E2) Cell Signaling Technology Cat#2873: KO validated by Western Blotting in this study Supplementary Fig. 1c.
Mouse RNF8 (B-2) Santa Cruz Biotechnology Cat# sc-271462: KO validated by Western Blotting in this study Supplementary Fig. 9a.
Rabbit RNF168 Millipore Sigma Cat# ABE367 : KO validated by Western Blotting in this study Supplementary Fig. 9a.
Rabbit H2AX Fortis Life Sciences Cat# A300-083A: KO validated by Western Blotting in this study Supplementary Fig. 9c.
Rabbit MDC1 Abcam Cat# ab11169: KO validated by Western Blotting in this study Supplementary Fig. 9c.
Mouse FBH1 (2353C1a) Abcam Cat# ab58881: KO validated by Western Blotting in this study Supplementary Fig. 11d.

Primary antibodies for Immunofluorescence

Mouse RPA, clone RPA34-20 Millipore Sigma Cat# MABE285: This antibody is widely used antibody in the DNA repair field with >35 citations.
Rat Rpa32/rpa2 (4E4) Cell Signaling Technology Cat# 5597 Cat #2208S: This antibody is widely used to detect RPA by immunofluorescence with 165 citations
Rabbit γH2AX (Ser139) (20E3) Cell Signaling Technology Cat #9718: This antibody is widely used antibody in the DNA repair field with >9500 citations.
Rabbit γH2AX (Ser139) Active Motif Cat# 39117: Validated as damaged marker by Active Motif: https://www.activemotif.com/catalog/details/39117/histone-h2a-xs139ph-antibody-pab-h2ax and cited in 8 publications. Detected CPT-induced γH2AX (Ser139) signal in Western blotting and immunofluorescence in the present study, Supplementary Fig. 2a.
Mouse γH2AX (Ser139), clone JBW301 Millipore Sigma Cat# 05-636, Validated by IF in this study, Supplementary Fig. 3a.
Mouse MRE11 GeneTex Cat# GTX70212: Validated by EdU PLA in this study, Fig. 3c.
Rabbit EXO1 GeneTex Cat# GTX109891: KValidated by EdU PLA in this study, Fig. 3a.
Mouse BRCA1 (D-9) Santa Cruz Biotechnology Cat# sc-6954: Validated by damage-induced BRCA1 immunofluorescence foci formation by Santa Cruz Biotechnology: https://www.scbt.com/p/brca1-antibody-d-9?srsltid=AfmBOorEiOrr5cd8lUeDuIg217AgB3q7zixga0vjKwbO5Lt0lGyzzchr with 11 citations.
Mouse anti-HA.11 Epitope Tag BioLegend Cat# 901516: Validated by overexpression in immunofluorescence in this study, Supplementary Fig. 7a.
Mouse anti-Ubiquitin(FK2) Millipore Sigma Cat#04-263: This antibody is widely used to detect polyubiquitin conjugation with >200 citations.
Mouse Sumo 2 + Sumo 3 [8A2] Abcam Cat# ab81371: This antibody is widely used to detect SUMO2/3 conjugation with 69 citations.
Rabbit anti-biotin (D5A7) Cell Signaling Technology Cat# 5597 and Mouse anti-biotin Millipore Sigma Cat# SAB4200680: These two are standard anti-biotin antibodies used for SIRF-PLA assay (Roy S and Schlacher K., JCB 2018; Roy S and Schlacher K., BioProtoc. 2019).
Mouse RNF8 (B-2) Santa Cruz Biotechnology sc-271462: Validated by EdU PLA in this study, Supplementary Fig. 9c.
Rabbit RNF168 Millipore Sigma ABE367: Validated by EdU PLA in this study, Supplementary Fig. 9d.
Rat anti-BrdU Abcam Cat# ab6326: This is a standard antibody to detect CldU-labeled DNA in DNA fiber assay (Quinet A and Vindigni A., Methods Enzymol. 2017; Quinet A and Vindigni A. Mol Cell. 2021) srsltid=AfmBOoq0ZJvT57lMk8bOjJ8wogLTS5obxbOTGXv1nsGxi72ZSkKJ1u6R. Cited in >80 publications.
Mouse anti-BrdU (B44) BD Pharmigen Cat# 47580: This is a standard antibody to detect IdU-labeled DNA in DNA fiber assay (Quinet A and Vindigni A., Methods Enzymol. 2017; Quinet A and Vindigni A. Mol Cell. 2021)

Eukaryotic cell lines

Policy information about cell lines and Sex and Gender in Research

Cell line source(s)	HT-29 (HTB-38, ATCC) and HEK293T (CRL-3216, ATCC) were purchased from ATCC.<br>SMARCAL1, ZRANB3, HLTF or FBH1 KO U2OS cell lines were provided by David Cortez, Vanderbilt University School of Medicine.
Authentication	HT-29 and HEK293T cell lines were authenticated by STR profiling at ATCC.<br>KO of SMARCAL1, ZRANB3, HLTF or FBH1 in U2OS cells were verified by PCR and immunoblotting (W. Liu and David Cortez, Sci. Adv. 2020)
Mycoplasma contamination	Cell lines were tested negative for mycoplasma using MycoStrip - Mycoplasma Detection Kit, Invivogen
Commonly misidentified lines<br>(See ICLAC register)	No commonly misidentified cell lines were used in this study

Plants

Seed stocks	N/A
Novel plant genotypes	N/A
Authentication	N/A
