# Supplementary material for: The BRCA1-A complex restricts replication fork reversal-dependent DNA repair in ATM deficient cells
Source: Nat Commun. 2026 Jul 4;17:8321. doi: 10.1038/s41467-026-75271-7 (PMC13470386; doi:10.1038/s41467-026-75271-7)
Supplement: Supplementary file 4 [file 41467_2026_75271_MOESM7_ESM.pdf]

nature portfolio

Peer Review File

The BRCA1-A complex restricts replication fork reversal-dependent DNA repair in ATM deficient cells

Corresponding Author: Dr Roger Greenberg

This manuscript has been previously reviewed at another journal. This document only contains information relating to versions considered at Nature Communications.

This file contains all reviewer reports in order by version, followed by all author rebuttals in order by version.

Version 0:

Reviewer comments:

Reviewer #1

(Remarks to the Author)
This study investigates the role of BRCA1-A during CPT-induced replicative stress in the context of ATM deficiency. Homologous recombination (HR) processes are known to be critical in response to CPT-mediated damage, which induces damage during replication and fork reversal. As one of the main sensor kinases of the DNA damage response pathway, loss of ATM results in DNA damage hypersensitivity and cancer susceptibility. Previous work on ATM-deficient cells showed that loss of NHEJ factors or of the BRCA1-A complex confers resistance to DNA-damaging agents, suggesting that their repair functions in a normal context can end up compromising repair or fork protection mechanisms upon ATM deficiency. Here, the authors show an increased BRCA1-A localization at damaged forks in the absence of ATM, mediated through both SUMO and ubiquitin recognition. They correlate this with higher chromatin compaction. They also establish that ATM inhibition (ATMi) drastically reduces the number of reversed replicative forks that typically arise in response to CPT, a phenomenon rescued by BRCA1-A loss. They propose a model where BRCA1-A increased presence in the absence of ATM impedes access of other repair factors to the forks and protective fork reversal establishment. Without reversal, 1/nucleases get less access to blocked forks, thereby hindering controlled resection and compromising repair by HR ; and 2/forks become more prone to restart via PRIMPOL and engage in toxic fusions via NHEJ. Overall, the work is technically sophisticated, addresses an important question, and contains potentially impactful findings. In particular, the identification of a functional interplay between ATM signaling and BRCA1-A at damaged replication forks is conceptually interesting and may have important implications for understanding resistance mechanisms in ATM-deficient tumors. However, there are a few concerns that authors should address before the manuscript can be considered to acceptance.

Key concerns:

1/ The introduction would benefit from a clearer explanation of the canonical DNA Damage signaling pathway involving ATM, MDC1, 53BP1 and CHK2 involvement here. Particularly, MDC1 is recruited through γ-H2AX to chromatin, additional ATM-mediated MDC1 phosphorylation recruits RNF8 and Ubc13, who finally deposit the ubiquitin residues onto H2A. Those same Ubiquitin residues are the ones known to be bound by BRCA1-A through RAP80 (PMID: 18550271). In the manuscript, authors show higher SUMO and ubiquitin abundance at forks upon ATMi in Extended Data Fig7 and point to another independent mechanism. I suggest adding the following citation and elaborating on this aspect (PMID: 35717336). However, authors also show fork ubiquitylation decrease upon RNF8 or RNF168 KO, meaning those ligases are still implicated in the process.
Given that enhanced BRCA1-A recruitment in ATM-deficient cells is central to the proposed model, the authors should clarify which components of the canonical recruitment pathway remain active and which are bypassed or modified upon ATM inhibition. Although a complete dissection of this pathway may be beyond the scope of the current manuscript, a clearer explanation and more careful interpretation would substantially improve the significance of the findings.

2/ Line 99 citations should be reassessed: n.36 shows deficient mice are hypersensitive to IR and prone to tumorigenesis, which is the opposite of what is stated in the text. N.35 shows hypersensitivity to DNA ICLs, as well as reduced end-resection and HR, conflicting with next sentence and references.

3/ It is confusing whether immunofluorescence data (like in Fig1c,d) are representing total number of foci per positive cell, spot intensity per positive cell or overlapping foci. Please extend figure legends to clarify this aspect. When first mentioned, it would be important to explain how γH2AX positive cells were assessed, especially because such signals can 1/ appear outside of the S-phase and 2/ be greatly modified by the presence of ATMi.

4/ It is understandable that all differences (p-values) between datasets cannot be shown on graphs for readability concerns, but those that shape a conclusion within the text should always appear. In this regard, please show p-value difference between S404A/S406A +/- ATMi, which you stated "[leads] to a moderate increase", line 185.

5/ More controls (untreated, +CPT alone in some mutants) would have strengthened the study, and this reviewer would like to advise including more of them in your future experimental designs. Within this study, the ABRAXAS KO +CPT without ATMi in Figure 1g should be specifically included.

6/ The data convincingly demonstrate reduced MNase accessibility of nascent chromatin in ABRAXAS-deficient cells. The increased abundance of di- and polynucleosomal BrdU-containing fragments following MNase digestion is consistent with altered chromatin organization and reduced accessibility of nascent chromatin (Fig2g). However, these data do not directly establish increased nucleosome occupancy or chromatin compaction, as MNase resistance can also be influenced by nucleosome spacing, chromatin maturation, protein occupancy such as RAD51 coated intermediates or protein enriched stalled forks, and altered replication intermediate structures ex-reversed forks, ssDNA-containing structures, branched DNA intermediates, and Holliday junction-like structures may all affect nuclease accessibility. Given that the authors themselves propose increased fork reversal in ABRAXAS-deficient cells, it remains possible that the observed reduction in MNase digestion reflects changes in replication fork architecture rather than a bona fide chromatin compaction phenotype. Thus, the current data support altered accessibility of nascent chromatin but do not convincingly demonstrate chromatin compaction per se. The authors should therefore either temper their conclusions or provide additional orthogonal evidence before proposing that BRCA1-A directly regulates chromatin compaction or higher-order chromatin architecture at stressed replication forks e.g., H3K9me2/3, histone acetylation levels, nucleosome occupancy (total H3/H4 levels), chromatin profiling approaches (CUT&RUN/CUT&Tag), or high-resolution imaging of chromatin organization.

7/ In many occasions, the authors use the term "suppress" or "rescued" for partial recovery of a phenotype, "reduces" or "decreases" would be more adequate. (lines 201, 339)
8/ Several figures cannot be assessed properly (Fig1d, 4c,g,h, 6b) because the scales are set up to include some outliers, which makes the majority of the data appear on less than 30% of the figure. This visually decreases differences between datasets.

Minor concerns :

1/ Line 220, citation 62 would benefit being updated to a non-DSB context, as it has been stressed out that lower doses of CPT (similar to those used in clinc) do not actually induce DSBs. ATM kinase activity on chromatin compaction or relaxation during replicative stress would be more adequate.
2/ Line 68 substoichiometric is missing the r
3/ Figure 3e is missing the KO after EXO1 to make it consistent with MRE11
4/ Figure 4d legend is shifted, and thus CPT+ATMi does not align with the cell lines
5/ Line 270, the authors refer to Extended Data Fig 7b and 7c instead of 8.

Reviewer #2

(Remarks to the Author)
The authors did a great job in addressing the reviewers' comments and should be congratulated for a thoroughly revised manuscript. I endorse publication in Nature Communications.

Minor suggestions to check the wording:
Lines 88-90 (check grammar)
Lines 435-437 (please check the note on condensed chromatin, now that the H3K9me3 SIRF-PLA data have been omitted)

Reviewer #3

(Remarks to the Author)
The authors have done an excellent job addressing all comments. The new experimental data provide compelling evidence for a key role of the BRCA1-A complex in suppressing fork reversal in ATM-deficient cells, thereby causing sensitivity to camptothecin. Conversely, loss of the BRCA1-A complex in ATM-deficient cells restores fork reversal, enabling DNA repair and rendering cells resistant to camptothecin treatment. Overall, the data support the model presented in Figure 7g. In my opinion, the extensively revised manuscript is acceptable for publication.

Reviewer #4

(Remarks to the Author)
I co-reviewed this manuscript with one of the reviewers who provided the listed reports. This is part of the Nature

Communications initiative to facilitate training in peer review and to provide appropriate recognition for Early Career Researchers who co-review manuscripts.

Open Access This Peer Review File is licensed under a Creative Commons Attribution 4.0 International License, which permits use, sharing, adaptation, distribution and reproduction in any medium or format, as long as you give appropriate credit to the original author(s) and the source, provide a link to the Creative Commons license, and indicate if changes were made.
In cases where reviewers are anonymous, credit should be given to 'Anonymous Referee' and the source.
The images or other third party material in this Peer Review File are included in the article's Creative Commons license, unless indicated otherwise in a credit line to the material. If material is not included in the article's Creative Commons license and your intended use is not permitted by statutory regulation or exceeds the permitted use, you will need to obtain permission directly from the copyright holder.
To view a copy of this license, visit https://creativecommons.org/licenses/by/4.0/

Reviewer #1 (Remarks to the Author):

This study investigates the role of BRCA1-A during CPT-induced replicative stress in the context of ATM deficiency. Homologous recombination (HR) processes are known to be critical in response to CPT-mediated damage, which induces damage during replication and fork reversal. As one of the main sensor kinases of the DNA damage response pathway, loss of ATM results in DNA damage hypersensitivity and cancer susceptibility. Previous work on ATM-deficient cells showed that loss of NHEJ factors or of the BRCA1-A complex confers resistance to DNA-damaging agents, suggesting that their repair functions in a normal context can end up compromising repair or fork protection mechanisms upon ATM deficiency. Here, the authors show an increased BRCA1-A localization at damaged forks in the absence of ATM, mediated through both SUMO and ubiquitin recognition. They correlate this with higher chromatin compaction. They also establish that ATM inhibition (ATMi) drastically reduces the number of reversed replicative forks that typically arise in response to CPT, a phenomenon rescued by BRCA1-A loss. They propose a model where BRCA1-A increased presence in the absence of ATM impedes access of other repair factors to the forks and protective fork reversal establishment. Without reversal, 1/nucleases get less access to blocked forks, thereby hindering controlled resection and compromising repair by HR ; and 2/forks become more prone to restart via PRIMPOL and engage in toxic fusions via NHEJ. Overall, the work is technically sophisticated, addresses an important question, and contains potentially impactful findings. In particular, the identification of a functional interplay between ATM signaling and BRCA1-A at damaged replication forks is conceptually interesting and may have important implications for understanding resistance mechanisms in ATM-deficient tumors. However, there are a few concerns that authors should address before the manuscript can be considered to acceptance.

We thank the reviewer for finding our study impactful.

Key concerns:

1/ The introduction would benefit from a clearer explanation of the canonical DNA Damage signaling pathway involving ATM, MDC1, 53BP1 and CHK2 involvement here. Particularly, MDC1 is recruited through γ-H2AX to chromatin, additional ATM-mediated MDC1 phosphorylation recruits RNF8 and Ubc13, who finally deposit the ubiquitin residues onto H2A. Those same Ubiquitin residues are the ones known to be bound by BRCA1-A through RAP80 (PMID: 18550271). In the manuscript, authors show higher SUMO and ubiquitin abundance at forks upon ATMi in Extended Data Fig7 and point to another independent mechanism. I suggest adding the following citation and elaborating on this aspect (PMID: 35717336). However, authors also show fork ubiquitylation decrease upon RNF8 or RNF168 KO, meaning those ligases are still implicated in the process.

Given that enhanced BRCA1-A recruitment in ATM-deficient cells is central to the proposed model, the authors should clarify which components of the canonical recruitment pathway remain active and which are bypassed or modified upon ATM inhibition. Although a complete dissection of this pathway may be beyond the scope of the current manuscript, a clearer explanation and more careful interpretation would substantially improve the significance of the findings.

In the revised manuscript, we delineated that BRCA1-A recruitment to damaged forks in ATM inhibited cells relies on a combination of SUMOylation and RNF8/ RNF168 dependent fork ubiquitylation. While we appreciate the reviewer's thought on delineating canonical DSB response, we believe that further elaborating on this aspect will cause the manuscript to deviate from its focus

on replication fork damage which elicits a mechanistically distinct response from canonical two-ended DSBs.

We have added PMID: 35717336 in the discussion section while including potential roles of ATM at damaged replication forks.

2/ Line 99 citations should be reassessed: n.36 shows deficient mice are hypersensitive to IR and prone to tumorigenesis, which is the opposite of what is stated in the text. N.35 shows hypersensitivity to DNA ICLs, as well as reduced end-resection and HR, conflicting with next sentence and references.

One of these studies (Ref #35 and #36) found no change in IR sensitivity in vivo and the other described very modest changes. Importantly, resection was reduced only during crosslink repair because A complex deficiency resulted in a delay of FAND2-I recruitment to ICL damage, hence reducing ICL unhooking and subsequent resection. This differs from the well documented anti-resection and anti HR functions of BRCA1-A as described in refs. 37-38 (Coleman and Greenberg, JBC 2011; Hu, Livingston, GenesDev 2011), which relates to the basis of our present study. We have modified the text accordingly, "A-complex deficiency is well tolerated in mice, which showed minimal to no changes in comparison to wildtype littermates in response to IR and only modest hypersensitivity to replication fork damaging agents[35, 36]"

3/ It is confusing whether immunofluorescence data (like in Fig1c,d) are representing total number of foci per positive cell, spot intensity per positive cell or overlapping foci. Please extend figure legends to clarify this aspect. When first mentioned, it would be important to explain how γH2AX positive cells were assessed, especially because such signals can 1/ appear outside of the S-phase and 2/ be greatly modified by the presence of ATMi.

We have revised the figure legends of Fig. 1d to state that the data represents total number of RPA foci per EdU positive cell. We also now explaine how γH2AX positive cells were assessed in the Methods section.

4/ It is understandable that all differences (p-values) between datasets cannot be shown on graphs for readability concerns, but those that shape a conclusion within the text should always appear. In this regard, please show p-value difference between S404A/S406A +/- ATMi, which you stated "[leads] to a moderate increase", line 185.

We have indicated the p value difference between S404A/S406A +/- ATMi in Fig. 1f.

5/ More controls (untreated, +CPT alone in some mutants) would have strengthened the study, and this reviewer would like to advise including more of them in your future experimental designs. Within this study, the ABRAXAS KO +CPT without ATMi in Figure 1g should be specifically included.

Many of our experiments include CPT only controls. Specifically, RPA foci have been examined in ABRAXAS KO cells treated with only CPT without ATMi in Fig. 1d. We also show that Abraxas KO in DMSO control cells does not confer hypersensitivity to CPT (Fig. 1a, b). We feel that these experiments are sufficient to justify not including additional experiments that examine damage responses in Abraxas KO cells without ATMi treatment.

6/ The data convincingly demonstrate reduced MNase accessibility of nascent chromatin in ABRAXAS-deficient cells. The increased abundance of di- and polynucleosomal BrdU-containing fragments following MNase digestion is consistent with altered chromatin organization and reduced accessibility of nascent chromatin (Fig2g). However, these data do not directly establish increased nucleosome occupancy or chromatin compaction, as MNase resistance can also be influenced by

nucleosome spacing, chromatin maturation, protein occupancy such as RAD51 coated intermediates or protein enriched stalled forks, and altered replication intermediate structures ex-reversed forks, ssDNA-containing structures, branched DNA intermediates, and Holliday junction-like structures may all affect nuclease accessibility. Given that the authors themselves propose increased fork reversal in ABRAXAS-deficient cells, it remains possible that the observed reduction in MNase digestion reflects changes in replication fork architecture rather than a bona fide chromatin compaction phenotype. Thus, the current data support altered accessibility of nascent chromatin but do not convincingly demonstrate chromatin compaction per se. The authors should therefore either temper their conclusions or provide additional orthogonal evidence before proposing that BRCA1-A directly regulates chromatin compaction or higher-order chromatin architecture at stressed replication forks e.g., H3K9me2/3, histone acetylation levels, nucleosome occupancy (total H3/H4 levels), chromatin profiling approaches (CUT&RUN/CUT&Tag), or high-resolution imaging of chromatin organization.

We have replaced the phrase “chromatin compaction” with chromatin accessibility in the text.

7/ In many occasions, the authors use the term “suppress” or “rescued” for partial recovery of a phenotype, “reduces” or “decreases” would be more adequate. (lines 201, 339)

We have edited the text accordingly by changing “suppress” to “limit” and “rescued” to “partially rescued”.

8/ Several figures cannot be assessed properly (Fig1d, 4c,g,h, 6b) because the scales are set up to include some outliers, which makes the majority of the data appear on less than 30% of the figure. This visually decreases differences between datasets.

We have removed the outliers in Fig1d, 4c,g,h, 6b.

Minor concerns :

1/ Line 220, citation 62 would benefit being updated to a non-DSB context, as it has been stressed out that lower doses of CPT (similar to those used in clinc) do not actually induce DSBs. ATM kinase activity on chromatin compaction or relaxation during replicative stress would be more adequate.

Reference #62 describes ATM functions specifically at canonical DSBs and not at stressed forks. Therefore, we specified that it promotes chromatin relaxation at DSBs and in the next sentence hypothesized, rather than explicitly stated as fact, that ATM would regulate chromatin accessibility at forks.

2/ Line 68 substoichiometric is missing the r

This is corrected.

3/ Figure 3e is missing the KO after EXO1 to make it consistent with MRE11

This is corrected.

4/ Figure 4d legend is shifted, and thus CPT+ATMi does not align with the cell lines

This is corrected.

5/ Line 270, the authors refer to Extended Data Fig 7b and 7c instead of 8.

This is corrected.

Reviewer #2 (Remarks to the Author):

The authors did a great job in addressing the reviewers' comments and should be congratulated for a thoroughly revised manuscript. I endorse publication in Nature Communications.

We thank the reviewer for the appreciation of our revision and endorsing publication of the study.

Minor suggestions to check the wording:

Lines 88-90 (check grammar)

Grammar is corrected.

Lines 435-437 (please check the note on condensed chromatin, now that the H3K9me3 SIRF-PLA data have been omitted)

We have reconstructed the sentence and describe a BRCA1-A dependent restrictive chromatin state at forks during ATMi. We removed language regarding “condensed chromatin”.

Reviewer #3 (Remarks to the Author):

The authors have done an excellent job addressing all comments. The new experimental data provide compelling evidence for a key role of the BRCA1-A complex in suppressing fork reversal in ATM-deficient cells, thereby causing sensitivity to camptothecin. Conversely, loss of the BRCA1-A complex in ATM-deficient cells restores fork reversal, enabling DNA repair and rendering cells resistant to camptothecin treatment. Overall, the data support the model presented in Figure 7g. In my opinion, the extensively revised manuscript is acceptable for publication.

We thank the reviewer for the appreciation of our revision and supporting publication of the study.

Reviewer #4 (Remarks to the Author):

I co-reviewed this manuscript with one of the reviewers who provided the listed reports. This is part of the Nature Communications initiative to facilitate training in peer review and to provide appropriate recognition for Early Career Researchers who co-review manuscripts.

We thank the reviewer for reviewing our manuscript.
